# Crowding-out effects of opt-out defaults: Evidence from organ donation policies

**DOI:** 10.1093/pnasnexus/pgaf311

**Published:** 2025-10-28

**Authors:** Pascal Güntürkün, Sinika Studte, Daniel Winkler, Michel Clement, Jonathan H W Tan, Eva-Maria Merz, Elisabeth Huis in ‘t Veld, Eamonn Ferguson

**Affiliations:** Department of Marketing, Vienna University of Economics and Business, 1020 Vienna, Austria; Competence Center for Experimental Research, Vienna University of Economics and Business, 1020 Vienna, Austria; Institute for Marketing, University of Hamburg, 20148 Hamburg, Germany; Department of Marketing Transformation, HSBA Hamburg School of Business Administration, 20459 Hamburg, Germany; School of Marketing, University of New South Wales, Sydney, NSW 2033, Australia; Institute for Marketing, University of Hamburg, 20148 Hamburg, Germany; Department of Economics, School of Social Sciences, Nanyang Technological University, Singapore 639798, Singapore; Department of Sociology, Vrije Universiteit Amsterdam, 1081HV Amsterdam, The Netherlands; Sanquin Blood Supply, Research and Lab Services, 1066CX Amsterdam, The Netherlands; Hamburg Institute of Advanced Studies, 20148 Hamburg, Germany; Department of Cognitive Science and Artificial Intelligence, Tilburg School of Humanities and Digital Sciences, Tilburg University, 5037AB Tilburg, The Netherlands; School of Psychology, University of Nottingham, Nottingham NG7 2RD, United Kingdom; Department of Public Health and Primary Care, National Institute for Health and Care Research, Blood and Transplant Research Unit in Donor Health and Behaviour, University of Cambridge, Cambridge, United Kingdom

**Keywords:** behavioral interventions, default nudges, public health policy, organ donation, crowding-out effects

## Abstract

Many of today's pressing societal challenges, such as organ shortages, low vaccination rates, and climate change, require significant changes in individual behavior. One promising intervention to encourage such behavioral change is the opt-out default, which presumes consent for a desirable action rather than requiring active opt-in. While past research focused on the impact of opt-out defaults on the targeted behavior, potential crowding out of related behaviors has been largely overlooked. Here, we investigate whether adopting opt-out policies for deceased organ donation reduces living donations, a related prosocial behavior serving the same public good. Analyzing epidemiological panel data from countries that adopted an opt-out default between 2000 and 2023, we find that the policy switch, on average, leads to a nonsignificant increase in annual *deceased* donor rates of +1.21 people per million population (+7%, *P* = 0.213) but to a significant decrease in *living* donor rates of −4.59 people per million population (−29%, *P* = 0.026). Across four additional studies, we demonstrate that this crowding-out effect is reflected in a reduced willingness for living altruistic (vs. familial) donations and is attributable to a stronger belief that the organ supply is sufficiently met with deceased donations under opt-out (vs. opt-in). Our research advances insights into the unintended consequences of default nudges and suggests ways to mitigate them.

- Positive Analysis of Policy Formulation and Implementation D78
- Micro-Based Behavioral Economics: Role and Effects of Psychological, Emotional, Social, and Cognitive Factors on Decision Making D910

I. Health, Education, and Welfare

- Government Policy • Regulation • Public Health I18

Significance Statement The opt-out default is one of the most promising behavioral nudges to improve individuals' health, wealth, and sustainability decisions. But can this widely used policy intervention also have unintended consequences? We study this possibility in the context of organ donations and show that opt-out defaults have a crowding-out effect on related behaviors (living donations) not targeted by the intervention, offsetting the intended welfare gains. We reveal that this is due to people holding stronger beliefs that the opt-out default has solved the organ shortage problem. Our findings are relevant for behavioral scientists and policymakers as they extend the theoretical and practical understanding of default nudges and call for further research to overcome the identified crowding-out effect.

## Introduction

Many of today's pressing societal challenges, such as the shortage of organs for transplantation, low vaccination rates, or the progression of climate change, call for significant changes in individual's behavior (1–3). One of the most promising interventions to promote such behavioral change is the opt-out default nudge, where a socially desirable behavior is set as the default that applies if one does not actively opt-out (1, 4). Introducing such an opt-out default has a strong influence on behavior as defaults convey a recommended action, establish a reference point for evaluation, and make it easy to accept the behavior (5, 6). For policymakers, the inherent appeal of adopting an opt-out policy stems from its noncoercive nature, as it does not restrict individual choice (1). Not surprisingly, opt-out default policies have been widely adopted and shown to be highly effective and cost-efficient in promoting socially desirable behaviors across various domains (2, 3, 5, 7).

One of the most prominent examples of the success of the opt-out default policy concerns organ donation. Securing a sufficient supply of organ donations is essential to saving lives. In the United States, for example, the waiting time for a kidney is 3–5 years, and every day, 17 people die while waiting for an organ transplant (8). In their seminal article, Johnson and Goldstein (9) suggested that simply switching from an opt-in default (where nobody is a donor unless they opt-in) to an opt-out default for deceased organs (where everybody who is eligible is a donor unless they opt-out) can save many lives, as countries using an opt-out default policy have, on average, six-times higher registration rates for deceased organ donation. Many other studies have since confirmed a positive effect of the opt-out default on deceased donor registration rates and actual deceased donations (for a review, see 10). Given these promising projections and the organ shortages worldwide, many countries have decided to adopt an opt-out default policy (e.g. recently England and The Netherlands (11)) or are considering a switch (e.g. Germany (12)).

However, there is yet little evidence on the total welfare gains of such a policy switch regarding total organ donors gained and, by implication, lives saved. One key reason is that prior work on opt-out defaults has primarily focused on effects on deceased donations (the targeted behavior), while the possibility of unintended negative effects on living donations (a related behavior, not targeted by the policy) has been largely overlooked (13–17). Given that ∼61% of kidney transplants worldwide come from deceased, and 39% come from living organ donors (18), a more holistic view is essential to assess the actual welfare gains of the opt-out default policy in terms of its effects on the total supply of organs for transplant from both living and deceased donors.

In this paper, we examine whether adopting an opt-out default policy leads to “crowding-out” effects on related behaviors (i.e. living donation) not targeted by the intervention. We define the opt-out policy default's crowding-out effect as its indirect negative effect on the willingness to make living organ donations and, in turn, the number of living organ donors. To estimate the effects of the policy adoption on deceased and living organ donor rates, we used a difference-in-difference approach (19) to compare relative donor rate developments between countries that switched to opt-out and countries that maintained an opt-in policy between 2000 and 2023. We find that while adopting an opt-out policy increased deceased donors in some countries, it did not lead to a significant average increase in deceased donors per million population (+1.21 pmp, *P* = 0.213). In contrast, we find consistent evidence across switching countries that the policy crowds out living organ donors, with an average decrease of −4.59 pmp (*P* = 0.026).

We hypothesize that a perceptional bias on how people perceive the supply of organs following the policy change can explain this crowding-out effect. Specifically, we predict that people will be less willing to become a living organ donor under an opt-out than an opt-in default due to stronger beliefs that organ supply is sufficient to meet demand under opt-out. This inflated perception of organ supply is likely to be triggered by the anticipation of higher registration rates under opt-out and may be reinforced by the rhetoric from politicians advocating a move to an opt-out default policy (20). Thus, people might perceive that more organs are available under the opt-out default policy and, as a result, feel less inclined to donate as living donors. We also test two alternative potential explanations for the perceived supply-sufficiency hypothesis: (i) reputation gain (studies 2 and 3) and (ii) injunctive social norms (studies 4 and 5). The detailed rationale for these alternative mechanisms is included in the relevant studies.

We also expect this crowding-out effect to be stronger for decisions about altruistic (e.g. to a friend or stranger) vs. kinship living donations (e.g. to a family member). The decision to donate to a close family member should be less susceptible to being influenced by the default policy because people are generally more willing to incur higher costs to help family members due to direct personal and genetic benefits (21–23), while the default policy more likely influences the assessment of altruistic acts (24, 25). This distinction not only promotes a deeper understanding of the limits of conditional cooperation but is also important from a clinical perspective, as altruistic donations accounted for 10% of living kidney donations in the United Kingdom in 2019–2020 (26) and 7% in the United States in 2021 (8)—a trend that is likely to increase (27).

We provide empirical evidence for these predictions in four preregistered studies: the first is a cross-country survey (*n* = 435) that compares people's perceptions about the supply of organs for transplantation and their willingness to become a living organ donor across two culturally similar countries using different default policies (i.e. Germany opt-in and Austria opt-out). This study aims to cross-validate the behavioral effect in opt-in vs. opt-out counties and show that supply sufficiency is perceived as higher under opt-out. The second is a randomized experiment (*n* = 1,721) in which we manipulate the default policy (opt-in vs. opt-out) and registration rate (low vs. high) as a behavioral proxy of the supply of deceased organs. This experiment explored if the proposed effect of supply sufficiency is driven by the default or registration rates or their combination. That is, the effect is strongest for opt-out with a high registration rate. The third is a randomized experiment (*n* = 1,582) that examines the role of organ shortages when policy and registration rates are congruent. We expect that greater perceived shortages, especially under opt-out, should lead to lower perceptions of supply sufficiency and higher willingness to donate. The fourth is a randomized experiment (*n* = 1,225) in Switzerland (a country that in 2022, based on a referendum, decided to implement opt-out but has not yet switched) that explores if exposure to a realistic description of the effectiveness of the opt-out policy (that supply of deceased donor organs is not much better than under opt-in) compared with the standard description of opt-out and an optimistic description influence perception of supply sufficiency. We expect that perceived supply sufficiency will be reduced when the effectiveness of opt-out is presented in a realistic fashion. The first two studies explore the alternative mechanism of reputation building, and the latter two study injunctive norms.

Our research makes two important contributions to the recently emerging literature on the unintended consequences of default nudges (28, 29). First, we show that adopting an opt-out default policy to increase individual altruistic giving behavior (i.e. becoming an organ donor after death) can lead to substantial crowding-out effects for related altruistic acts untargeted by the intervention (i.e. becoming an organ donor while alive). Second, we identify a mechanism related to the default nudge that, in part, explains this crowding-out effect. Specifically, we show that higher beliefs in the effectiveness of the default nudge create an inflated perception of the sufficient supply for the public good, which decreases people's willingness to engage in the related altruistic behavior.

Our findings emphasize the importance of looking beyond the target behavior and considering potential crowding-out effects on related behaviors to understand the full welfare implications of opt-out defaults. The mechanism we identify points to a dilemma for policymakers who want to advocate for implementing an opt-out default, as the stronger people believe in the effectiveness of a default, the stronger the crowding-out effects are. These findings have important implications for behavioral scientists and (health) policymakers seeking to improve the effectiveness of opt-out defaults.

## Study 1: epidemiological study using a quasi-experimental design

Study 1 examines how the adoption of opt-out default policies has influenced deceased and living organ donor rates in countries that transitioned from opt-in policies between 2000 and 2023. To examine behavioral consequences over time, we collected epidemiological data on annual numbers of actual living and deceased organ donors from the International Registry for Organ Donation and Transplantation (IRODaT; 30) and annual NHSBT reports for each of the four countries within the United Kingdom (31). The countries' organ donation policy was classified based on websites of health ministries and governments, available legislative documents, and verified with correspondence with health ministry representatives to establish accurate dates for policy changes from opt-in to opt-out (Table S1.1).

We gathered data from countries that either transitioned to an opt-out policy during the study period (i.e. treated countries) or maintained an opt-in default policy throughout (i.e. control countries). All treated countries initially adopted a “soft” opt-out policy, where family members can still override presumed consent. We also included countries that later revised and strengthened their legislation toward a “hard” opt-out model, in which family veto is no longer permitted. In total, 24 countries met our inclusion criteria: 18 maintained an opt-in policy throughout the study period, while six transitioned to an opt-out policy (Slovak Republic: January 2005, Argentina: December 2005, Wales: December 2015, England: May 2020, The Netherlands: July 2020, and Scotland: March 2021; Fig. S1.1 and Table S1.1).

**Fig. 1.**
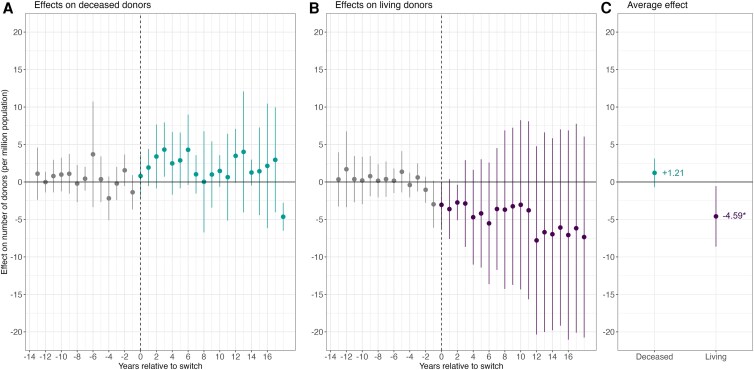
Switching to an opt-out policy has a lasting negative effect on living donor rates (study 1). Results of a difference-in-differences model with staggered treatment adoption. The vertical dashed lines in A and B reflect the time point of the policy switch. Panel C shows average annual postadoption effects. Error bars represent the 95% CI for each point estimate. Significance levels in C: * *P* < 0.05.

We use a quasi-experimental approach that compares the relative development of donor rates between countries that switched to an opt-out default and those that continued to use an opt-in default during the study period. We employ the difference-in-differences estimator proposed by Callaway and Sant’Anna (19) to address the panel data structure and variation in countries' policy adoption timing. Following prior research (13, 32), we added annual statistics on gross domestic product and road traffic fatalities from the World Bank database as covariates in the model. Figure 1 shows the average effects of adopting an opt-out default policy on deceased donors (Fig. 1A) and living donors (Fig. 1B) for a year relative to the switch. Figure 1C shows the aggregated effects across the postadoption period. Any deviation from zero in the postadoption effects can be attributed to the switch. It is interpreted as the relative difference to the counterfactual, i.e. the development of the donor rates if the country would not have switched its default policy.

The results show that switching to an opt-out default policy increased deceased donor rates in some countries (e.g. Wales), but did not yield a significant average annual increase across all switching countries (+1.21 pmp, 95% CI: −0.70, 3.12; *P* = 0.213; Table 1 and Fig. 1A and C). This marginal positive uplift of 7% for the targeted outcome aligns with recent studies examining the impact of opt-out policies on deceased organ donation rates (32). However, adopting an opt-out policy consistently crowded out living organ donors in all switching countries, reflected in a significant average annual decrease of −4.59 pmp (95% CI: −8.63, −0.55; *P* = 0.026), corresponding to a relative drop of −29% (Table 1 and Fig. 1B and C). These findings support our crowding-out hypothesis and are stable over time. We performed a series of robustness checks that consistently confirm this pattern of results (Supplementary File S1, Robustness checks).

**Table 1.** Switching to opt-out crowds out living donor rates across countries (study 1).

	Deceased donors (pmp)			Living donors (pmp)			Aggregate total effects (pmp)		
Country (cohort)	Estimate	95% CI	*P*	Estimate	95% CI	*P*	Estimate	95% CI	*P*
**ATT(Average)**	**1**.**21**	**(−0.70, 3.12)**	**0**.**213**	**−4**.**59**	**(−8.63, −0.55)**	**0**.**026**	**−3**.**38**	**(−8.37, 1.62)**	**0**.**185**
ATT(2005): Slovak Rep.	−0.67	(−2.08, 0.73)	0.346	−3.83	(−13.45, 5.79)	0.435	−4.50	(−14.45, 5.44)	0.375
ATT(2006): Argentina	5.22	(3.49, 6.96)	<0.001	−4.90	(−14.87, 5.07)	0.336	0.32	(−10.54, 11.18)	0.954
ATT(2016): Wales	4.92	(3.14, 6.70)	<0.001	−3.64	(−8.33, 1.05)	0.128	1.29	(−3.94, 6.51)	0.629
ATT(2021): England	−3.25	(−4.86, −1.64)	<0.001	−7.41	(−8.98, −5.84)	<0.001	−10.66	(−12.93, −8.39)	<0.001
ATT(2021): Netherlands	1.61	(−0.30, 3.52)	0.098	−2.20	(−3.76, −0.65)	0.006	−0.59	(−3.25, 2.06)	0.662
ATT(2021): Scotland^a^	−0.56	(−2.28, 1.16)	0.523	−5.55	(−7.18, −3.93)	<0.001	−6.11	(−8.44, −3.78)	<0.001

ATTs (average treatment effects on the treated) represent the average annual change in donor rates (pmp) following the adoption of an opt-out policy. Bold values indicate the average ATT across all switching countries. ATT(year): Country denotes country-specific ATTs, and the year in parantheses refers to the first full year of implementation. pmp = per million population.

^a^Although Scotland switched in March 2021, we treat 2021 as the first full year of implementation because the NHSBT reporting year runs from April to March.

In sum, the policy's effects on deceased and living donors largely offset each other, resulting in a small, statistically nonsignificant aggregate decrease in total donors (−3.38 pmp; 95% CI: −8.37, 1.62; *P* = 0.185). However, deceased and living donors are no substitutes as they differ in both the quantity and quality of organs donated. A deceased donor can donate two kidneys instead of one (although the effective yield is closer to 1.5–1.8 kidneys per donor; 33), which is relevant as most people on organ transplant waiting lists need a kidney (8). While a living organ donor can only give one kidney, a living donation yields more favorable patient outcomes including higher quality of life (34) and improved survival rates (35, 36). Thus, the overall welfare and ethical implications of adopting an opt-out policy are more complex than the reported statistics suggest.

Taken together, study 1 provides behavioral evidence for the hypothesized crowding-out effect of the opt-out default policy on living donor rates.

## Study 2: field-based comparison between Germany (opt-in) and Austria (opt-out)

The aim of study 2 is to cross-validate the epidemiological findings through a comparative survey between two countries with different policies and show how the perceived supply sufficiency mechanism explains crowding-out effects across familial and altruistic donation decisions. We compared perceptions of supply sufficiency and willingness to become a living organ donor between Germany (GER; opt-in) and Austria (AT; opt-out) as an example of two culturally similar countries with different default policies (9, 24). We used an online survey to collect a sample of 435 participants with representative demographic profiles from both countries (*N*_GER_ = 210; *N*_AT_ = 225; 49% female; *M*_age_ = 44.17; SD = 15.06).

We also tested reputation building as an alternative explanation to supply sufficiency. There is evidence that being a deceased organ donor is seen as a more mundane act of altruism under an opt-out (vs. opt-in) as it shifts the norm toward being a donor (24), which weakens the reputational incentives (or “kudos”) of being a living donor. We test whether this reduction in perceived reputation building generalizes to diminish peoples' willingness to become living organ donors.

Upon entering the survey, participants were briefed about the current default policy and donor registration rates in their respective countries (Germany: opt-in default, 36% registered; Austria: opt-out default, 99.5% registered) and general information on living donation. To explore differences between familial and altruistic donation decisions, we differentiate between participants' willingness to make a directed familial donation (e.g. to a child or spouse), a directed altruistic donation (to a close friend, a remote relative, and an acquaintance), and a nondirected altruistic donation (to a stranger) (23). To test our hypotheses regarding the underlying mechanism of perceived organ supply sufficiency to meet demand (e.g. “I think that the need for organs is already sufficiently covered by the number of existing donors,” 37) and perceived reputation building associated with an organ donation (e.g. “Donating organs gives me social approval,” 38). Finally, participants responded to a set of covariate items, provided demographic information, and were debriefed.

**Fig. 2.**
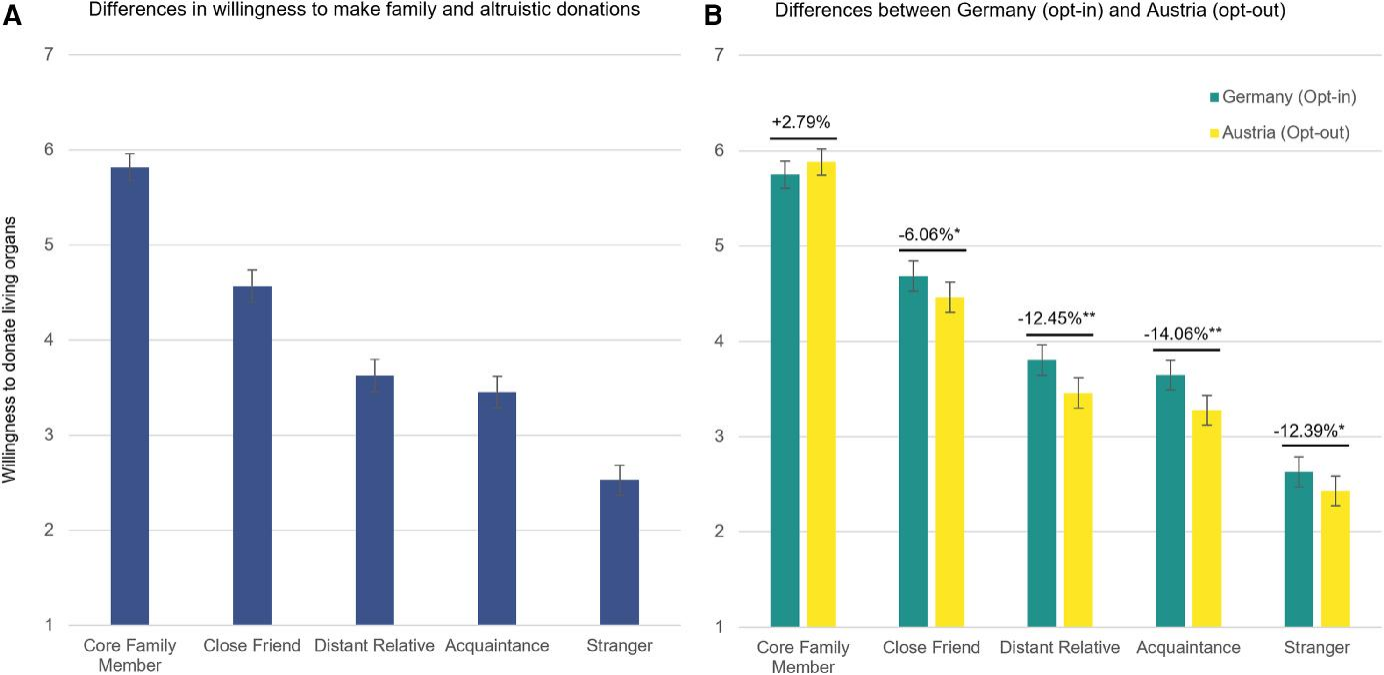
Willingness to make altruistic but not familial living organ donations is lower in Austria (opt-out) than in Germany (opt-in) (study 2). Panel A shows that participants' willingness to make familial living organ donations is generally higher than their willingness to make altruistic donations (i.e. to friends, distant relatives, acquaintances, or strangers) for the total sample (*n*_Total_ = 435). Panel B shows differences in willingness to donate between Germany (*n*_GER_ = 210) and Austria (*n*_AT_ = 225). In A, bar plots illustrate the adjusted means of participants' willingness to donate for each recipient and error bars represent 95% CIs. In B, bar plots show the adjusted point estimates for mean differences and error bars represent 95% CIs. Additional significance levels in B: *P < 0.05, **P < 0.01.

The results of a regression analysis confirm our findings from study 1 by showing that the willingness to become a living organ donor is significantly lower under an opt-out policy with 99.5% registered donors (Austria) compared with an opt-in policy with 36% registered donors (Germany; *B* = −0.312; SE = 0.129; *P* = 0.015). They further indicate that this effect is primarily driven by a decrease in people's willingness to make altruistic living organ donations under opt-out compared with opt-in. Specifically, willingness to donate to a friend (*B* = −0.317; SE = 0.160; *P* = 0.047), a distant relative (*B* = −0.466; SE = 0.162; *P* = 0.004), an acquaintance (*B* = −0.470; SE = 0.159; *P* = 0.003), and a stranger (*B* = −0.347; SE = 0.158; *P* = 0.028) is significantly lower under opt-out (Fig. 2), while people's willingness to make a kinship donation to a family member is equally high across both policy contexts (*B* = 0.038; SE = 0.140; *P* = 0.786). These results support our prediction that the crowding-out effect of the opt-out default policy on living organ donations mainly undermines altruistic donation decisions, while donations to close kin are unaffected.

Focusing on the underlying mechanism, we find that perceived sufficiency of organ supply is significantly higher among participants from a country using an opt-out (Austria) vs. an opt-in default policy (Germany; *B* = 0.655, SE = 0.119, *P* < 0.001). There is also support for the alternative explanation based on perceived reputation building, as participants under opt-out (Austria) compared with an opt-in (Germany) country perceive lower reputation gains associated with organ donation (*B* = −0.338, SE = 0.146, *P* = 0.021). A parallel mediation analysis supports the proposed mechanism by showing that elevated perceptions of supply sufficiency under opt-out explain reduced willingness to make a directed altruistic living donations (*B* = −0.081, SE = 0.041; *P* < 0.050), but not for nondirected altruistic donation, i.e. stranger (*B* = 0.007, SE = 0.043; *P* = 0.871). For the alternative mechanism, we find that lower perceptions of reputation building partially mediate the effect on altruistic donations (directed: *B* = −0.058, SE = 0.031; *P* = 0.064; nondirected: *B* = −0.061, SE = 0.032; *P* = 0.057). These findings suggest that both perceptions of supply sufficiency and reputation building play a complementary role in explaining the crowding-out effect.

Taken together, study 2 provides correlational evidence that the opt-out policy reduces living organ donations by lowering people's willingness to make altruistic, but not familial, donations. This effect appears to be driven by increased perceptions of supply sufficiency and reduced perceptions of reputation gain under opt-out.

## Study 3: experimental manipulation of default policy and registration rates

Study 3 aims to replicate the findings of Study 2 in a randomized experiment and disentangle whether the crowding-out effect is driven by the policy, registration rates, or both. We also aim to provide further evidence for the proposed mechanism of supply sufficiency and the alternative mechanism of reputation building. We conducted a randomized 2 × 2 between-subjects experiment crossing default policy (opt-in vs. opt-out) with information on deceased organ donation registration rates (15 vs. 85%). This yielded two conditions congruent with situations observed in the real world (i.e. opt-in/15%; opt-out/85%) and two less typical conditions (i.e. opt-in/85%; opt-out/15%). Participants read a scenario describing one of the four situations and then completed the same measures as in study 2. We collected data from 1,721 participants from Austria and Germany with representative demographic profiles (49% female; *M*_age_ = 44.28, SD = 14.85).

We find a significant negative effect of the opt-out policy on the willingness to become a living organ donor for altruistic donations (directed: *B* = −0.200; SE = 0.069; *P* = 0.004; nondirected: *B* = −0.212, SE = 0.074; *P* = 0.004) but not for familial donations (*B* = −0.048, SE = 0.072; *P* = 0.506). The registration rate did not have any direct effects but moderated the effect of the default policy on directed altruistic donations (*B* = −0.280; SE = 0.138; *P* = 0.042), such that the opt-out policy only reduced willingness to make a directed altruistic donation when registration rates are high. This moderating effect implies that policy and registration rates matter. Multiple comparisons further support the joint influence of policy and registration rate, showing that significant differences between opt-in and opt-out policy only occur at a high (directed: *B* = −0.337, SE = 0.097, *P* < 0.001; nondirected: *B* = −0.242, SE = 0.104, *P* = 0.021) but not at a low registration rate (directed: *B* = 0.057, SE = 0.099, *P* = 0.562; nondirected: *B* = −0.181, SE = 0.106, *P* = 0.088). The willingness to make a directed altruistic donation was also significantly lower (*B* = −0.209, SE = 0.097, *P* = 0.031) under an opt-out policy with 85% registration rate (vs. opt-in, 15%). These findings corroborate the results of study 2 and show that the crowding-out effect caused by the opt-out default policy operates mainly through a reduced likelihood of making an altruistic (vs. kinship) donation, and especially when registration rates are high.

Parallel mediation analysis showed a significant indirect effect via perceived supply sufficiency on willingness to make a directed altruistic donation (*B* = −0.027, SE = 0.013; *P* = 0.039), which is mainly driven by the registration rate (*B* = −0.024, SE = 0.012; *P* = 0.039). Thus, supply sufficiency explains the crowding-out effects on directed altruistic donations, with higher registration rates being the driving factor. For reputation building, in contrast, we do not observe such an indirect effect (*B* = 0.001, SE = 0.012; *P* = 0.967). These findings suggest that the perceived supply sufficiency mechanism offers a more robust explanation for why opt-out defaults with high registration rates reduce living donations than reputation building. This is an important insight, as the registration rate often serves as a proxy when communicating the success of the opt-out default policy (9).

In sum, study 3 demonstrates that people's willingness to make an altruistic living organ donation is influenced by both the donation policy and the registration rate, with the strongest crowding-out effects emerging when the two are congruent (opt-out, 85%). The findings also confirm that perceptions of supply sufficiency are primarily driven by the registration rate, potentially serving as a proxy for organ supply. Expanding on this idea, we examine the role of information about actual organ supply in the following study.

## Study 4: experimental manipulation of default policy and organ shortages

Study 4 extends the findings of study 3 by examining the role of organ shortages for donation when policy and registration rates are congruent. We hypothesize that greater perceived shortages will lead to lower perceptions of supply sufficiency and a higher willingness to be a living donor. We also test the alternative mechanism that opt-out leads to a greater injunctive social norm to donate deceased organs and thus a lower need to be a living donor. Injunctive norms refer to what people “ought” to do. There is evidence that injunctive norms increase levels of prosocial behavior (39–42) and that opt-out defaults are associated with enhanced injunctive norms (43). As such, moving to an opt-out default should increase people's injunctive norm that they should be deceased organ donors. This will also be enhanced by the perceived descriptive norm that registration rates are higher under opt-out, so not only is donation the normative behavior, but it is also a behavior that people should do. Indeed, cooperation is higher when descriptive and injunctive norms are operating together (42), and the “social radar” model of norms indicates that descriptive norms, as embodied in the opt-out default, are socially monitored and reinforce the decision to act in accordance with the injunctive norm (44). Thus, the injunctive norm to be a deceased organ donor will be higher under opt-out, and this will drive the normative focus (45) onto deceased organ donation and away from being a living donor.

We conducted a randomized 2 × 2 between-subjects experiment, manipulating policy and congruent registration rates (opt-in with 40% vs. opt-out with 99%) with the presence or absence of information about organ shortages. The scenario and measures were consistent with those used in study 3. We collected data from 1,582 participants from Germany with representative demographic profiles (49% female; *M*_age_ = 45.23, SD = 14.57).

We find a positive main effect of the shortages on the willingness to become a nondirected altruistic living organ donor. When shortages are present, people are more likely to give to strangers (*B* = 0.230, SE = 0.073, *P* = 0.002) while the effects on directed altruistic (*B* = 0.120, SE = 0.072, *P* = 0.099) and familial donations (*B* = 0.114, SE = 0.078, *P* = 0.143) are less pronounced. We also observe a main effect on supply sufficiency: when shortages were present, perceived supply sufficiency was lower (*B* = −0.317, SE = 0.072, *P* < 0.001). Furthermore, as predicted, exposure to an opt-out policy resulted in perceived greater supply sufficiency (*B* = 0.823, SE = 0.070, *P* < 0.001) and a stronger injunctive norm for deceased organ donation (*B* = 0.334, SE = 0.067, *P* < 0.001).

Parallel mediation analysis showed that the main effect of shortages on nondirected altruistic donations was mediated by perceived supply sufficiency (*B* = 0.042, SE = 0.013, *P* = 0.001) but not injunctive norms (*B* = 0.002, SE = 0.005, *P* = 0.657). Thus, highlighting organ shortages increases willingness to be a nondirected altruistic living organ donor by increasing people's perception of insufficient supply to meet demand.

We further expected that mentioning shortages would have stronger effects on perceived supply sufficiency under opt-out compared with opt-in. Supporting this idea, we find a negative interaction between shortages and opt-out on supply sufficiency (*B* = −0.441, SE = 0.143, *P* = 0.002). This finding extends study 3 by showing that supply sufficiency is highest under opt-out with high registration rates and no mention of shortages. It further indicates that highlighting shortages might be a means to reduce supply sufficiency perceptions under opt-out, an idea that we further aim to explore in study 5.

In sum, study 4 shows that highlighting shortages significantly increases willingness to become a living organ donor by reducing perceived supply sufficiency, and this effect is particularly pronounced under an opt-out vs. opt-in default policy.

### Internal meta-mediation analysis of supply sufficiency mechanism

**Fig. 3.**
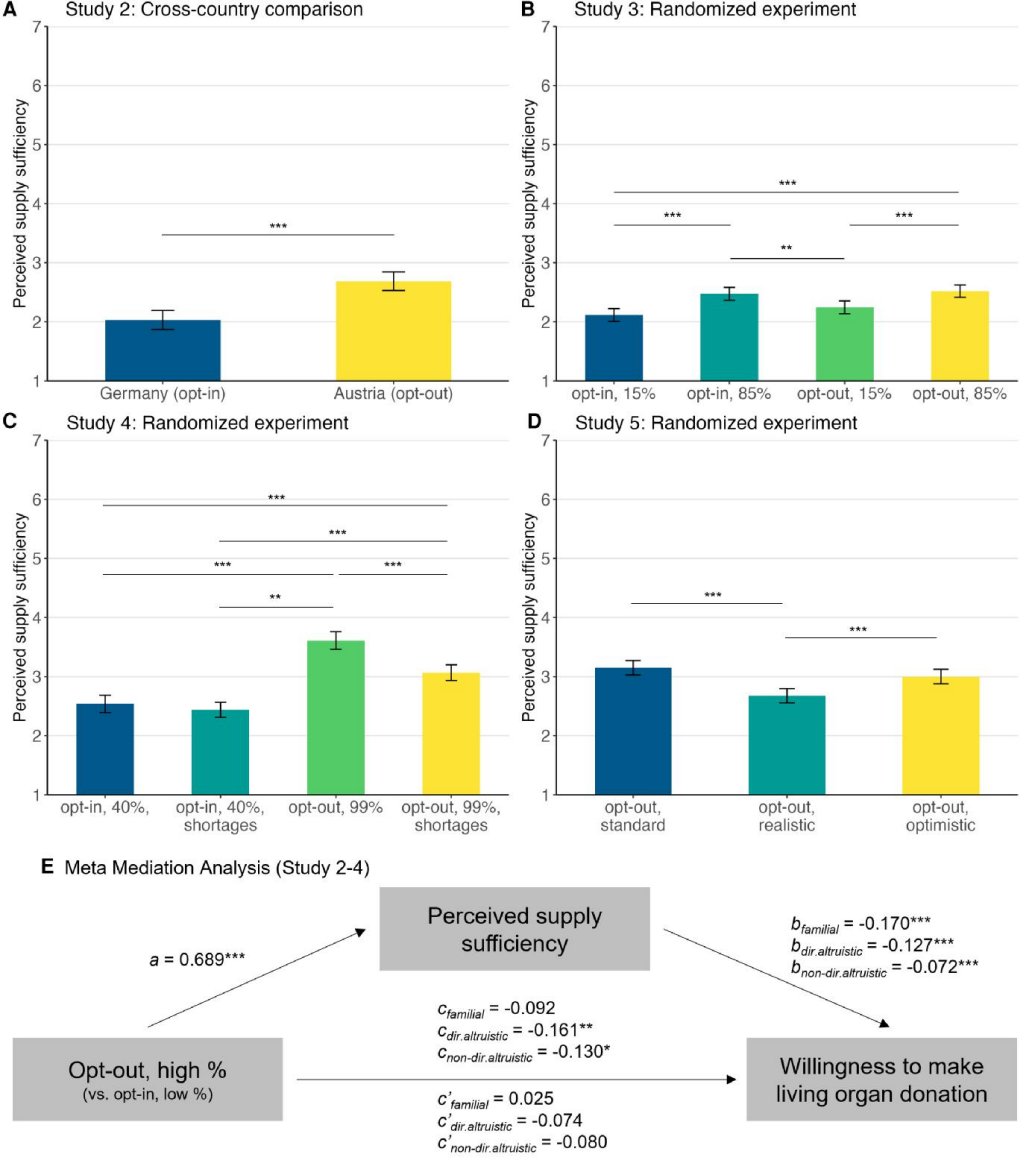
Opt-out policy reduces willingness to make altruistic living organ donation by increasing perceived sufficiency of organ supply (studies 2–5). A cross-country comparison (A) and three randomized experiments (B–D) show that perceived sufficiency of organ supply is higher under opt-out than under opt-in. Plots in A–D show conditional effects on perceived supply sufficiency when all covariates are at their mean. Panel E illustrates the results of an internal meta-mediation analysis using pooled data from Studies 2 to 4. We used a simplified mediation model only comparing congruent policy and registration rates (i.e. opt-out, high% vs. opt-in, low%). Familial = living donation to a core family member (e.g. sibling, spouse); dir.altruistic = directed living donation to a friend, distant relative or acquaintance; non-dir.altruistic = nondirected living donation to a stranger. Error bars represent 95% CIs of these effects. Significance levels of differences: * *P* < 0.05, ** *P* < 0.01, *** *P* < 0.001.

As the results across studies 2–4 show a consistent pattern for the proposed supply sufficiency mechanism, we pooled the data from all three studies to explore a meta-mediation model with more extensive statistical power. To integrate data from all three studies, we test a simplified model only including the two congruent conditions (opt-out, high% vs. opt-in, low%). We find that moving along the congruency line from opt-in (low%) to opt-out (high%) decreases willingness to make an altruistic donation (directed: *B* = −0.160; SE = 0.056; *P* = 0.004; nondirected: *B* = −0.130, SE = 0.058; *P* = 0.024) while we find no significant effect on familial donation decisions (*B* = −0.090, SE = 0.060; *P* = 0.132). In support of our proposed mechanism, we find that perceptions of supply sufficiency mediate both effects of altruistic donation (directed: *B* = −0.091; SE = 0.016; *P* < 0.001; nondirected: *B* = −0.052, SE = 0.016; *P* = 0.001). Figure 3 illustrates the findings of this internal meta-mediation analysis. In sum, these findings further support the proposed mechanism based on supply sufficiency and rule out reputation concerns and injunctive social norms as alternative explanations.

## Study 5: experimental manipulation of default policy and organ shortages

Study 5 explores whether providing a more realistic preview of the effectiveness of the opt-out default policy attenuates the crowding-out effect on living organ donations and further explores the relative contributions of the supply sufficiency and injunctive norms mechanisms. We conducted a randomized one-way (at three levels) experiment: (i) a standard preview condition in which we did not add any further information; (ii) a realistic preview condition based on the insights from the epidemiological study highlighting that an opt-out policy will not solve the organ shortage problem; and (iii) an optimistic preview that aligns with policymaker's usual advocacy for the opt-out default. We hypothesized that supply sufficiency will be lower under the realistic opt-out version than both the opt-out and optimistic opt-out conditions, indirectly increasing people's willingness to make a living altruistic donation.

To enhance realism in this study, we took advantage of an upcoming policy change to an opt-out default in Switzerland in 2026. We ran the study a year ahead of the planned policy change. We briefed participants about the current opt-in policy, including information on current registration rates (23%) and organ shortages (2–3 years waiting time for a kidney). Participants were asked to imagine that the opt-out policy was already in place and to respond to the same dependent variables and covariate as in the previous studies. We also asked participants whether they voted for or against the implementation of the opt-out policy, as this was decided in a public vote in May 2022. We collected data from 1,225 participants from Switzerland with representative demographic profiles (49% female; *M*_age_ = 43.86, SD = 14.47).

Consistent with our predictions, the realistic opt-out preview is associated with significantly lower expectations of registration rates for a deceased donation (*B* = −8.553, SE = 1.418, *P* < 0.001), reduced policy effectiveness (*B* = −0.701, SE = 0.088, *P* < 0.001), and reduced perceived supply sufficiency (*B* = −0.401, SE = 0.075, *P* < 0.001) compared with the standard and optimistic conditions. Injunctive social norms were not significantly different across conditions (*B* = 0.020, SE = 0.077, *P* = 0.795). Notably, the standard and optimistic preview opt-out conditions did not differ significantly in terms of registration rates (*B* = −1.541, SE = 1.562, *P* = 0.324), policy effectiveness (*B* = −0.093, SE = 0.090, *P* = 0.304), supply sufficiency (*B* = −0.149, SE = 0.091, *P* = 0.101), and injunctive social norms (*B* = −0.083, SE = 0.092, *P* = 0.364). This finding shows that people tend to have an overly optimistic view of the effectiveness of the opt-out policy, but this belief can be corrected with a more realistic preview.

Parallel mediation analysis shows a significant indirect effect of realistic preview (vs. standard and optimistic) on willingness to make an altruistic donation via perceived supply sufficiency (directed: *B* = 0.075, SE = 0.019, *P* < 0.001; nondirected: *B* = 0.061, SE = 0.017, *P* < 0.001). Thus, the realistic preview reduces perceived supply sufficiency compared with the baseline and optimistic opt-out conditions, thereby indirectly increasing willingness to be a living organ donor. There were no indirect effects via injunctive norms (directed: *B* = 0.002, SE = 0.009, *P* = 0.795; nondirected: *B* = 0.002, SE = 0.008, *P* = 0.796).

In sum, study 5 shows that when we focus just on opt-out (with no comparison with opt-in) but vary the information on its effectiveness (baseline, optimistic, and realistic), we see again that supply sufficiency is the key mechanism that is sensitive to the effectiveness of opt-out. Also, the findings show that, all things being equal, people perceive the opt-out policy in an overly optimistic way.

## Discussion

Our findings show that opt-out default policies effectively nudge individuals toward more socially desirable behaviors but can also have unintended negative consequences. We provide evidence that opt-out defaults can have a crowding-out effect on related behaviors not targeted by the intervention, substantially diminishing the overall intended welfare gains. Specifically, we show that across countries that adopted an opt-out policy for deceased organ donation over the past 20–25 years, there was only a marginal average increase in deceased donors (+1.21 pmp, +7%, not significant), but a significant decrease in average annual living donors (−4.59 pmp, −29%). We find consistent evidence across our studies that this crowding-out effect on living organ donors is driven by a reduction in altruistic (vs. kinship) donations and is attributable, in part, to people being more likely to perceive that supply sufficiency meets that demand under an opt-out. Other potential mechanisms, based on reputation building and injunctive norms, also played a role but were not as central for explaining the crowding-out effect as the proposed supply sufficiency mechanism (Table 2).

**Table 2.** Summary of key findings.

Study	Groups	Deceased donation	Living donation			Proposed mechanism (and alternatives)		
Behavioral comparison between opt-in and opt-out								
Study 1	Opt-in^a^	16.70 pmp	15.63 pmp					
	Switch to opt-out	+1.21 pmp (1.245)	−4.59* pmp (2.228)					
			Directed familial	Directed altruistic	Nondirected altruistic	Supply sufficiency	(Reputation building)	(Injunctive social norm)
Mechanisms between opt-in and opt-out								
Study 2	Opt-in, 36%		5.796*** (0.102)	4.098*** (0.107)	2.706*** (0.111)	2.032*** (0.082)	3.327*** (0.104)	
	Opt-out, 99%		0.038 (0.140)	−0.418** (0.151)	−0.347* (0.157)	0.655*** (0.116)	−0.338* (0.147)	
Study 3	Opt-in, 15%		5.690*** (0.073)	3.876*** (0.070)	2.588*** (0.075)	2.115*** (0.056)	3.074*** (0.070)	
	Opt-in, 85%		0.089 (0.103)	0.070 (0.099)	−0.009 (0.106)	0.359*** (0.079)	0.110 (0.099)	
	Opt-out, 15%		0.023 (0.103)	−0.057 (0.099)	−0.181+ (0.106)	0.131+ (0.079)	0.022 (0.099)	
	Opt-out, 85%		−0.027 (0.101)	−0.267** (0.097)	−0.251* (0.104)	0.405*** (0.077)	−0.004 (0.098)	
Study 4	Opt-in, 40%, shortages		5.851*** (0.072)	3.825*** (0.067)	2.392*** (0.067)	2.438*** (0.065)		4.243*** (0.063)
	Opt-in, 40%		−0.119 (0.109)	−0.159 (0.102)	−0.253* (0.102)	0.101 (0.099)		−0.134 (0.095)
	Opt-out, 99%, shortages		−0.152 (0.104)	−0.108 (0.096)	−0.058 (0.097)	0.630*** (0.094)		0.243** (0.090)
	Opt-out, 99%		−0.260* (0.110)	−0.186+ (0.103)	−0.265* (0.104)	1.172*** (0.100)		0.318*** (0.096)
Internal meta-analysis (S2–S4)	Opt-in, low%		5.723***	3.749***	2.362***	2.640***		
	Opt-out, high%		−0.092 (0.059)	−0.161** (0.055)	−0.130* (0.057)	0.689*** (0.050)		
Mechanisms within opt-out								
Study 5	Opt-out, standard		5.598*** (0.072)	3.743*** (0.067)	2.305*** (0.070)	3.151*** (0.062)		4.154*** (0.064)
	Opt-out, realistic		0.043 (0.102)	0.065 (0.094)	−0.018 (0.098)	−0.475*** (0.087)		−0.021 (0.090)
	Opt-out, optimistic		0.046 (0.103)	−0.025 (0.095)	−0.057 (0.099)	−0.149+ (0.088)		−0.083 (0.090)

The table presents key group comparisons for each study. The first group (in gray font) serves as the baseline for the comparison and shows the group mean. All other estimates (in black font) indicate differences from this baseline with covariates held at their mean. Standard errors are in parentheses. Living donation types are: Directed familial = to a core family member; Directed altruistic = to a friend, distant relative, or acquaintance; nondirected altruistic = to a stranger. pmp = donors per million population.

^a^Average donor rates per million population (pmp) in switching countries prior to the opt-out adoption. Significance levels: +*P* < 0.1, *P < 0.05, **P < 0.01, ***P < 0.001.

This shift in the perception of supply sufficiency has implications for many key endeavors where individual cooperation at a societal level is necessary to achieve the public good. For example, while opt-out defaults have been reported to increase uptake of influenza vaccinations (46, 47), our findings suggest they may also decrease attention to nontargeted but related risk-mitigation behaviors, such as social distancing or hygiene practices. Similarly, while opt-out defaults have been shown to increase domestic green energy use (48), they may also reduce engagement in other pro-environmental behaviors, such as recycling or reduced air travel. While such crowding-out effects have been discussed as a within-person phenomenon—arising from individuals' own adoption of a sustainable behavior (49)—our research suggests that behavioral interventions, like opt-out defaults, might also trigger them. This highlights the importance of looking beyond direct effects on the target behavior to fully understand the broader welfare implications of such policies.

Our findings, therefore, tie in with recently emerging research on the unintended consequences of default nudges, showing that the effects of automatic enrollment in retirement saving plans can lead to increases in household debt (29) and that asking people about their support for, or exposing them to, an opt-out default policy for green energy usage can reduce their support for related system-level policies (28, 50). We add to these emerging studies by providing evidence of crowding-out effects in the context of high-cost prosocial behavior.

Our work also complements prior work on organ donation defaults, which has reported differences in rates of living and deceased organ donors between countries with opt-in and opt-out default policies (13, 14, 51). We extend these findings by providing quasi-experimental evidence on how transitioning from an opt-in to an opt-out policy impacts donor rates. Moreover, we provide insights into a boundary condition of the crowding-out effect, such that default nudges only affect altruistic donations (e.g. to strangers, acquaintances) but not kinship-based donations (i.e. family members), and identify an underlying mechanism based on the perception of the supply sufficiency of a public good.

From a theoretical perspective, our findings also complement existing models of human cooperation by offering more insights into how high-cost cooperative decision-making depends on the availability of a low-cost resource and recipient kinship (23). We extend these models by showing that perceived uptake in the availability of a low-cost resource (i.e. deceased donor organs) driven by a change in the opt-out default can crowd out more costly cooperative behaviors (i.e. becoming a living organ donor), but this only applies for altruistic not kinship-based cooperative behavior. Interestingly, although helping a distant relative offers inclusive fitness benefits over helping a stranger (21, 52), these benefits do not ensure cooperation when the perceived supply for a high-cost cooperative act can be met through another means. This provides new insights into human cooperation and is essential when policies or campaigns are designed to alter the availability of a public good.

Our study also reveals a dilemma for policymakers wishing to promote organ donation by introducing an opt-out default policy, as the small increase in deceased donors is roughly offset by a substantial decrease in living donors. This has potential health consequences as a kidney from a living donor (compared with a deceased donor) results in significantly higher quality of life (34) and patient survival rates (35, 36). So, for any policy switch to an opt-out default, strategies are needed to mitigate the loss of living donors (27).

One-way forward might be to be more open about the effectiveness of opt-out to meet the demand for organs and, based on actual evidence, communicate that a 10–20% increase in organs can be expected, which is clinically significant, but not to see opt-out as a panacea to the supply problem. Also, it should be made clear that living organ donors are still needed—a major communication task for policymakers. By identifying perceived supply sufficiency as the key underlying mechanism for the crowding-out effects, we provide a theoretical basis for policymakers to develop further communication strategies to contribute to the promotion of altruistic donations under opt-out policies.

There are limitations to our studies. The experimental studies were conducted in the Global North, and generalizability to wider cultural groups and ethnicities is limited (53). However, the change to an opt-out organ donation policy, compared with an opt-in policy, results in a reduction in living organ donors observed across the world. Thus, the behavioral phenomena do seem to generalize, and as such, the proposed supply-demand mechanism should also generalize. While our experimental studies focused on the reported willingness to donate, the pattern of results observed for willingness mirrored actual behavioral data; as such, we have confidence that the willingness data reflects behavior. We are not aware of any global database that provides disaggregated data on altruistic vs. kinship donations, but including this distinction in the reporting of organ statistics would help future research to gain more nuanced insights into behavior.

## Materials and methods

### Ethics and preregistration

This research complies with the Declaration of Helsinki (2023) for human subject research. Study 1 used fully anonymized, publicly available aggregate data and did not require ethical approval. Studies 2 and 3 were approved by the Research Ethics and Data Management Committee of the Tilburg School of Humanities and Digital Sciences (REDC #2019/110, 2019 November 19). Studies 4 and 5 received approval from the Ethics and Data Management Committee of the University of Hamburg Business School (2024 December 6). We preregistered the hypotheses and methods for studies 2 and 3 (https://osf.io/s4zv6), study 4 (https://osf.io/tych7), and study 5 (https://osf.io/eb84s). Deviations from the preregistration are detailed in Tables S7.1 and S7.2.

### Methods—study 1

#### Sampling procedure and data sources

The basis for our sampling was countries that contributed national organ donor statistics to IRODaT, a publicly accessible database reviewed by a network of experts (30). Additional data for each of the four UK countries was sourced from annual NHSBT reports (31). We collected data on actual deceased and living organ donor rates per million population. The period of investigation was determined by data availability considerations, as there was only limited data before 2000 and many countries did not yet submit data after 2023 at the time of data collection. We excluded all countries that (i) already relied on opt-out in the first year of study period or before, (ii) switched to opt-out in the last year of study period, (iii) changed their default consent legislation from opt-in to opt-out and back during the study period, (iv) introduced opt-out only for special cases, e.g. for victims of violent death or certain parts of the country, (v) had <1 million inhabitants, (vi) had >50% missing data for living or deceased donor rates, and (vii) had missing data in the final 5 years of the study period. In sum, 24 countries met all inclusion criteria and could be used for data analysis (Fig. S1.1 and Table S1.1).

In the final sample, some data points were missing due to gaps in reporting (Fig. S1.2). To address this issue, we used the multiple imputation approach implemented in Amelia II (54). This algorithm specifically accounts for the panel structure of the data and jointly imputes missing values using leads and lags of both log(living donations pmp) and log(deceased donations pmp), as well as log(road traffic accident rates) and a squared time trend. Logarithmic transformations were applied solely during imputation to satisfy the assumption of multivariate normality and were reverted to level values for subsequent difference-in-differences estimation. Furthermore, observations are assumed to be missing at random, meaning that the probability of a data point being missing solely depends on the observed data.

#### Analytical approach

To quantify the effect of adopting an opt-out policy, we applied the difference-in-differences estimator developed by Callaway and Sant’Anna, which accommodates staggered treatment adoption (19). Their estimator isolates the treatment effect of opt-in adoption by comparing variations in adopting countries around the time of adoption with analogous countries that retain an opt-in policy. This approach identifies the treatment effect by comparing temporal changes in countries that adopted opt-out policies with contemporaneous changes in countries that retained opt-in policies. It explicitly accounts for variation in adoption timing and allows treatment effects to differ across both time and cohorts. As with standard difference-in-differences models, identification relies on the assumption that, in the absence of policy change, the difference in donation rates between treated and untreated countries would have remained constant (the “parallel trends” assumption). We adopted a relaxed version of this assumption, requiring parallel trends conditional on covariates. We further allowed for anticipation effects of up to 3 years, reflecting the typical lag between policy announcement and implementation. During this period, the parallel trends assumption is also assumed to hold absent the policy announcement. Standard errors are adjusted for multiple imputations, as outlined by Honaker et al. (54).

All analyses were conducted in R (v4.3.0) (55) using the *Amelia II* package (v1.8.1) for imputation (54) and the *did* package (V2.1.2) for estimating the difference-in-differences models (19). The raw time series can be found in Supplementary File S1.

#### Robustness checks

We conducted three types of robustness checks to assess the stability of the results. First, we re-ran the model without imputed data. Second, we used a more conservative sample selection, by excluding countries that have further switched to a “hard” opt-out policy after the initial soft opt-out adoption, in which the possibility of family objections limited (i.e. Slovak Republic in 2017 and Argentina in 2018). Third, we also estimated a model in which we exclude the COVID years (2020–2023) for each of the previous sampling approaches to make sure that these years did not bias our results in any way. All checks confirmed the directional pattern of results from the main study (see Supplementary File S1, Robustness Checks).

### Methods—study 2

#### Sampling and participants

We conducted an online survey using Unipark (www.unipark.com) and recruited participants through a professional panel provider (https://respondi.com). Our preregistered target was 200 respondents per country; the final sample comprised 435 adults from Germany (*n* = 210) and Austria (*n* = 225), with demographic quotas representative of age and gender (*N*_GER_ = 210; *N*_AT_ = 225; 49% female; *M*_age_ = 44.17; SD = 15.06). Participants were compensated via a point-based reward system and provided informed consent.

#### Study design

Participants first received country-specific information on organ donation defaults and registration rates (Table S2.1). German participants were informed of their country's opt-in system and the 2019 registration rate of ∼36% (56), while Austrian participants learned about their opt-out system and the ∼99.5% presumed consent rate (57). Participants then received information on the procedure and consequences of making a living organ donation.

#### Dependent variables

Participants read a scenario in which someone requires a kidney or liver lobe transplant and were asked to rate their willingness to donate to various recipients (close family, distant relatives, friends, acquaintances, and strangers) on 7-point Likert scales. To prevent order effects, the display of the different recipient types was randomized across participants. Responses were averaged across organs to form composite scores for each recipient type (Table S2.1).

#### Mediators and covariates

Subsequent items assessed perceptions of organ supply sufficiency (e.g. “I think that the need for organs is already sufficiently covered by the number of existing donors” (37)) and prosocial reputation motives (e.g. “Donating organs gives me social approval” (38)) on 7-point Likert scales, with randomization of item order. Participants then completed covariate items, demographic questions, and a debrief (Table S2.1). Descriptive statistics and correlations are in Table S2.2.

#### Analytical approach

We employed a path modeling approach to estimate all relationships simultaneously (58) and estimated the models using M*Plus* v8.9 with a robust maximum likelihood estimator (59). We used a factor analysis to ensure that all measures meet reliability and validity criteria (Table S2.1). Full results are presented in Tables S2.3–S2.5, with visualizations in Figs. S2.1 and S2.2.

### Study 3—methods

#### Sampling and participants

We conducted an online experiment using Unipark (www.unipark.com) and recruited participants via a professional panel provider (https://respondi.com) in Germany and Austria. Our preregistered target was 200 participants per condition in each country. Slight oversampling by the panel provider yielded a total of 1,721 adults with demographic quotas representative of age and gender (49% female; *M*_age_ = 44.28, SD = 14.85). Participants received compensation via a redeemable points system and provided informed consent.

#### Study design

We employed a 2 × 2 between-subjects design, manipulating the default policy (opt-in vs. opt-out) and the proportion of registered deceased organ donors (15% vs. 85%) as a proxy for organ supply. Participants were asked to imagine living in a country characterized by one of the resulting four conditions (Table S3.1) and then received information on the procedure and consequences of making a living organ donation. Group-level descriptive statistics are presented in Table S3.2.

#### Dependent variables

Participants rated their willingness to make a living donation of a kidney or liver lobe to five recipient types (close family, distant relatives, close friend, acquaintance, and stranger), using the same measures as in study 2. Ratings for both organs were averaged to form composite scores per recipient category.

#### Mediators and covariates

We included the same measures for the mediators—perceptions of supply sufficiency and reputation gains—along with the same covariates as in study 2 (Table S3.1). We added country (0 = Germany; 1 = Austria) as an additional covariate in the model to adjust for national differences.

#### Analytical approach

We verified reliability and validity of all multi-item measures via factor analysis; all scales met standard criteria (Table S3.1). We used path modeling to estimate relationships simultaneously (58), with robust maximum likelihood estimation implemented in M*Plus* v8.9 (59). Complete model results are provided in Tables S3.3–S3.5, with visual summaries in Figs. S3.1 and S3.2.

### Study 4—methods

#### Sampling and participants

We conducted an online survey using Qualtrics (www.qualtrics.com) and recruited participants residing in Germany through a professional panel provider (www.bilendi.de). Participants were compensated for their time. The preregistered sample size was 400 participants per condition (*n* = 1,600). The final sample comprised 1,582 adults with demographic characteristics representative of the German population (49% female; *M*_age_ = 45.23, SD = 14.57). All participants provided informed consent.

#### Study design

We conducted a randomized experiment employing a 2 (default with congruent registration rates: opt-in with 40% vs. opt-out with 99%) × 2 (shortages: present vs. absent) between-subjects design. The manipulations were embedded in an immersive scenario (Table S4.1), based on recent registration data from Germany and Austria to enhance realism.

#### Dependent variables

We used the same measures as in studies 2 and 3 to capture participants' willingness to make a living kidney donation for a close family member, a remote relative, a close friend, an acquaintance, and a stranger. We counterbalanced the order of appearance of these questions.

#### Mediators and covariates

Participants next rated their perceptions of supply sufficiency, as in studies 2 and 3. We further measured injunctive social norm toward deceased organ donation as an alternative mechanism (e.g. “I would feel that I should also be an organ donor after my death” (60)) on a 7-point Likert scale. All measures and descriptive statistics are reported in Tables S4.1 and S4.2.

#### Analytical approach

All multi-item measures were found to be reliable and valid (Table S4.1). We used path modeling with robust maximum likelihood estimation in M*Plus* v8.9 (59), including all preregistered covariates. Full model results are provided in Tables S4.3 and S4.4, and visualizations in Figs. S4.1 and S4.2. The internal meta-mediation analysis pooling data from studies 2 to 4 is presented in Table S6.1.

#### Power calculations

Based on effect sizes from study 3 (Cohen's *d* ≈ 0.13), a power analysis for a 2 × 2 between-subjects design (*α* = 0.05, power = 0.80) indicated a required sample of 1,600 participants (400 per condition).

### Study 5—methods

#### Sampling and participants

We conducted an online survey using Qualtrics (www.qualtrics.com) and recruited participants residing in Switzerland via a professional panel provider (www.bilendi.de). Participants received monetary compensation. Our preregistered sample size was 400 participants per condition (*n* = 1,200). The final sample included 1,225 adults with representative demographics (49% female; *M*_age_ = 43.86, SD = 14.47). All participants provided informed consent.

#### Study design

We conducted a randomized between-subjects experiment manipulating the communicated effectiveness of the opt-out default policy at three levels: (i) *baseline*—no information provided; (ii) *realistic preview*—highlighting limited policy effectiveness based on our epidemiological findings; and (iii) *optimistic preview*—emphasizing opt-out as the most effective policy to address organ shortages, reflecting typical policymaker framing (Table S5.1).

#### Dependent variables

As in studies 2–4, we measured willingness to donate a kidney to five recipient types (close family, distant relative, close friend, acquaintance, and stranger). We counterbalanced the order of questions.

#### Mediators and covariates

Participants then completed measures assessing perceptions of supply sufficiency and injunctive social norms toward deceased donation, using the same items as in study 4. Descriptive statistics by condition are presented in Table S5.2.

#### Analytical approach

All multi-item scales demonstrated acceptable reliability and validity (Table S5.1). We estimated all relationships simultaneously using path modeling with robust maximum likelihood estimation in MPlus v8.9 (59), including all preregistered covariates. Detailed results are reported in Table S5.3, with visualizations in Figs. S5.1–S5.3.

#### Power calculations

Based on prior effect sizes (Cohen’s *d* ≈ 0.13 from studies 2 and 3), we conducted a power analysis for a one-factorial between-subjects design with three groups. To achieve 80% power (*α* = 0.05), the required sample was 1,200 participants (400 per group).

## Supplementary Material

pgaf311_Supplementary_Data

## Data Availability

All study materials, data, and analysis codes are publicly available (https://osf.io/5kbha/).

## Preprints

This manuscript was posted on a preprint: https://osf.io/preprints/psyarxiv/c36v8_v2.
