# Supplementary material for: Crowding-out effects of opt-out defaults: Evidence from organ donation policies
Source: PNAS Nexus. 2025 Oct 28;4(10):pgaf311. doi: 10.1093/pnasnexus/pgaf311 (PMC12560092; doi:10.1093/pnasnexus/pgaf311)
Supplement: pgaf311_Supplementary_Data [file pgaf311_supplementary_data.pdf]

Crowding out Effects of Opt-out Defaults: Evidence from Organ Donation Policies

Supplementary Files

List of Contents

Supplementary File 1: Sampling and Supporting Analysis for Study 1 4

Main analysis. 4

Figure S1.1: Sampling strategy and criteria. 4

Table S1.1: Overview of included countries, switching dates, and legal frameworks. 5

Figure S1.2: Panel data for deceased and living organ donors. 8

Figure S1.3: Model free time series plots for deceased and living donor rates. 9

Robustness checks. 10

Table S1.2: Robustness checks: Overview of model specifications. 11

Table S1.3: Robustness checks: Overview of key findings across all robustness checks. 11

Table S1.4: Robustness check model (2): Difference-in-differences model results (no imputation, balanced sampling, 2000-2023). 12

Figure S1.4: Robustness check model (2): Difference-in-differences model results (no imputation, balanced sampling, 2000-2023). 13

Table S1.5: Robustness check model (3): Difference-in-differences model results (conservative sampling, 2000-2023). 14

Figure S1.5: Robustness check model (3): Difference-in-differences model results (conservative sampling, 2000-2023). 15

Table S1.6: Robustness check model (4): Difference-in-differences model results (balanced sampling, 2000-2019). 16

Figure S1.6: Robustness check model (4): Difference-in-differences model results (balanced sampling, 2000-2019). 17

Supplementary File 2: Materials and Supporting Analyses for Study 2 18

Table S2.1: Survey design and measures. 18

Table S2.2: Correlations and means. 21

Table S2.3: Effect of opt-out default policy on willingness to donate a living organ. 22

Table S2.4: Effects of opt-out default policy on willingness to donate a living organ for different types of recipients. 23

Figure S2.1: Differences in willingness to become a living organ donor between Germany (opt-in, 36% registered) and Austria (opt-out, 99.5% registered). 24

Figure S2.2: Differences in perceptions of sufficiency of organ supply and reputational gain between Germany (opt-in, 36% registered) and Austria (opt-out, 99.5% registered) 25

Table S2.5: Parallel mediation analysis for Study 2. 26

Supplementary File 3: Materials and Supporting Analyses for Study 3 27

Table S3.1: Survey design, experimental manipulations, and measures. 27

Table S3.2: Descriptive statistics for each experimental group (n = 1,721). 30

Table S3.3: Main and interaction effects of default policy and registration rate on willingness to make a (i) familial, (ii) directed altruistic, and (iii) non-directed altruistic living organ donation. 31

Table S3.4: Multiple comparisons across experimental groups for (i) familial, (ii) directed altruistic, and (iii) non-directed altruistic living organ donation. 32

Figure S3.1: Differences in willingness to become a living organ donor depending on the policy (opt-in, opt-out) and registration rate (15%, 85%). 33

Figure S3.2: Differences in perceived supply sufficiency and reputational building depending on the policy (opt-in, opt-out) and registration rate (15%, 85%). 34

Table S3.5: Parallel mediation analysis for Study 3. 35

Supplementary File 4: Materials and Supporting Analyses for Study 4 36

Table S4.1: Survey design, experimental manipulations, and measures. 36

Table S4.2: Descriptive statistics for each experimental group (n = 1,582). 39

Table S4.3: Main and interaction effects of congruent policy/registration rate and the presence of organ shortages on willingness to make a (i) familial, (ii) directed altruistic, and (iii) non-directed altruistic living organ donation. 40

Figure S4.1: Differences in willingness to become a familial and altruistic living organ donor depending on the policy and registration rate (opt-in with low%, opt-out with high %) and organ shortages (absent, present). 41

Figure S4.2: Differences in perceived supply sufficiency and injunctive social norms depending on the policy and registration rate (opt-in, 40% vs. opt-out, 99%) and mentioning of organ shortages (absent vs. present). 42

Table S4.4: Parallel mediation analysis for Study 4. 43

Supplementary File 5: Materials and Supporting Analyses for Study 5 44

Table S5.1: Survey design, experimental manipulations, and measures. 44

Table S5.2: Descriptive statistics for each experimental group (n = 1,225). 47

Figure S5.1: Differences in expected registration rate and policy effectiveness depending on description of policy effectiveness. 48

Figure S5.2: Differences in willingness to become a familial and altruistic organ donor depending on description of policy effectiveness. 49

Figure S5.3: Differences in perceived supply sufficiency and injunctive social norms depending on description of policy effectiveness. 50

Table S5.3: Parallel mediation analysis for Study 5. 51

Supplementary File 6: Internal Meta-Mediation-Analysis for Studies 2-4 52

Table S6.1: Parallel mediation analysis – pooled data from Study 2-4. 52

Supplementary File 7: Transparency Notes – Deviations from Pre-registration **53**
Table S7.1: Deviations from pre-registration for Study 2 and 3. 53
Table S7.2: Deviations from pre-registration for Study 4. 55
References **56**

Supplementary File 1: Sampling and Supporting Analysis for Study 1

Main analysis.

Figure S1.1: *Sampling strategy and criteria.*

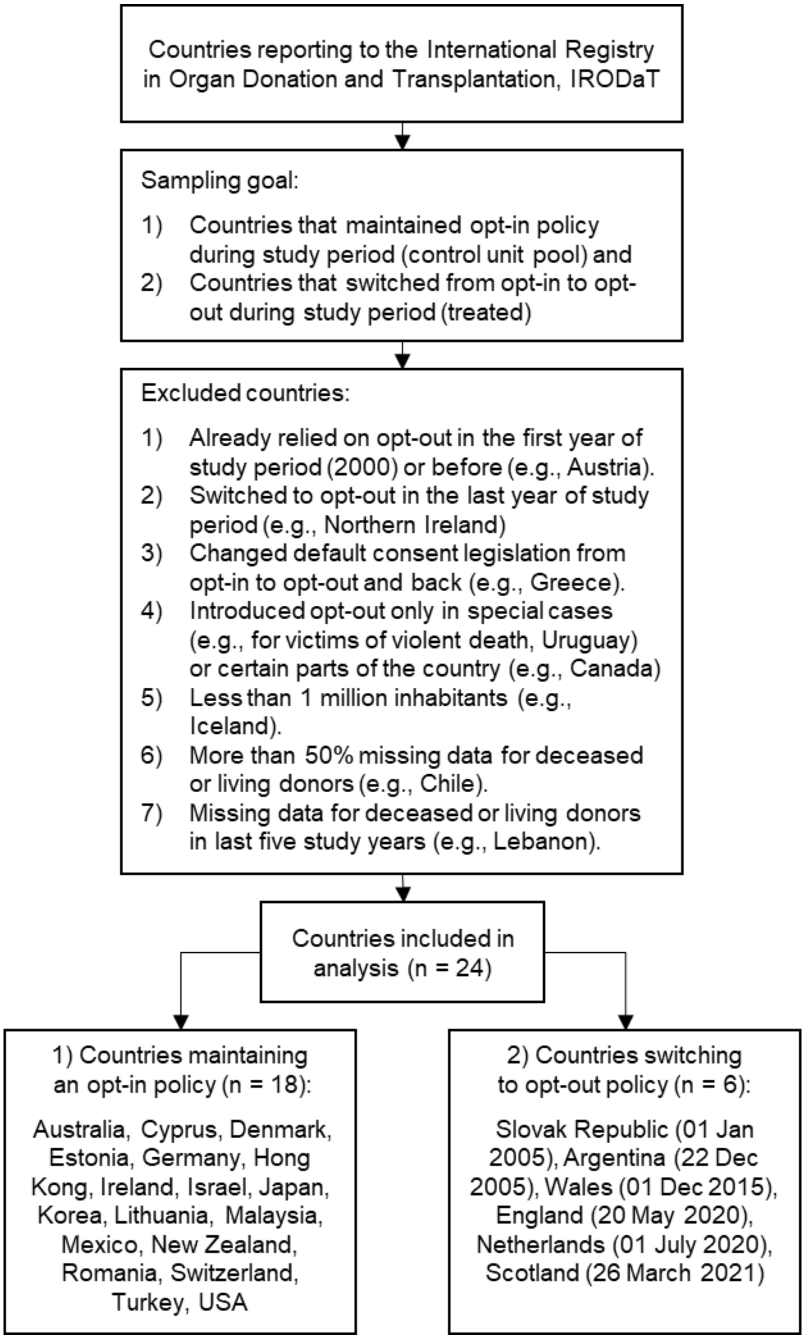

Notes. The diagram shows the sampling goals and exclusion criteria used to determine the final data sample for our main analysis. We disaggregated the UK countries to study their staggered policy adoption in isolation. For UK countries organ donor data was retrieved from annual reports of the National Health Services Blood and Transplant (NHSBT).

Table S1.1: *Overview of included countries, switching dates, and legal frameworks.*

Country	Policy in 2023	Switch to opt-out since 2000	Current Practices Description (as of 2023)	Legal Framework
Argentina*	Opt-out	22 Dec 2005 to soft opt-out<br><br>04 August 2018 to hard opt-out	In 2005, Argentina switched from opt-in to a soft opt-out policy where all adults are presumed to have consented to organ donation unless they have registered an objection. In 2018, they switched to a hard opt-out policy which limits the possibilities for family objections.	Law No. 26,066 on Organ and Tissue Transplants (2005)<br><br>Law No. 27,447 on Organ and Tissue Transplants (2018)
Australia	Opt-in	No	Australia has an opt-in policy where consent is needed before donation can happen. People can register to be a donor on the Australian Organ Donor Register. Families will be asked to agree to donation when their family member dies.	Australian Organ and Tissue Donation and Transplantation Authority Act 2008
Cyprus	Opt-in	No	Cyprus operates under an opt-in system. If a deceased individual hasn't registered their consent, the decision to donate organs rests with their family.	The Human Organ Transplantation Law (N. 127(I)/2007), amended by 102(I)2014 and 160(I)/2017
Denmark	Opt-in	No	Denmark has an opt-in policy requiring individuals to register their consent for organ donation. Family consent is typically sought.	Act No. 402 of 13 June 1990 on the Removal, Storage and Transplantation of Human Organs
England	Opt-out	20 May 2020 to soft opt-out	In 2020, England switched from opt-in to opt-out. Unless individuals opt out, they are presumed to have consented. However, families are still consulted and can object.	Organ Donation (Deemed Consent) Act 2019
Estonia	Opt-in	No	Estonia uses an opt-in organ donation system. Families are consulted even if the person is a registered donor.	Handling and Transplantation of Cells, Tissues and Organs (Act RT I 2002, 21, 118) amended by (RT I 2008, 25, 163)
Germany	Opt-in	No	Germany has an opt-in policy. Individuals must explicitly consent to donate. Families are consulted even if the person is a registered donor.	Transplantation Act as amended by the notice of 4 September 2007 (BGBl. I p. 2206), which was last amended by Article 15 (3) of the Law of 4 May 2021 (BGBl. I p. 882

Country	Policy in 2023	Switch to opt-out since 2000	Current Practices Description (as of 2023)	Legal Framework
Hong Kong	Opt-in	No	Hong Kong operates an opt-in system. Individuals must register their consent on the Centralised Organ Donation Register. Family consent is required.	Human Organ Transplant Ordinance (Cap. 465)
Ireland	Opt-in	No	As of 2023, Ireland uses an opt-in system. People must register their consent, and families must also agree.	Human Tissue Act 2004, with amendments S.I. No. 325 of 2012 and S.I. No. 198 of 2014
Israel	Opt-in	No	Israel uses an opt-in system. Registered donors and their families are given priority in transplant allocation. Family consent is required.	Organ Transplantation Law, 5768–2008
Japan	Opt-in	No	Japan uses an opt-in system. Written consent is required from the donor, and families must approve. Families also decide about donor status if a deceased person's intention is unclear.	Organ Transplant Law (1997, amended 2010)
Korea	Opt-in	No	South Korea uses an opt-in system. Individuals can register as donors, and family consent is also required.	Organ Transplant Act
Lithuania	Opt-in	No	Lithuania follows an opt-in model where explicit consent is needed. Family members can decide if the deceased did not express their will.	Law on Donation and Transplantation of Human Tissues, Cells and Organs (1996, No I-1626), last amended 2016 (No XII-2344)
Malaysia	Opt-in	No	Malaysia has an opt-in system. Consent can be obtained either from the deceased person's expressed will and/or from the next of kin.	National Organ, Tissue and Cell and Transplantation Policy 2007 (MOH/P/PAK/131.07 (BP))
Mexico	Opt-in	No	Mexico operates under an opt-in system. People must consent during their lifetime, and families are consulted. If no consent exists, the next-of-kin must grant permission before retrieval.	General Health Law (Ley General de Salud), Title XIV
Netherlands	Opt-out	01 July 2020 to soft opt-out	The Netherlands has an opt-out system since 2020. All adults are presumed donors unless they opt out. However, families are still consulted and can object to a donation.	Organ Donation Act (Wet op de orgaandonatie)

Country	Policy in 2023	Switch to opt-out since 2000	Current Practices Description (as of 2023)	Legal Framework
New Zealand	Opt-in	No	New Zealand has an opt-in system. Donor status is indicated on driver licenses, and families are consulted.	Human Tissue Act 2008 and Organ Donors and Related Matters Act 2019
Romania	Opt-in	No	In Romania, organ donation follows an opt-in system. If a deceased individual hasn't registered their consent, the decision to donate organs rests with their family.	Law No. 95/2006 on Healthcare Reform, supplemented by LAW No. 9 of 18 January 2016
Scotland	Opt-out	26 March 2021 to soft opt-out	Scotland uses a soft opt-out system introduced in 2021. Consent is presumed unless opted out, with families still consulted.	Human Tissue (Authorisation) (Scotland) Act 2019
Slovak Republic*	Opt-out	01 Jan 2005 to opt-out (soft)<br>29 April 2017 to hard opt-out	Slovakia has an opt-out system where all adults are presumed donors unless they have opted out.	Act No. 576/2004 Coll. on Healthcare<br>Act No. 317/2016 Coll. on Requirements and Procedures for the Collection and Transplantation of Human Organs, Human Tissues, and Human Cells
Switzerland	Opt-in	No	Switzerland uses an opt-in system. Donation is allowed with the deceased's written consent or, if absent, with next-of-kin approval based on the presumed will.	Federal Act on the Transplantation of Organs, Tissues and Cells (810.21)
Turkey	Opt-in	No	Turkey uses an opt-in system. However, in practice, even with a prior declaration, the family's consent is always required.	Organ and Tissue Transplantation Law (Law No. 2238, 1979, with amendments up to December 5, 2018)
USA	Opt-in	No	The USA uses an opt-in system. People can register via state donor registries or on their driver's license. Family consent is also typically sought.	Uniform Anatomical Gift Act (2006)
Wales	Opt-out	01 December 2015 to soft opt-out	Wales introduced an opt-out system in 2015. Consent is presumed, but families are consulted before donation.	Human Transplantation (Wales) Act 2013

***Notes*.** Switching dates indicate the point in time at which the policy change came into effect. The key difference between a soft and a hard opt-out system is the role of the family. In a soft opt-out system, the family of the deceased can still veto organ donation, effectively making the final decision theirs, whereas in a hard opt-out system, family objections are not permitted and donation proceeds unless the individual explicitly opted out during their lifetime. All links, last seen on July 8, 2025. Where possible, we linked to the English version of the legislation.

Figure S1.2: *Panel data for deceased and living organ donors*.

A Panel data on deceased donors　　**B** Panel data on living donors

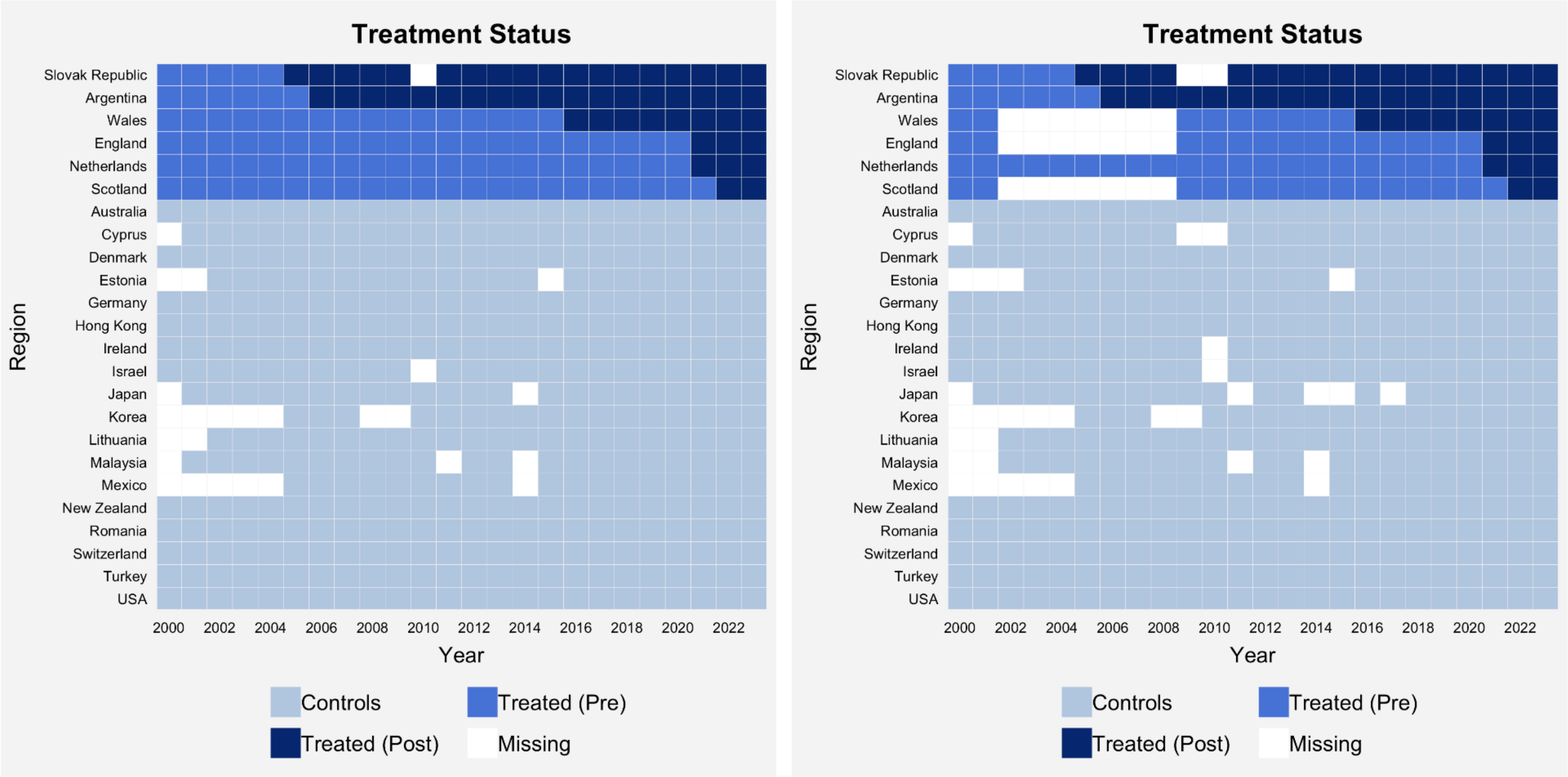

Notes. The Figure provides an overview of the panel data for our main analysis of deceased donors (Panel A) and living donors (Panel B) from countries that switched to opt-out default (treated) and maintained an opt-in default (controls) between 2000-2023. Treated countries have switched to opt-out default within the study period. As policy switches can occur within a running year, we treat the first year in which the policy is fully in place as the first year of the treated (post) period. The panels show the available panel data before data imputation.

Figure S1.3: *Model free time series plots for deceased and living donor rates.*

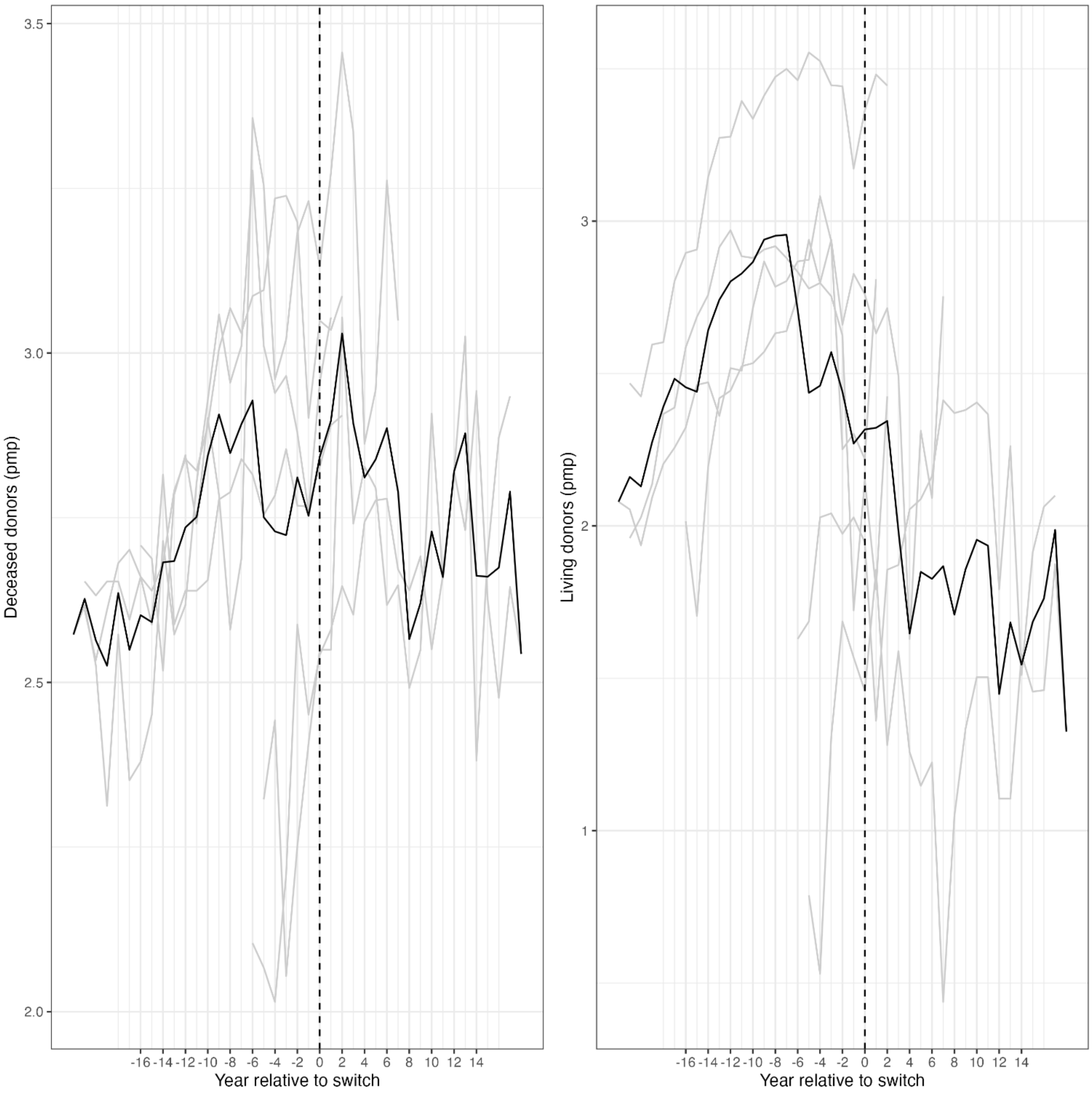

Notes: The donor rate is expressed as donors per million population (pmp). The graphs show the model free panel data for the countries that switched to opt-out within the study period. Grey lines reflect the development of donors per million population (pmp) for each country in the years relative to the switch. Black lines reflect the average development of donor numbers (pmp) for each country in the years relative to the switch.

Robustness checks.

To ensure the validity of our main findings, we conducted several robustness checks addressing three potential concerns: (1) the imputation of missing data, (2) the sampling strategy, and (3) the period of investigation, particularly the inclusion of the Covid years 2020–2022. An overview of the additional model specifications estimated as robustness checks is provided in Table S1.2.

Overall, **all models show consistent support for the direction of our main results**, thereby reinforcing the robustness of our key findings. Table S1.3 summarizes the main results across all robustness models, while Tables S1.3–S1.6 and Figures S1.3–S1.6 offer detailed insights into each specification.

Data imputation:

Our primary analysis employed *multiple imputation* to handle missing data in organ donor statistics. To verify that our findings are not an artifact of this imputation, we re-estimated the model without imputing missing data. The results for model (2) show that our findings remain directionally stable, indicating that data imputation does not drive our main effects (see Table S1.3 for an overview and Table S1.4 and Figure S1.4 for detailed results).

Sampling strategy:

Our main analysis is based on a *balanced* sampling strategy that aimed to reconcile two goals: (1) including only countries that meet our definition of switching from opt-in to opt-out, and (2) maintaining a broad sample to enhance the generalizability of our findings. As part of this strategy, we included two countries — the Slovak Republic and Argentina — which not only switched to opt-out during the study period but also later introduced hard opt-out policies, removing the possibility of family objection (see Table S1.2).

To test whether this balanced approach influenced our results, we conducted a more *conservative* analysis excluding these two cases. The results of this test are shown in model (3), and they support our hypothesis both directionally and statistically (see Table S1.3 for an overview, and Table S1.5 and Figure S1.5 for details). Notably, the observed crowding-out effects are even stronger under the conservative sampling strategy, suggesting that the effects are more pronounced in cases that closely match our core definition of the policy shift. In sum, these results provide additional support for our crowding-out hypothesis.

Period of investigation:

Our difference-in-differences approach measures relative changes and therefore inherently adjusts for general effects of the Covid-19 pandemic. Nevertheless, we conducted additional analyses excluding the pandemic period, restricting the data to pre-2020. Notably, this restriction in the period of investigation not only alters the temporal scope but also changes the sample composition by excluding England, the Netherlands, and Scotland—countries that implemented opt-out policies between 2020 and 2021 (see Table S1.1). Consequently, any differences observed between models are driven not only by changes in the period of investigation but also by this shift in sample composition.

Furthermore, variations in country-level effect sizes between models including or excluding 2020–2023 also stem from the nature of the estimated treatment effects. Since the average treatment effects on the

treated (ATTs) reflect annualized changes in donor statistics, adding or removing years can influence the magnitude of these averages.

In sum, we again observe directional support for our hypothesis when excluding the pandemic years and the countries that switched during that period (see Table S1.3 for an overview and Table S1.6 and Figure S1.6 for detailed results). Although effect sizes are smaller in these models, this is unlikely due to Covid-related disruptions because we do not observe a uniform decline across countries during the pandemic period, but rather substantial variation in the magnitude of effects. Specifically, we find a reduction in living donor rates of –7.43 pmp in England and –8.94 pmp in Scotland, compared to a more modest decrease of –2.22 pmp in the Netherlands. This heterogeneity suggests that the observed effects are more plausibly driven by differences in policy implementation rather than by the pandemic itself. In sum, this analysis again provides evidence for the robustness of our findings.

Table S1.2: *Robustness checks: Overview of model specifications.*

Model (#)	Data imputation	Sampling strategy	Period of investigation	Switches from opt-in to opt-out included in the model estimation (month/year)
(1)	yes	Balanced	2000-2023	Slovak Republic (01/2005), Argentina (12/2005), Wales (12/2015), England (05/2020), Netherlands (07/2020), Scotland (03/2021)
(2)	no	Balanced	2000-2023	Slovak Republic (01/2005), Argentina (12/2005), Wales (12/2015), England (05/2020), Netherlands (07/2020), Scotland (03/2021)
(3)	yes	Conservative	2000-2023	Wales (12/2015), England (05/2020), Netherlands (07/2020), Scotland (03/2021)
(4)	yes	Balanced	2000-2019	Slovak Republic (01/2005), Argentina (12/2005), Wales (12/2015)

Table S1.3: *Robustness checks: Overview of key findings across all robustness checks.*

ATT(average) on	(1)	(2)	(3)	(4)
Deceased donors (pmp)	**1.21**<br>(-0.70, 3.12)	**1.65**<br>(-1.21, 4.50)	**0.68**<br>(-1.82, 3.18)	**4.39*****<br>(3.32, 5.46)
Living donors (pmp)	**-4.59***<br>(-8.63, -0.55)	**-7.68†**<br>(-16.06, 0.71)	**-4.68*****<br>(-7.30, -2.06)	**-2.42**<br>(-10.36, 5.52)
Total effect (pmp)	**-3.38**<br>(-8.37, 1.62)	**-6.00**<br>(-13.50, 1.50)	**-4.00†**<br>(-8.66, 0.66)	**1.97**<br>(-6.45, 10.38)
Data imputation	Yes	No	Yes	Yes
Sampling strategy	Balanced	Balanced	Conservative	Balanced
Period of investigation	2000-2023	2000-2023	2000-2023	2000-2019

Notes. See Table S1.1 and accompanying text for more information on model specifications. pmp = per million population. ATT = Average Treatment Effect on the Treated. ATTs represent the average annual donor rate change (pmp) after adopting an opt-out policy for the switching countries included in the respective model. Significance levels: † $P < 0.1$, * $P < 0.05$, ** $P < 0.01$, *** $P < 0.001$

Table S1.4: *Robustness check model (2): Difference-in-differences model results (no imputation, balanced sampling, 2000-2023).*

	Deceased donors (pmp)			Living donors (pmp)			Aggregate total effects (pmp)		
**Term**	**Estimate**	**95% CI**	***P***	**Estimate**	**95% CI**	***P***	**Estimate**	**95% CI**	***P***
**ATT(Average)**	**1.65**	**(-1.21, 4.50)**	**0.258**	**-7.68**	**(-16.06, 0.71)**	**0.073**	**-6.00**	**(-13.50, 1.50)**	**0.117**
ATT(2005): Slovak Rep.	-1.52	(-5.57, 2.53)	0.462	-5.39	(-22.17, 11.4)	0.529	-7.74	(-21.9, 6.43)	0.284
ATT(2006): Argentina	4.44	(-0.63, 9.52)	0.086	-10.24	(-26.97, 6.49)	0.230	-7.16	(-23.45, 9.13)	0.389
ATT(2016): Wales	5.32	(2.68, 7.97)	<0.001	-5.40	(-12.1, 1.3)	0.114	-0.24	(-5.85, 5.37)	0.934
ATT(2021): England	-1.59	(-4.62, 1.45)	0.746	-11.48	(-17.64, -5.31)	<0.001	-12.21	(-17.34, -7.07)	<0.001
ATT(2021): Netherlands	2.51	(0.08, 4.94)	0.043	-3.48	(-6.74, -0.22)	0.036	-0.08	(-4.28, 4.11)	0.969
ATT(2022): Scotland[a]	0.71	(-2.47, 3.9)	0.660	-10.08	(-16.62, -3.54)	0.003	-8.55	(-13.67, -3.43)	0.001

Notes. ATT = Average Treatment Effect on the Treated. ATTs represent the annual average change in donors (per million people) due to switching to an opt-out policy. The table reports country-specific ATTs, interpreted as the average effect for each switching cohort. The year in parentheses indicates the first full year of implementation. [a] Although Scotland switched in March 2021, we treat 2021 as the first full year of implementation because the UK reporting year runs from April to March.

Figure S1.4: *Robustness check model (2): Difference-in-differences model results (no imputation, balanced sampling, 2000-2023).*

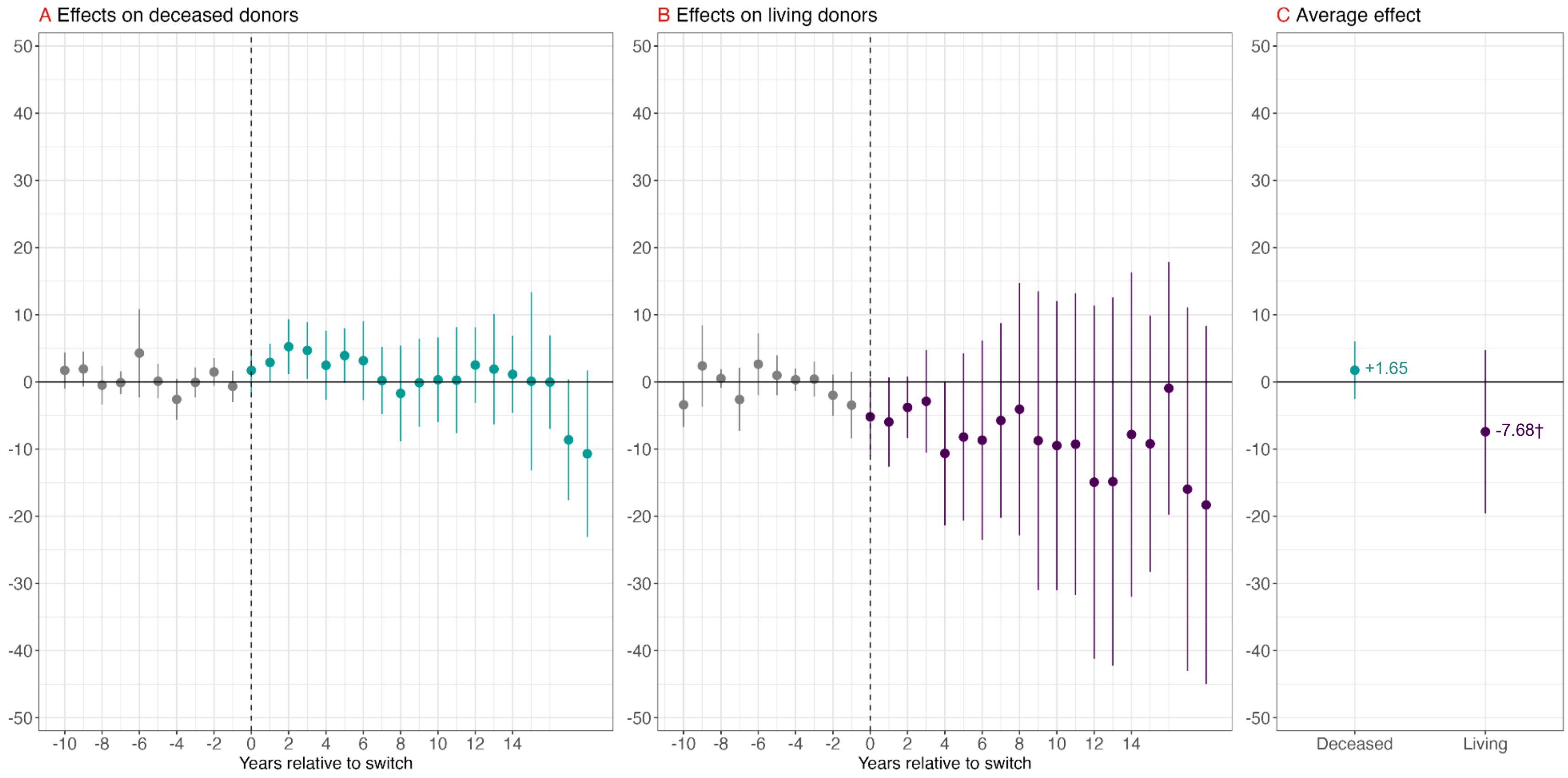

Notes. Results of a difference-in-differences model with staggered treatment adoption. The vertical dashed lines in panel **A** and **B** reflect the timepoint of the policy switch. Panel **C** shows average annual post-adoption effects. Error bars represent the 95% confidence interval for each point estimate. Significance levels in panel **C**: † $P < 0.1$, * $P < 0.05$, ** $P < 0.01$, *** $P < 0.001$.

Table S1.5: *Robustness check model (3): Difference-in-differences model results (conservative sampling, 2000-2023).*

	**Deceased donors (pmp)**			**Living donors (pmp)**			**Aggregate total effects (pmp)**		
**Term**	**Estimate**	**95% CI**	***P***	**Estimate**	**95% CI**	***P***	**Estimate**	**95% CI**	***P***
**ATT(Average)**	**0.68**	**(-1.82, 3.18)**	**0.593**	**-4.68**	**(-7.30, -2.06)**	**<0.001**	**-4.00**	**(-8.66, 0.66)**	**0.093**
ATT(2016): Wales	4.92	(3.14, 6.70)	<0.001	-3.64	(-8.33, 1.05)	0.128	1.28	(-3.94, 6.51)	0.631
ATT(2021): England	-3.25	(-4.86, -1.64)	<0.001	-7.38	(-8.97, -5.79)	<0.001	-10.63	(-12.92, -8.35)	<0.000
ATT(2021): Netherlands	1.61	(-0.30, 3.52)	0.098	-2.18	(-3.76, -0.60)	0.007	-0.57	(-3.23, 2.10)	0.677
ATT(2022): Scotland[a]	-0.56	(-2.28, 1.16)	0.523	-5.53	(-7.17, -3.88)	<0.001	-6.09	(-8.43, -3.74)	<0.000

Notes. ATT = Average Treatment Effect on the Treated. ATTs represent the annual average change in donors (per million people) due to switching to an opt-out policy. The table reports country-specific ATTs, interpreted as the average effect for each switching cohort. The year in parentheses indicates the first full year of implementation. [a] Although Scotland switched in March 2021, we treat 2021 as the first full year of implementation because the UK reporting year runs from April to March.

Figure S1.5: *Robustness check model (3): Difference-in-differences model results (conservative sampling, 2000-2023).*

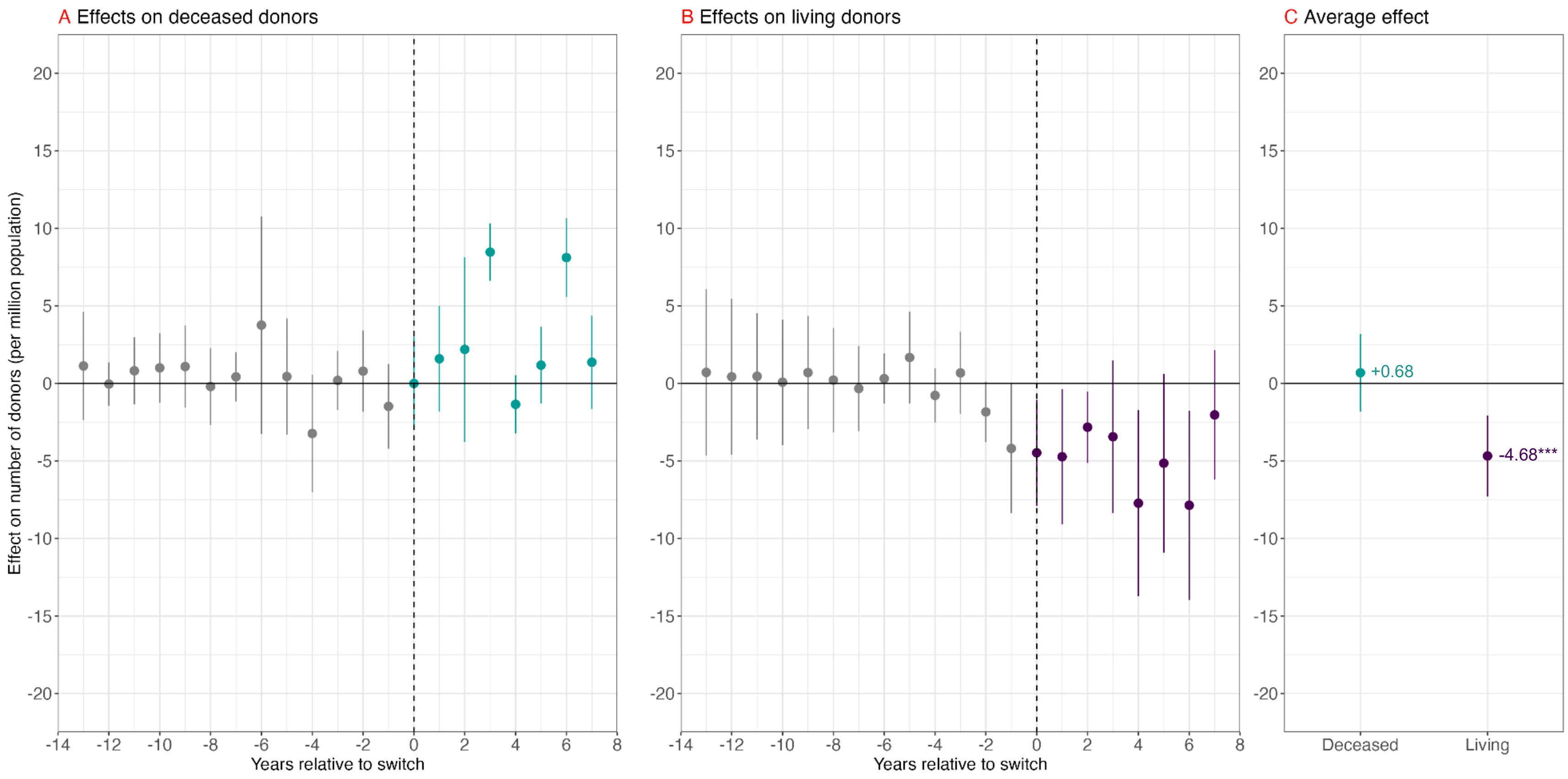

Notes. Results of a difference-in-differences model with staggered treatment adoption. The vertical dashed lines in panel **A** and **B** reflect the timepoint of the policy switch. Panel **C** shows average annual post-adoption effects. Error bars represent the 95% confidence interval for each point estimate. Significance levels in panel **C**: † $P < 0.1$, * $P < 0.05$, ** $P < 0.01$, *** $P < 0.001$.

Table S1.6: *Robustness check model (4): Difference-in-differences model results (balanced sampling, 2000-2019).*

	Deceased donors (pmp)			Living donors (pmp)			Aggregate total effects (pmp)		
**Term**	**Estimate**	**95% CI**	***P***	**Estimate**	**95% CI**	***P***	**Estimate**	**95% CI**	***P***
**ATT(Average)**	**4.39**	**(3.32, 5.46)**	**<0.001**	**-2.42**	**(-10.36, 5.52)**	**0.550**	**1.97**	**(-6.45, 10.38)**	**0.647**
ATT(2005): Slovak Rep.	0.33	(-1.41, 2.07)	0.712	-2.07	(-14.04, 9.90)	0.735	-1.74	(-13.86, 10.38)	0.778
ATT(2006): Argentina	5.31	(3.62, 7.01)	<0.001	-3.61	(-14.01, 6.79)	0.496	1.70	(-9.48, 12.88)	0.765
ATT(2016): Wales	7.52	(6.06, 8.99)	<0.001	-1.59	(-5.75, 2.57)	0.455	5.94	(1.25, 10.63)	0.013

Notes. ATT = Average Treatment Effect on the Treated. ATTs represent the annual average change in donors (per million people) due to switching to an opt-out policy. The table reports country-specific ATTs, interpreted as the average effect for each switching cohort. The year in parentheses indicates the first full year of implementation.

Figure S1.6: *Robustness check model (4): Difference-in-differences model results (balanced sampling, 2000-2019).*

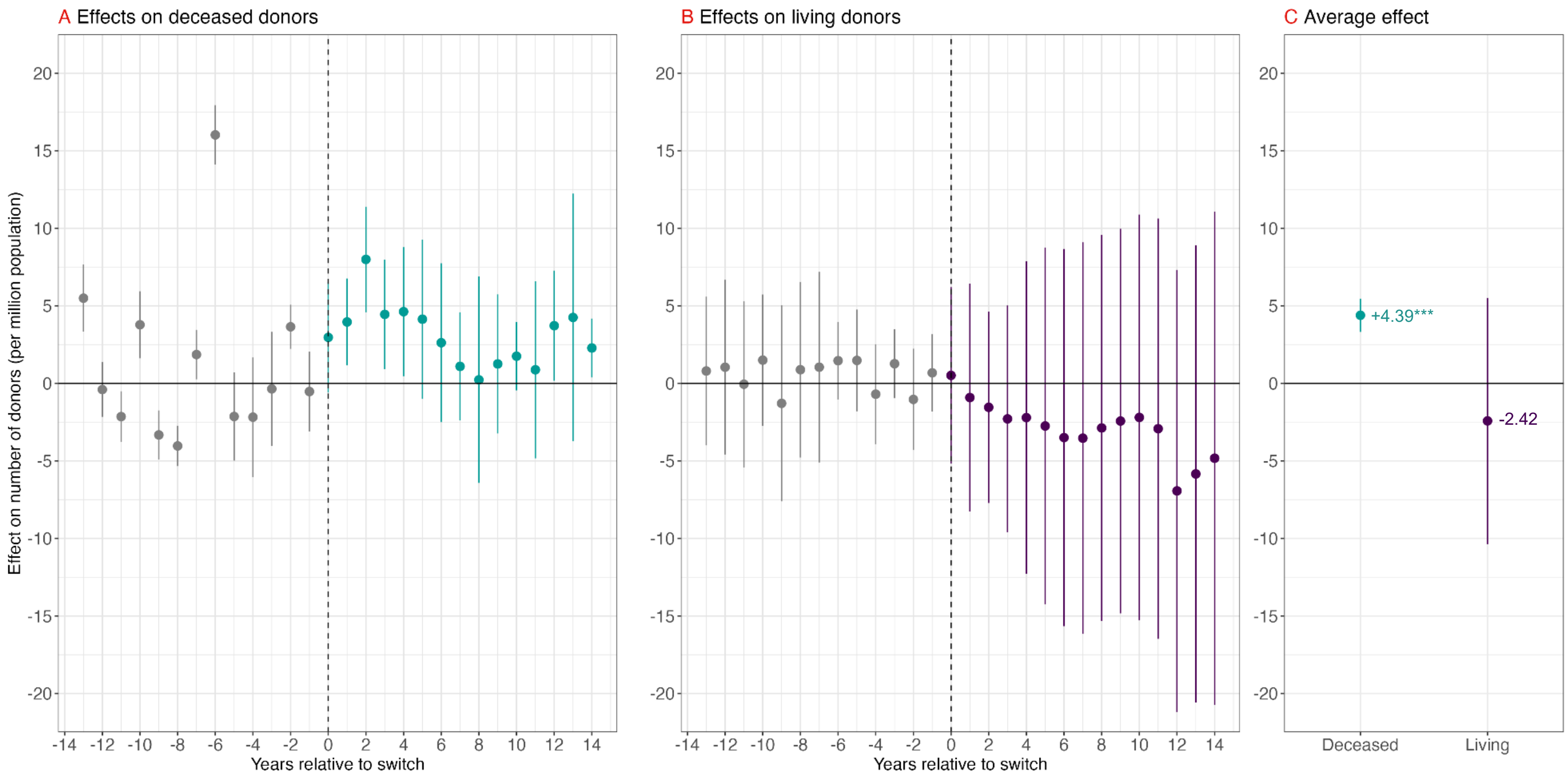

Notes. Results of a difference-in-differences model with staggered treatment adoption. The vertical dashed lines in panel **A** and **B** reflect the timepoint of the policy switch. Panel **C** shows average annual post-adoption effects. Error bars represent the 95% confidence interval for each point estimate. Significance levels in panel **C**: † $P < 0.1$, * $P < 0.05$, ** $P < 0.01$, *** $P < 0.001$.

Supplementary File 2: Materials and Supporting Analyses for Study 2

Table S2.1: *Survey design and measures.*

**Briefing on default consent and registration rates for deceased organ donations in Germany and Austria**	
**Germany (Opt-in)**	**Germany relies on an opt-in system for organ donation**. Official statistics indicate that approximately **36% of the population has actively registered**. These people are thus considered (in the event of their death) to be potential post-mortem organ donors.
**Austria (Opt-out)**	**Austria relies on an opt-out system for organ donation**. Official statistics indicate that **99.5% of the population are on the donor list** because they have not actively de-registered. These people are thus considered (in the event of their death) to be potential post-mortem organ donors.
**Briefing on living organ donation**	
**Briefing on living organ donation**	Now you get to know that someone is critically ill and is waiting for a kidney/lobe of a liver. The person needs either a post-mortem donation or a living donation. Assuming, a living donation is possible for you: Would you be willing to make this living donation?<br>Additionally, we have some further explanations concerning a living kidney/liver donation for you: A living kidney/liver donation has many advantages for the recipient…<br>[for kidney:] In this way, a better functional rate and a longer functional duration of the kidney can be achieved. A living donation would require an operation to remove the kidney.<br>[for liver:] The long waiting time for a liver of a deceased person can be shortened considerably. Sometimes the partial liver donation for a patient is also the only way to get a donor organ. The removal of a part of the liver from a living donor would require an operation.
**Dependent variables**	
**Willingness to Donate**	To what extent you would be willing to donate one of your kidneys/a part of your liver to:<br>(1 = Not at all likely, to 7 = Extremely likely)<br>• A close family member?<br>• A close friend?<br>• A remote relative?<br>• An acquaintance?<br>• A stranger?<br>*Note: All participants indicated their willingness to donate a kidney and a lobe of their liver. The sequences of appearance of the questions about liver and kidney donations were randomized.*
**Mediating variables** *(note that the sequences of constructs and items was randomized)*	
**Perceived Sufficiency of Supply**<br>**(α = 0. 839)**<br>(Evans and Ferguson 2014)	To what extent to do you agree with the following statements?<br>(1 = strongly disagree, to 7 = strongly agree)<br>• If I do not donate, it will certainly be easy to find another organ donor.<br>• I think that there are enough people willing to donate their organs. I don't have to do that.<br>• I think that the need for organs is already sufficiently covered by the number of existing donors.
**Perceived Reputation Building**<br>**(α = 0. 903)**<br>(Chell and Mortimer 2014)	To what extent to do you agree with the following statements? **(Cronbach's α = 0. 903)**<br>(1 = strongly disagree, to 7 = strongly agree)<br>• Donating organs improves the way I am perceived by others.<br>• Donating organs makes a good impression on other people.<br>• Donating organs gives me social approval.

**Covariates – Prosocial Attitude**	
**Reciprocity**<br>**(α = 0. 774)**<br>(Perugini et al. 2003)	To what extent do you agree with the following statements?<br>(1 = strongly disagree, to 7 = strongly agree)<br>• To help somebody is the best policy to be certain that s/he will help you in the future.<br>• I do not behave badly with others so as to avoid them behaving badly with me.<br>• I fear the reactions of a person I have previously treated badly.<br>• If I work hard, I expect it will be repaid.<br>• When I pay someone compliments, I expect that s/he in turn will reciprocate.<br>• I avoid being impolite because I do not want others being impolite with me.<br>• If I help tourists, I expect that they will thank me nicely.<br>• It is obvious that if I treat someone badly s/he will look for revenge.<br>• If I don't leave a good tip in a restaurant, I expect that in future I will not get good service.
**Philanthropy**<br>**(α = 0. 630)**<br>(Schuyt, Smit, Bekkers 2004)	To what extent do you agree with the following statements?<br>(1 = strongly disagree, to 7 = strongly agree)<br>• We have to leave this world a better place for the next generation.<br>• Each generation has to solve its own problems.[†] (reverse coded)<br>• Society is in danger because people are less concerned about each other nowadays.<br>• The world needs responsible citizens.<br>• The world community relies on international politics and corporations, and that is a good thing.[†] (reverse coded)<br>• I give money to charitable causes, no matter what the government does.<br>• Charity and public benefit should be supported by the government, and not by citizens and business corporations.[†] (reverse coded)
**Altruism**<br>**(α = 0. 875)**<br>(Rushton, Chrisjohn, and Fekken 1981)	Please tick the relevant box below that indicates how often, if at all, you have actually carried out the following actions.<br>(1 = never, 5 = very often)<br>• I have given directions to a stranger.<br>• I have given change for a banknote to a stranger.<br>• I have given money to a charity.<br>• I have given money to a stranger who needed it or asked me for it.<br>• I have donated goods or clothes to a charity.<br>• I have done voluntary work for a charity.<br>• I have donated blood.<br>• I have helped carry a stranger's belongings (books, parcels, etc.).<br>• I have delayed a lift and held the door open for a stranger.<br>• I have helped push a stranger's car that had broken down.<br>• I have allowed someone to go ahead of me in a queue (e.g. in the supermarket, for a photocopier).<br>• I have given a stranger a lift in my car.<br>• I have pointed out a clerk's error (e.g. in a bank, at the supermarket) in undercharging me for an item.<br>• I have let a neighbour who I didn't know too well borrow an item of some value to me (e.g. a dish, tools, etc.).<br>• I have bought charity Christmas cards deliberately because I knew it was for a good cause.<br>• I have voluntarily helped a workmate or classmate who I did not know that well with a task where my knowledge was greater.<br>• I have, before being asked, voluntarily looked after a neighbour's children or pets without being paid for it.<br>• I have offered to help a handicapped or elderly stranger across a street.<br>• I have offered my seat on a bus or train to a stranger who was standing.<br>• I have helped an acquaintance to move households.

Covariates – Health and Organ Donation	
**Know so. who donated organ**	• Do you know someone who has donated an organ by transplantation? (0 = no, 1 = yes)
**Know so. who received organ**	• Do you know someone who has received an organ by transplantation? (0 = no, 1 = yes)
**Know so. who is critically ill**	• Is one of your family members or close friends terminally critically ill? (0 = no, 1 = yes)
**Healthy enough**	• Do you feel healthy enough to be able to make a living donation? (1 = not at all, to 7 = completely)
**Trust in doctors**	• Do you trust in doctors and their expertise? (1 = not at all, to 7 = completely)
**Registration as deceased donor**	• I am registered as a potential post-mortem organ donor. (0 = no, 1 = yes)
**Covariates – Demographics**	
**Age**	• How old are you? ___ years
**Gender**	• With which gender do you identify? (0 = female, 1 = male)
**Religion**	• Do you belong to a church or religious community? (0 = no, 1 = yes)

Notes. For each recipient, willingness to be a living donor was constructed as the mean of their willingness to donate a liver and kidney. We conducted exploratory (EFA) and confirmatory factor analyses (CFA) to assess the reliability and validity of the measurement scales for 'perceived supply sufficiency' and 'perceived reputation building'. Following the recommended thresholds in the literature (Bagozzi and Yi 2012; Marsh et al. 2004), items marked with † were discarded to ensure sufficient reliability and validity of our measures. The results remain robust with or without the inclusion of these items. The local and global fit indices indicate a very good model fit of the final measurement model. For the local fit indices, we find that indicator reliability (IR) exceeds .79 for all items and both constructs have high composite reliabilities (CR) (CR 'perceived sufficiency of supply to meet demand' = .84; CR 'perceived reputation building' = .90). The fact that both constructs are not significantly correlated ($r = -.05$; $p = .44$) offers indication for high discriminant validity (Fornell and Larcker 1981). The global fit indices further indicate a very good fit of the measurement model (RMSEA = < .01; 90%CI 0.00, .056; CFI = 1.00; SRMR = .02).

Table S2.2: *Correlations and means.*

	1	2	3	4	5	6	7	8	9	10	11	12	13	14	15	16	17	18	19
**1.** Willingness to donate to close family member	1																		
**2.** Willingness to donate to close friend	0.563**	1																	
**3.** Willingness to donate to distant relative	0.483**	0.717**	1																
**4.** Willingness to donate to acquaintance	0.423**	0.769**	0.874**	1															
**5.** Willingness to donate to stranger	0.252**	0.607**	0.754**	0.810**	1														
**6.** Sufficiency of supply	-0.171**	-0.231**	-0.177**	-0.123**	-0.092	1													
**7.** Reputation building	0.135**	0.227**	0.277**	0.290**	0.256**	-0.049	1												
**8.** Reciprocity	0.113*	0.081	0.109*	0.143**	0.073	0.086	0.388**	1											
**9.** Philanthropy	0.169**	0.149**	0.176**	0.127**	0.167**	-0.272**	0.070	0.070	1										
**10.** Altruism	0.203**	0.247**	0.288**	0.285**	0.275**	-0.062	0.176**	0.140**	0.260**	1									
**11.** Knowing s.o. who donated an organ	0.042	-0.034	-0.068	-0.099*	-0.140**	-0.031	-0.025	0.005	-0.011	-0.160**	1								
**12.** Knowing s.o. who received an organ	-0.070	-0.050	-0.053	-0.060	-0.049	0.134**	0.021	0.025	-0.090	-0.213**	0.381**	1							
**13.** Knowing s.o. who is critically ill	0.074	0.033	0.021	0.036	0.010	0.025	0.010	0.029	-0.028	-0.067	0.064	0.131**	1						
**14.** Feeling healthy enough to donate	0.163**	0.212**	0.205**	0.194**	0.170**	-0.147**	0.204**	0.086	0.185**	0.171**	0.072	0.020	0.072	1					
**15.** Trust in doctors	0.121*	0.150**	0.237**	0.223**	0.154**	-0.201**	0.237**	0.154**	0.201**	0.167**	-0.072	-0.126**	0.025	0.239**	1				
**16.** Registration as deceased organ donor	0.131**	0.169**	0.134**	0.172**	0.174**	-0.170**	0.140**	-0.002	0.205**	0.166**	-0.101*	-0.104*	-0.034	0.244**	0.183**	1			
**17.** Age	-0.093	-0.248**	-0.132**	-0.136**	-0.101*	-0.016	-0.201**	-0.185**	-0.019	0.046	-0.109*	-0.106*	0.009	-0.383**	0.017	-0.153**	1		
**18.** Gender	0.186**	0.121*	0.108*	0.077	0.127**	-0.187**	0.010	0.013	0.115*	-0.023	-0.013	-0.089	0.041	0.069	-0.017	0.097*	-0.120*	1	
**19.** Religious	0.083	0.063	0.133**	0.093	0.084	-0.038	0.098*	0.156**	0.068	0.172**	-0.087	-0.161**	-0.095*	0.072	0.099*	0.087	-0.137**	0.115*	1
**Means**	**5.816**	**4.568**	**3.6241**	**3.453**	**2.526**	**2.270**	**3.153**	**4.141**	**4.724**	**2.731**	**1.880**	**1.760**	**1.870**	**4.430**	**5.000**	**0.400**	**44.170**	**0.510**	**0.563**

Notes: The Table shows the statistics for the total sample (N_{Total} = 435). The coefficients in the table are Pearson correlations (except for the means for each construct in the bottom row). **. Correlation is significant at the 0.01 level (2-tailed). *. Correlation is significant at the 0.05 level (2-tailed). No correction for multiple comparison was undertaken. We used SPSS Version 28.0.1.1. to generate this analysis. Variable coding: gender (0 = female, 1 = male), religious (0 = not religious, 1 = religious).

Table S2.3: *Effect of opt-out default policy on willingness to donate a living organ.*

	(1) Familial donation	(2) Altruistic directed	(3) Altruistic non-directed	(4) All donations (index)
**Focal Relationship**				
Opt-out default policy	0.038	-0.418**	-0.347*	-0.312*
	(0.140)	(0.146)	(0.158)	(0.129)
**Covariates – prosocial attitudes**				
Reciprocity	0.056	-0.008	-0.035	-0.001
	(0.080)	(0.088)	(0.082)	(0.075)
Philanthropy	0.183*	0.156*	0.144+	0.159*
	(0.076)	(0.074)	(0.08)	(0.065)
Altruism	0.348**	0.637***	0.592***	0.570***
	(0.118)	(0.132)	(0.135)	(0.113)
**Covariates – health and organ donation**				
Know so. who donated organ	0.404	-0.262	-0.672*	-0.211
	(0.265)	(0.261)	(0.264)	(0.228)
Know so. who received organ	-0.180	0.058	0.244	0.048
	(0.168)	(0.193)	(0.19)	(0.164)
Know so. who is critically ill	0.348	0.175	0.064	0.188
	(0.231)	(0.226)	(0.217)	(0.197)
Healthy enough	0.042	0.052	0.055	0.051
	(0.044)	(0.045)	(0.044)	(0.039)
Trust in doctors	0.037	0.142*	0.068	0.106*
	(0.059)	(0.062)	(0.062)	(0.054)
Registration as deceased donor	0.135	0.179	0.262	0.187
	(0.148)	(0.163)	(0.171)	(0.140)
**Covariates - demographics**				
Age	-0.004	-0.017**	-0.008	-0.013**
	(0.006)	(0.005)	(0.005)	(0.005)
Gender	-0.484**	-0.279+	-0.369*	-0.338*
	(0.145)	(0.152)	(0.156)	(0.131)
Religious	0.038	0.074	0.056	0.063
	(0.148)	(0.154)	(0.156)	(0.132)

Notes. Total sample size is $N_{Total} = 435$. The sample size for Germany is $N_{\text{Germany}} = 210$. The sample size for Austria is $N_{\text{Austria}} = 225$. Significance levels: + $P < 0.1$, * $P < 0.05$, ** $P < 0.01$, *** $P < 0.001$. *P*-values are two-tailed. We relied on a path regression analysis to estimate all effects simultaneously. All models were estimated using a maximum likelihood estimation with robust standard errors using *M*plus version 8.9. No correction for multiple comparisons was used. We used dummy variable coding to code default policy (0 = opt-in (Germany), 1 = opt-out (Austria)), gender (0 = female, 1 = male), and religious (0 = non-religious, 1 = religious). Estimates are unstandardized regression coefficients. Following the pre-registration, we analyzed the effects including the effects of all covariates on each dependent variable to account for the influence of relevant influence factors of organ donation decisions identified by prior literature.

Table S2.4: *Effects of opt-out default policy on willingness to donate a living organ for different types of recipients.*

	Estimate	(S.E.)	*p*-value
**Effect of opt-out default policy on**			
Willingness to donate to core family member	0.038	(0.140)	0.786
Willingness to donate to close friend	-0.317	(0.160)	0.047
Willingness to donate to distant relative	-0.466	(0.162)	0.004
Willingness to donate to acquaintance	-0.470	(0.159)	0.003
Willingness to donate to stranger	-0.347	(0.158)	0.028

Notes. Total sample size is $N_{Total} = 435$. The sample size for Germany is $N_{\text{Germany}} = 210$. The sample size for Austria is $N_{\text{Austria}} = 225$. We relied on a path regression analysis to estimate all effects simultaneously. All models were estimated using a maximum likelihood estimation with robust standard errors using *M*plus version 8.9. No correction for multiple comparisons was used. We used dummy variable coding to test the effects of the default policy (0 = opt-in (Germany), 1 = opt-out (Austria)) on willingness to donate living organs for the respective recipients. Estimates are unstandardized regression coefficients. *P*-values are two-tailed. Following the pre-registration, we analyzed the effects including the effects of all covariates on each dependent variable to account for the influence of relevant influence factors of organ donation decisions identified by prior literature. Results for covariate effects are not included to facilitate interpretation of the core effects.

Figure S2.1: *Differences in willingness to become a living organ donor between Germany (opt-in, 36% registered) and Austria (opt-out, 99.5% registered).*

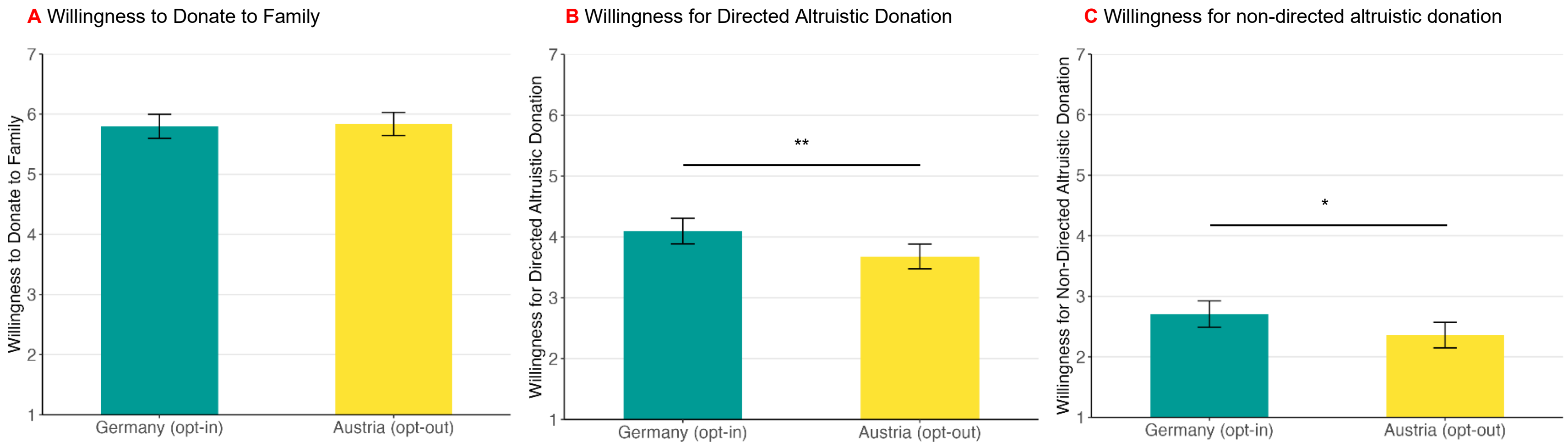

Notes. Significance levels: * $P < 0.05$, ** $P < 0.01$, *** $P < 0.001$. *P*-values are two-tailed. The plots show differences in willingness to become a living organ donor between Germany (opt-in, 36% registered) and Austria (opt-out, 99.5% registered) regarding the willingness to become a familial (Panel A), directed altruistic (Panel B), and non-directed altruistic living organ donor (Panel C). The horizontal lines and stars indicate which conditions are significantly different from one another. The effects associated with the significance levels reflect the difference between two conditions conditional on all covariate effects. To highlight pairwise comparisons between combinations we estimated separate versions of the same model using different levels of the experimental manipulations as the baseline. All covariates are demeaned to facilitate interpretation.

Figure S2.2: *Differences in perceptions of sufficiency of organ supply and reputational gain between Germany (opt-in, 36% registered) and Austria (opt-out, 99.5% registered)*

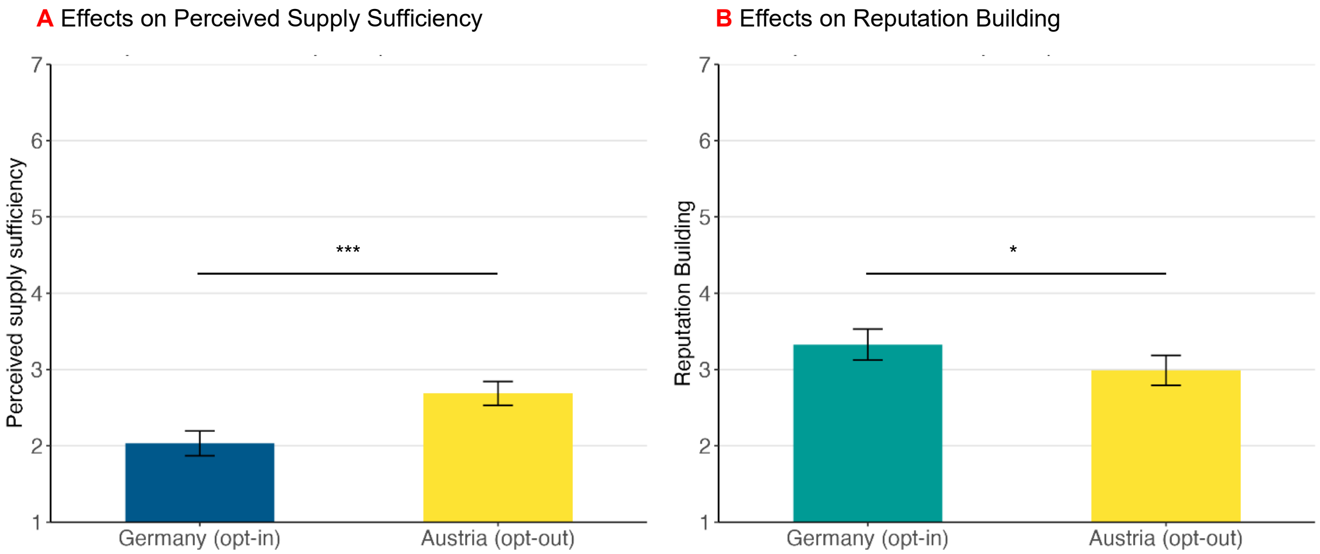

Notes. Significance levels: * $P < 0.05$, ** $P < 0.01$, *** $P < 0.001$. *P*-values are two-tailed. The plots show differences between Germany (opt-in, 36% registered) and Austria (opt-out, 99.5% registered) regarding perceptions of sufficiency of organ supply (Panel A) and perceived reputational gain from being a deceased organ donor (Panel B). The horizontal lines and stars indicate which conditions are significantly different from one another. The effects associated with the significance levels reflect the difference between two conditions conditional on all covariate effects. To highlight pairwise comparisons between combinations we estimated separate versions of the same model using different levels of the experimental manipulations as the baseline. All covariates are demeaned to facilitate interpretation.

Table S2.5: *Parallel mediation analysis for Study 2.*

**Tested relationships**	**(1) Supply Sufficiency**	**(2) Reputation building**	**(3) Familial donation**	**(4) Altruistic directed**	**(5) Altruistic non-directed**
Opt-out default policy	0.655***<br>(0.119)	-0.338*<br>(0.146)	0.138<br>(0.148)	-0.279+<br>(0.156)	-0.293+<br>(0.168)
Sufficiency of supply			-0.127*<br>(0.065)	-0.124*<br>(0.059)	0.011<br>(0.064)
Reputation building			0.048<br>(0.046)	0.171**<br>(0.052)	0.181***<br>(0.053)
**Mediation analysis**					
**Specific indirect effects**					
Indirect effect via sufficiency of supply			-0.084+<br>(0.046)	-0.081+<br>(0.041)	0.007<br>(0.043)
Indirect effect via reputation building			-0.016<br>(0.019)	-0.058+<br>(0.031)	-0.061+<br>(0.032)
**Total indirect effects**					
Total indirect effect			-0.100+<br>(0.051)	-0.139**<br>(0.05)	-0.054<br>(0.052)
Total direct and indirect effect			0.038<br>(0.143)	-0.418**<br>(0.151)	-0.347*<br>(0.162)

Notes. Total sample size is $N_{Total} = 435$. The sample size for Germany is $N_{\text{Germany}} = 210$. The sample size for Austria is $N_{\text{Austria}} = 225$. Significance levels: + $P < 0.1$, * $P < 0.05$, ** $P < 0.01$, *** $P < 0.001$. *P*-values are two-tailed. We relied on a path regression analysis to estimate all effects simultaneously. All models were estimated using a maximum likelihood estimation with robust standard errors using *M*plus version 8.9. No correction for multiple comparisons was used. We used dummy variable coding to test the effects of the default policy (0 = Germany, opt-in; 1 = Austria, opt-out) on willingness to donate living organs (index). The index was created by calculating the average for all recipients. Sufficiency of supply = perceived sufficiency of supply to meet demand. Reputation building = perceived reputation building. Estimates are unstandardized regression coefficients. Following the pre-registration, we analyzed the effects including the effects of all covariates on each dependent variable to account for the influence of relevant influence factors of organ donation decisions identified by prior literature. Results for covariate effects are not included to facilitate interpretation of the core effects.

Supplementary File 3: Materials and Supporting Analyses for Study 3

Table S3.1: *Survey design, experimental manipulations, and measures.*

**Experimental manipulation of default consent and organ supply (i.e., registration rates)**	
**Opt-in, low registration rate**	**Imagine that you live in a country with an opt-in policy for deceased organ donation**. Assume that **15% of the population has actively registered**. These people can be considered (in case of their death) as potential post-mortem organ donors.
**Opt-in, high registration rate**	**Imagine that you live in a country with an opt-in policy for deceased organ donation**. Assume that **85% of the population has actively registered**. These people can be considered (in case of their death) as potential post-mortem organ donors.
**Opt-out, low registration rate**	**Imagine that you live in a country with an opt-out policy for deceased organ donation**. Assume that **15% of the population are registered as deceased organ donors** as they have not actively de-registered. These people can be considered (in case of their death) as potential post-mortem organ donors.
**Opt-out, high registration rate**	**Imagine that you live in a country with an opt-out policy for deceased organ donation**. Assume that **85% of the population are registered as deceased organ donors** as they have not actively de-registered. These people can be considered (in case of their death) as potential post-mortem organ donors.
**Briefing on living organ donation**	
**Briefing on living organ donation**	Now you get to know that someone is critically ill and is waiting for a kidney/lobe of a liver. The person needs either a post-mortem donation or a living donation. Assuming, a living donation is possible for you: Would you be willing to make this living donation?<br>Additionally, we have some further explanations concerning a living kidney/liver donation for you: A living kidney/liver donation has many advantages for the recipient…<br>[for kidney:] In this way, a better functional rate and a longer functional duration of the kidney can be achieved. A living donation would require an operation to remove the kidney.<br>[for liver:] The long waiting time for a liver of a deceased person can be shortened considerably. Sometimes the partial liver donation for a patient is also the only way to get a donor organ. The removal of a part of the liver from a living donor would require an operation.
**Dependent variables**	
**Willingness to Donate**	To what extent you would be willing to donate one of your kidneys/a part of your liver to:<br>(1 = Not at all likely, to 7 = Extremely likely)<br>• A close family member?<br>• A close friend?<br>• A remote relative?<br>• An acquaintance?<br>• A stranger?<br>*Note: All participants indicated their willingness to donate a kidney and a lobe of their liver. The sequences of appearance of the questions about liver and kidney donations were randomized.*
**Mediating variables** *(note that the sequences of constructs and items was randomized)*	
**Perceived Sufficiency of Supply**<br>**(α = 0. 840)**<br>(Evans and Ferguson 2014)	To what extent to do you agree with the following statements?<br>(1 = strongly disagree, to 7 = strongly agree)<br>• If I do not donate, it will certainly be easy to find another organ donor.<br>• I think that there are enough people willing to donate their organs. I don't have to do that.<br>• I think that the need for organs is already sufficiently covered by the number of existing donors.

**Perceived Reputation Building** **(α = 0. 915)** (Chell and Mortimer 2014)	To what extent to do you agree with the following statements?<br>(1 = strongly disagree, to 7 = strongly agree)<br>• Donating organs improves the way I am perceived by others.<br>• Donating organs makes a good impression on other people.<br>• Donating organs gives me social approval.
**Covariates – Prosocial Attitude**	
**Reciprocity** **(α = 0. 773)** (Perugini et al. 2003)	To what extent do you agree with the following statements?<br>(1 = strongly disagree, to 7 = strongly agree)<br>• To help somebody is the best policy to be certain that s/he will help you in the future.<br>• I do not behave badly with others so as to avoid them behaving badly with me.<br>• I fear the reactions of a person I have previously treated badly.<br>• If I work hard, I expect it will be repaid.<br>• When I pay someone compliments, I expect that s/he in turn will reciprocate.<br>• I avoid being impolite because I do not want others being impolite with me.<br>• If I help tourists, I expect that they will thank me nicely.<br>• It is obvious that if I treat someone badly s/he will look for revenge.<br>• If I don't leave a good tip in a restaurant, I expect that in future I will not get good service.
**Philanthropy** **(α = 0. 631)** (Schuyt, Smit, Bekkers 2004)	To what extent do you agree with the following statements?<br>(1 = strongly disagree, to 7 = strongly agree)<br>• We have to leave this world a better place for the next generation.<br>• Each generation has to solve its own problems.† (reverse coded)<br>• Society is in danger because people are less concerned about each other nowadays.<br>• The world needs responsible citizens.<br>• The world community relies on international politics and corporations, and that is a good thing.† (reverse coded)<br>• I give money to charitable causes, no matter what the government does.<br>• Charity and public benefit should be supported by the government, and not by citizens and business corporations.† (reverse coded)
**Altruism** **(α = 0. 873)** (Rushton, Chrisjohn, and Fekken 1981)	Please tick the relevant box below that indicates how often, if at all, you have actually carried out the following actions.<br>(1 = never, 5 = very often)<br>• I have given directions to a stranger.<br>• I have given change for a banknote to a stranger.<br>• I have given money to a charity.<br>• I have given money to a stranger who needed it or asked me for it.<br>• I have donated goods or clothes to a charity.<br>• I have done voluntary work for a charity.<br>• I have donated blood.<br>• I have helped carry a stranger's belongings (books, parcels, etc.).<br>• I have delayed a lift and held the door open for a stranger.<br>• I have helped push a stranger's car that had broken down.<br>• I have allowed someone to go ahead of me in a queue (e.g. in the supermarket, for a photocopier).<br>• I have given a stranger a lift in my car.<br>• I have pointed out a clerk's error (e.g. in a bank, at the supermarket) in undercharging me for an item.<br>• I have let a neighbour who I didn't know too well borrow an item of some value to me (e.g. a dish, tools, etc.).<br>• I have bought charity Christmas cards deliberately because I knew it was for a good cause.<br>• I have voluntarily helped a workmate or classmate who I did not know that well with a task where my knowledge was greater.<br>• I have, before being asked, voluntarily looked after a neighbour's children or pets without being paid for it.

	• I have offered to help a handicapped or elderly stranger across a street.<br>• I have offered my seat on a bus or train to a stranger who was standing.<br>• I have helped an acquaintance to move households.
**Covariates – Health and Organ Donation**	
**Know so. who donated organ**	• Do you know someone who has donated an organ by transplantation? (0 = no, 1 = yes)
**Know so. who received organ**	• Do you know someone who has received an organ by transplantation? (0 = no, 1 = yes)
**Know so. who is critically ill**	• Is one of your family members or close friends terminally critically ill? (0 = no, 1 = yes)
**Healthy enough**	• Do you feel healthy enough to be able to make a living donation? (1 = not at all, to 7 = completely)
**Trust in doctors**	• Do you trust in doctors and their expertise? (1 = not at all, to 7 = completely)
**Registration as deceased donor**	• I am registered as a potential post-mortem organ donor. (0 = no, 1 = yes)
**Covariates – Demographics**	
**Age**	• How old are you? ___ years
**Gender**	• With which gender do you identify? (0 = female, 1 = male)
**Religion**	• Do you belong to a church or religious community? (0 = no, 1 = yes)

Notes. For each recipient, willingness to be a living donor was constructed as the mean of their willingness to donate a liver and kidney. We conducted exploratory (EFA) and confirmatory factor analyses (CFA) to assess the reliability and validity of the measurement scales for 'perceived sufficiency of supply to meet demand' and 'perceived reputation building'. Following the recommended thresholds in the literature (Bagozzi and Yi 2012; Marsh et al. 2004), items marked with † were discarded to ensure sufficient reliability and validity of our measures. The results remain robust with or without the inclusion of these items. The local and global fit indices indicate a very good model fit of the final measurement model. For the local fit indices, we find that indicator reliability (IR) exceeds .74 for all items and both constructs have high composite reliabilities (CR) (CR 'perceived supply sufficiency' = .84; CR 'perceived reputation building' = .92). The correlation between both mediators is rather small ($r = 0.09$; $p < .01$) and the criterion by Fornell and Larcker (1981) clearly indicates a high discriminant validity, as the squared correlation between both constructs does not exceed the average variance extracted of each construct. The global fit indices attest a very good fit of the measurement model (RMSEA = < .05; 90 Percent CI [0.00; .031]; CFI = 1.00; SRMR = .01).

Table S3.2: *Descriptive statistics for each experimental group (n = 1,721).*

Variable	Opt-in, low registration rate (n=422) Mean (SD)	Opt-in, high registration rate (n=420) Mean (SD)	Opt-out, low registration rate (n=422) Mean (SD)	Opt-out, high registration rate (n=457) Mean (SD)
**DVs: Willingness to donate**				
Close family member	5.664 (1.701)	5.782 (1.640)	5.715 (1.612)	5.683 (1.610)
Close friend	4.429 (1.888)	4.620 (1.804)	4.513 (1.773)	4.309 (1.817)
Distant relative	3.619 (1.788)	3.668 (1.769)	3.523 (1.716)	3.357 (1.745)
Acquaintance	3.514 (1.757)	3.545 (1.748)	3.429 (1.751)	3.217 (1.698)
Stranger	2.576 (1.727)	2.569 (1.591)	2.409 (1.650)	2.357 (1.681)
Index (all recipients)	3.960 (1.490)	4.037 (1.481)	3.918 (1.458)	3.784 (1.447)
**Mediators**				
Sufficiency of Supply	2.074 (1.173)	2.390 (1.313)	2.168 (1.146)	2.415 (1.339)
Reputation Building	3.073 (1.717)	3.162 (1.634)	3.093 (1.684)	3.096 (1.638)
**Covariates**				
**Prosocial attitudes**				
Reciprocity	4.007 (1.056)	3.959 (0.976)	3.972 (0.995)	4.024 (0.962)
Philanthropy	4.669 (0.770)	4.801 (0.770)	4.757 (0.761)	4.745 (0.798)
Pure Altruism	2.729 (0.627)	2.749 (0.571)	2.747 (0.609)	2.778 (0.624)
**Health & organ donation**				
Knowing s.o. who donated an organ	0.140 (0.350)	0.120 (0.327)	0.100 (0.303)	0.110 (0.312)
Knowing s.o. who received an organ	0.230 (0.423)	0.210 (0.406)	0.230 (0.418)	0.220 (0.415)
Knowing s.o. who is critically ill	0.110 (0.315)	0.130 (0.340)	0.120 (0.324)	0.140 (0.343)
Feeling healthy enough to donate	4.430 (1.929)	4.470 (1.891)	4.450 (1.996)	4.460 (1.891)
Trust in doctors	4.950 (1.447)	4.910 (1.456)	4.920 (1.510)	4.980 (1.426)
Registration as deceased organ donor	0.230 (0.421)	0.240 (0.429)	0.300 (0.460)	0.320 (0.467)
**Demographics**				
Age	44.280 (15.198)	43.450 (14.074)	43.650 (14.658)	45.610 (15.341)
Gender	0.507 (0.501)	0.500 (0.501)	0.505 (0.501)	0.540 (0.499)
Religious	0.543 (0.499)	0.555 (0.498)	0.543 (0.499)	0.516 (0.500)

Notes. Coding of binary variables: gender (0 = female, 1 = male), all other binary variables (0 = no, 1 = yes); see Supplementary File 5, Table 8 for further details. Sufficiency of supply = perceived sufficiency of supply to meet demand. Reputation building = perceived reputation building.

Table S3.3: *Main and interaction effects of default policy and registration rate on willingness to make a (i) familial, (ii) directed altruistic, and (iii) non-directed altruistic living organ donation.*

	(1) Familial donation	(2) Familial donation	(3) Altruistic directed	(4) Altruistic directed	(5) Altruistic non-directed	(6) Altruistic non-directed
Opt-out default policy	-0.048<br>(0.072)	0.023<br>(0.103)	-0.200**<br>(0.069)	-0.057<br>(0.098)	-0.212**<br>(0.074)	-0.181+<br>(0.106)
High registration rate	0.019<br>(0.072)	0.089<br>(0.103)	-0.072<br>(0.069)	0.070<br>(0.097)	-0.040<br>(0.074)	-0.009<br>(0.102)
Opt-out default policy × high registration rate		-0.139<br>(0.143)		-0.280*<br>(0.138)		-0.061<br>(0.148)
**Covariates**						
Reciprocity	0.113**<br>(0.041)	0.115**<br>(0.040)	0.141***<br>(0.037)	0.144***<br>(0.037)	0.082*<br>(0.040)	0.083*<br>(0.040)
Philanthropy	0.204***<br>(0.040)	0.203***<br>(0.040)	0.215***<br>(0.037)	0.214***<br>(0.037)	0.149***<br>(0.038)	0.149***<br>(0.038)
Altruism	0.185**<br>(0.067)	0.185**<br>(0.067)	0.337***<br>(0.065)	0.337***<br>(0.065)	0.391***<br>(0.071)	0.391***<br>(0.071)
Know so. who donated organ	0.097<br>(0.116)	0.095<br>(0.116)	-0.085<br>(0.117)	-0.089<br>(0.116)	-0.181<br>(0.131)	-0.182<br>(0.131)
Know so. who received organ	-0.095<br>(0.090)	-0.095<br>(0.090)	-0.045<br>(0.091)	-0.045<br>(0.091)	-0.083<br>(0.100)	-0.083<br>(0.100)
Know so. who is critically ill	-0.005<br>(0.118)	-0.005<br>(0.118)	-0.017<br>(0.105)	-0.016<br>(0.106)	0.001<br>(0.114)	0.001<br>(0.114)
Healthy enough	0.193***<br>(0.024)	0.193***<br>(0.024)	0.192***<br>(0.022)	0.192***<br>(0.022)	0.122***<br>(0.022)	0.122***<br>(0.022)
Trust in doctors	0.149***<br>(0.031)	0.150***<br>(0.031)	0.193***<br>(0.027)	0.193***<br>(0.027)	0.134***<br>(0.027)	0.134***<br>(0.027)
Registration as deceased donor	0.202*<br>(0.078)	0.202*<br>(0.078)	0.291***<br>(0.082)	0.291***<br>(0.082)	0.299**<br>(0.093)	0.299**<br>(0.093)
Age	0.004<br>(0.003)	0.004<br>(0.003)	-0.010***<br>(0.003)	-0.010***<br>(0.003)	-0.007*<br>(0.003)	-0.007*<br>(0.003)
Gender	-0.305***<br>(0.075)	-0.304***<br>(0.075)	-0.035<br>(0.072)	-0.035<br>(0.072)	-0.024<br>(0.077)	-0.024<br>(0.077)
Religious	0.143*<br>(0.073)	0.142+<br>(0.073)	0.111<br>(0.07)	0.109<br>(0.070)	0.079<br>(0.075)	0.078<br>(0.075)
Country Austria	0.182*<br>(0.074)	0.180*<br>(0.074)	-0.173*<br>(0.070)	-0.177*<br>(0.070)	-0.372***<br>(0.075)	-0.373***<br>(0.075)

Notes. N = 1,721. Significance levels: + P < 0.1, * P < 0.05, ** P < 0.01, *** P < 0.001. P-values are two-tailed. Estimates show unstandardized regression coefficients. We relied on a path regression analysis to estimate the effects in the models (1), (3), and (5), as well as (2), (4), and (6) simultaneously. All models were estimated with a maximum likelihood estimator with robust standard errors using *M*plus version 8.9. No correction for multiple comparisons was used. We used dummy variable coding to test the effects of the default policy (0 = opt-in, 1 = opt-out) and registration rate (0 = 15%, 1 = 86%) on willingness to donate living organs. Directed altruistic donations is an index reflecting the average willingness to give to a friend, acquaintance, and distant relative. Following the pre-registration, we included the covariates in all models.

Table S3.4: *Multiple comparisons across experimental groups for (i) familial, (ii) directed altruistic, and (iii) non-directed altruistic living organ donation.*

	Familial donation			Directed altruistic donation			Non-directed altruistic donation		
	(1)	(2)	(3)	(4)	(5)	(6)	(7)	(8)	(9)
**Multiple comparisons**									
(Intercept)	5.690***	5.779***	5.712***	3.876***	3.946***	3.818***	2.588***	2.579***	2.407***
	(0.073)	(0.073)	(0.073)	(0.070)	(0.070)	(0.070)	(0.075)	(0.075)	(0.075)
Opt-in, 15% registered		-0.089	-0.023		-0.070	0.057		0.009	0.181+
		(0.103)	(0.103)		(0.099)	(0.099)		(0.106)	(0.106)
Opt-in, 85% registered	0.089		0.067	0.070		0.128	-0.009		0.172
	(0.103)		(0.103)	(0.099)		(0.099)	(0.106)		(0.106)
Opt-out, 15% registered	0.023	-0.067		-0.057	-0.128		-0.181+	-0.172	
	(0.103)	(0.103)		(0.099)	(0.099)		(0.106)	(0.106)	
Opt-out, 85% registered	-0.027	-0.116	-0.050	-0.267**	-0.337***	-0.209*	-0.251*	-0.242*	-0.070
	(0.101)	(0.101)	(0.101)	(0.097)	(0.097)	(0.097)	(0.104)	(0.104)	(0.104)
**Covariates**									
Reciprocity	0.115**	0.115**	0.115**	0.144***	0.144***	0.144***	0.083*	0.083*	0.083*
	(0.038)	(0.038)	(0.038)	(0.036)	(0.036)	(0.036)	(0.039)	(0.039)	(0.039)
Philanthropy	0.203***	0.203***	0.203***	0.214***	0.214***	0.214***	0.149***	0.149***	0.149***
	(0.037)	(0.037)	(0.037)	(0.036)	(0.036)	(0.036)	(0.038)	(0.038)	(0.038)
Altruism	0.185**	0.185**	0.185**	0.337***	0.337***	0.337***	0.391***	0.391***	0.391***
	(0.067)	(0.067)	(0.067)	(0.064)	(0.064)	(0.064)	(0.069)	(0.069)	(0.069)
Know so. who donated organ	0.095	0.095	0.095	-0.089	-0.089	-0.089	-0.182	-0.182	-0.182
	(0.121)	(0.121)	(0.121)	(0.117)	(0.117)	(0.117)	(0.125)	(0.125)	(0.125)
Know so. who received organ	-0.095	-0.095	-0.095	-0.045	-0.045	-0.045	-0.083	-0.083	-0.083
	(0.095)	(0.095)	(0.095)	(0.091)	(0.091)	(0.091)	(0.097)	(0.097)	(0.097)
Know so. who is critically ill	-0.005	-0.005	-0.005	-0.016	-0.016	-0.016	0.001	0.001	0.001
	(0.111)	(0.111)	(0.111)	(0.107)	(0.107)	(0.107)	(0.115)	(0.115)	(0.115)
Healthy enough	0.193***	0.193***	0.193***	0.192***	0.192***	0.192***	0.122***	0.122***	0.122***
	(0.021)	(0.021)	(0.021)	(0.020)	(0.020)	(0.020)	(0.022)	(0.022)	(0.022)
Trust in doctors	0.150***	0.150***	0.150***	0.193***	0.193***	0.193***	0.134***	0.134***	0.134***
	(0.026)	(0.026)	(0.026)	(0.025)	(0.025)	(0.025)	(0.027)	(0.027)	(0.027)
Registration as deceased donor	0.202*	0.202*	0.202*	0.291***	0.291***	0.291***	0.299***	0.299***	0.299***
	(0.085)	(0.085)	(0.085)	(0.082)	(0.082)	(0.082)	(0.088)	(0.088)	(0.088)
Age	0.004	0.004	0.004	-0.010***	-0.010***	-0.010***	-0.007**	-0.007**	-0.007**
	(0.003)	(0.003)	(0.003)	(0.003)	(0.003)	(0.003)	(0.003)	(0.003)	(0.003)
Gender	-0.304***	-0.304***	-0.304***	-0.035	-0.035	-0.035	-0.024	-0.024	-0.024
	(0.075)	(0.075)	(0.075)	(0.072)	(0.072)	(0.072)	(0.077)	(0.077)	(0.077)
Religious	0.142+	0.142+	0.142+	0.109	0.109	0.109	0.078	0.078	0.078
	(0.073)	(0.073)	(0.073)	(0.070)	(0.070)	(0.070)	(0.075)	(0.075)	(0.075)
Country Austria	0.180*	0.180*	0.180*	-0.177*	-0.177*	-0.177*	-0.373***	-0.373***	-0.373***
	(0.074)	(0.074)	(0.074)	(0.071)	(0.071)	(0.071)	(0.076)	(0.076)	(0.076)

Notes. $N = 1{,}721$. Significance levels: + $P < 0.1$, * $P < 0.05$, ** $P < 0.01$, *** $P < 0.001$. P-values are two-tailed. Estimates (and standard errors in parentheses) reflect the effects of each condition on the DV conditional on controls. To highlight pairwise comparisons between combinations we estimated separate versions of the same model using different levels of the experimental manipulations as the baseline. All covariates are demeaned to facilitate interpretation.

Figure S3.1: *Differences in willingness to become a living organ donor depending on the policy (opt-in, opt-out) and registration rate (15%, 85%).*

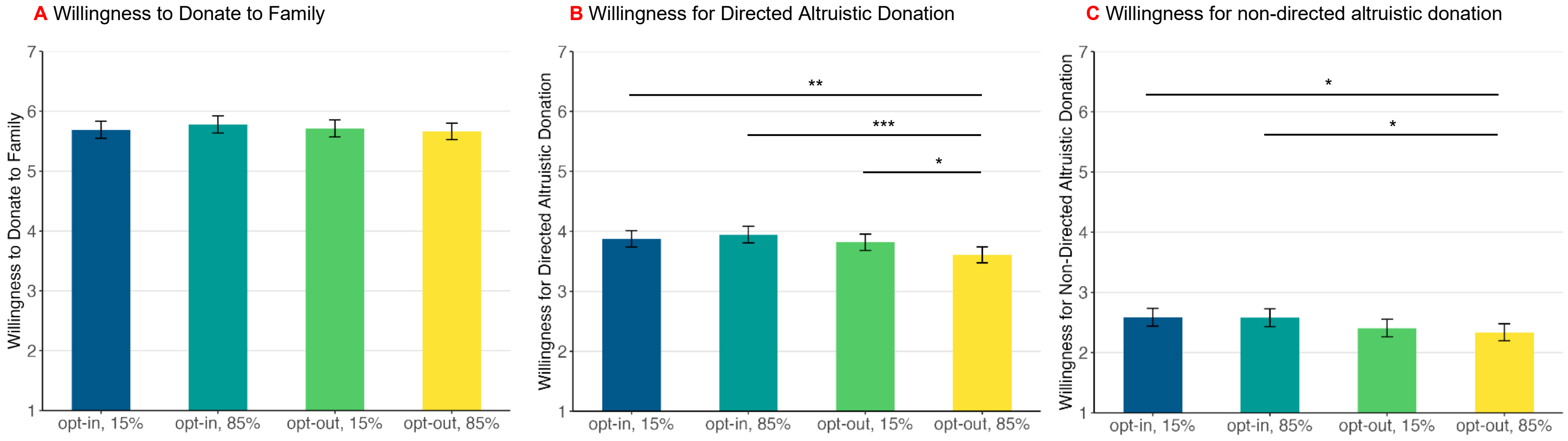

Notes. Significance levels: Significance levels: + $P < 0.1$, * $P < 0.05$, ** $P < 0.01$, *** $P < 0.001$. *P*-values are two-tailed. The plots show differences in willingness to become a familial (Panel A), directed altruistic (Panel B), and non-directed altruistic living organ donor (Panel C) depending on the policy (opt-in, opt-out) and registration rate (15%, 85%). The horizontal lines and stars indicate which conditions are significantly different from one another. The effects associated with the significance levels reflect the difference between two conditions conditional on all covariate effects. To highlight pairwise comparisons between combinations we estimated separate versions of the same model using different levels of the experimental manipulations as the baseline. All covariates are demeaned to facilitate interpretation.

Figure S3.2: *Differences in perceived supply sufficiency and reputational building depending on the policy (opt-in, opt-out) and registration rate (15%, 85%).*

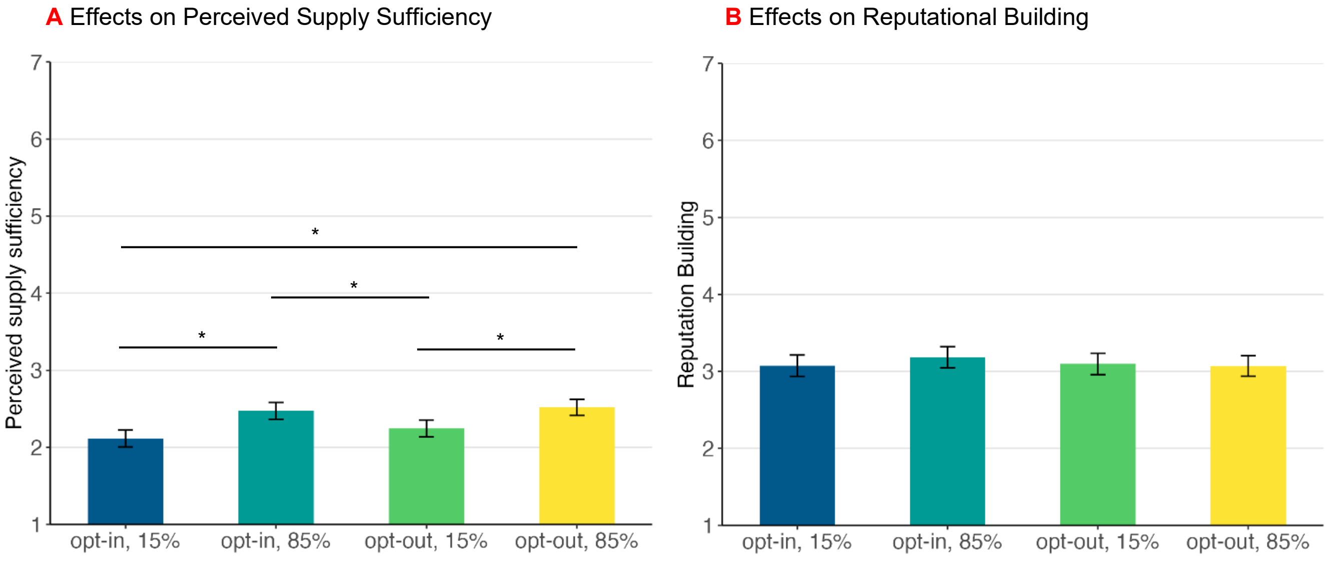

Notes. Significance levels: + $P < 0.1$, * $P < 0.05$, ** $P < 0.01$, *** $P < 0.001$. P-values are two-tailed. The plots show differences in perceptions of supply sufficiency (Panel A) and perceived reputational gain (Panel B) depending on the policy (opt-in, opt-out) and registration rate (15%, 85%). The horizontal lines and stars indicate which conditions are significantly different from one another. The effects associated with the significance levels reflect the difference between two conditions conditional on all covariate effects. To highlight pairwise comparisons between combinations we estimated separate versions of the same model using different levels of the experimental manipulations as the baseline. All covariates are demeaned to facilitate interpretation.

Table S3.5: *Parallel mediation analysis for Study 3.*

	(1) Supply Sufficiency	(2) Supply Sufficiency	(3) Reputation building	(4) Reputation building	(5) Familial donation	(6) Familial donation	(7) Altruistic directed	(8) Altruistic directed	(9) Altruistic non-directed	(10) Altruistic non-directed
Opt-out default policy	0.088<br>(0.055)	0.131+<br>(0.075)	-0.047<br>(0.070)	0.022<br>(0.102)	-0.030<br>(0.071)	0.041<br>(0.103)	-0.188**<br>(0.069)	-0.051<br>(0.098)	-0.205**<br>(0.073)	-0.182+<br>(0.106)
High registration rate	0.315***<br>(0.055)	0.359***<br>(0.078)	0.041<br>(0.069)	0.110<br>(0.099)	0.063<br>(0.071)	0.135<br>(0.102)	-0.057<br>(0.069)	0.081<br>(0.097)	-0.041<br>(0.074)	-0.017<br>(0.103)
Opt-out default policy × high registration rate		-0.086<br>(0.109)		-0.136<br>(0.139)		-0.140<br>(0.141)		-0.269+<br>(0.137)		-0.047<br>(0.147)
Sufficiency of supply					-0.153***<br>(0.035)	-0.154***<br>(0.035)	-0.067*<br>(0.029)	-0.068*<br>(0.029)	-0.013<br>(0.031)	-0.013<br>(0.031)
Reputation building					0.091***<br>(0.026)	0.091***<br>(0.026)	0.126***<br>(0.026)	0.125***<br>(0.026)	0.111***<br>(0.028)	0.111***<br>(0.028)
**Mediation analysis**										
**Specific indirect effects**										
Sum of indirect effects via sufficiency of supply					-0.062**<br>(0.019)	-0.062**<br>(0.019)	-0.027*<br>(0.013)	-0.027*<br>(0.013)	-0.005<br>(0.012)	-0.005<br>(0.012)
Opt-out policy→ Supply Sufficiency→ DV					-0.013<br>(0.009)	-0.020<br>(0.012)	-0.006<br>(0.004)	-0.009<br>(0.006)	-0.001<br>(0.003)	-0.002<br>(0.004)
Registration rate→ Supply Sufficiency→ DV					-0.048**<br>(0.014)	-0.055**<br>(0.018)	-0.021*<br>(0.010)	-0.024*<br>(0.012)	-0.004<br>(0.010)	-0.005<br>(0.011)
Interaction term→ supply sufficiency→ DV						0.013<br>(0.017)		0.006<br>(0.008)		0.001<br>(0.003)
Sum of indirect effects via reputation building					-0.001<br>(0.009)	<0.000<br>(0.009)	-0.001<br>(0.012)	-0.001<br>(0.012)	-0.001<br>(0.011)	<.000<br>(0.009)
Opt-out policy→ reputation building→ DV					-0.004<br>(0.006)	0.002<br>(0.009)	-0.006<br>(0.009)	0.003<br>(0.013)	-0.005<br>(0.008)	0.002<br>(0.011)
Registration rate→ reputation building→ DV					0.004<br>(0.006)	0.010<br>(0.009)	0.005<br>(0.009)	0.014<br>(0.013)	0.005<br>(0.008)	0.012<br>(0.011)
Interaction term→ reputation building→ DV						-0.012<br>(0.013)		-0.017<br>(0.018)		-0.015<br>(0.016)
**Total effects**										
Total indirect					-0.062**<br>(0.021)	-0.063**<br>(0.021)	-0.028<br>(0.018)	-0.028<br>(0.018)	-0.006<br>(0.016)	-0.006<br>(0.016)
Total direct and indirect					-0.030<br>(0.102)	-0.027<br>(0.102)	-0.272**<br>(0.096)	-0.267**<br>(0.096)	-0.252*<br>(0.105)	-0.251*<br>(0.105)

Notes. N = 1,721. Significance levels: + $P < 0.1$, * $P < 0.05$, ** $P < 0.01$, *** $P < 0.001$. P-values are two-tailed. Estimates are unstandardized regression coefficients. We relied on a path regression analysis to estimate all effects simultaneously. All models were estimated with a maximum likelihood estimator with robust standard errors using *M*plus version 8.9. No correction for multiple comparisons was used. We used dummy variable coding to test the effects of the default policy (0 = opt-in, 1 = opt-out) and registration rate (0 = 15%, 1 = 85%) on willingness to donate living organs. Following the pre-registration, we analyzed the effects including the effects of all covariates on each dependent variable to account for the influence of relevant influence factors of organ donation decisions identified by prior literature. Results for covariate effects are not included to facilitate interpretation of the core effects.

Supplementary File 4: Materials and Supporting Analyses for Study 4

Table S4.1: *Survey design, experimental manipulations, and measures.*

**Briefing on organ donation**	
**Introduction to organ donation**	To familiarize you with the topic of **organ donation**, we would like to provide you with some information before you complete the questionnaire.<br>In an organ donation, donors make their own organs available for transplantation to save a recipient's life or improve their quality of life.<br>Every person can freely decide whether they want to donate their organs or not.<br>There are two possible ways to donate organs:<br>• **Deceased organ donation**: In a deceased donation, organs are removed and donated after death.<br>• **Living donation**: In a living donation, organs (e.g., a kidney) are removed and donated during the donor's lifetime through surgery.
**Experimental manipulations of policy (and congruent registration rate) and organ shortages**	
**Opt-in with 40% registration rate, organ shortages absent**	**Imagine you live in a country where the opt-in policy for deceased organ donation is in place**.<br>The **opt-in policy** works as follows: According to this regulation, a person must explicitly give their consent during their lifetime for their organs to be donated after death (e.g., by filling out an organ donor card).<br>Under this regulation, **40% of the population is registered** as organ donors. This means that, in the event of their death, they are considered potential post-mortem organ donors.
**Opt-in with 40% registration rate, organ shortages present**	**Imagine you live in a country where the opt-in policy for deceased organ donation is in place**.<br>The **opt-in policy** works as follows: According to this regulation, a person must explicitly give their consent during their lifetime for their organs to be donated after death (e.g., by filling out an organ donor card).<br>Under this regulation, **40% of the population is registered** as organ donors. This means that, in the event of their death, they are considered potential post-mortem organ donors.<br>**There is a significant shortage of organ donations**: Patients **wait an average of 5 years for a donor kidney** – and one in five patients does not survive this waiting period.
**Opt-out with 99% registration rate, organ shortages absent**	**Imagine you live in a country where the opt-out policy for deceased organ donation is in place**.<br>The **opt-out policy** works as follows: According to this regulation, a person is automatically considered an organ donor after death, unless they have objected during their lifetime (e.g., by deregistering from the organ donor registry).<br>Under this regulation, **99% of the population is registered** as organ donors. This means that, in the event of their death, they are considered potential post-mortem organ donors.
**Opt-out with 99% registration rate, organ shortages present**	**Imagine you live in a country where the opt-out policy for deceased organ donation is in place**.<br>The **opt-out policy** works as follows: According to this regulation, a person is automatically considered an organ donor after death, unless they have objected during their lifetime (e.g., by deregistering from the organ donor registry).<br>Under this regulation, **99% of the population is registered** as organ donors. This means that, in the event of their death, they are considered potential post-mortem organ donors.<br>**There is a significant shortage of organ donations**: Patients **wait an average of 5 years for a donor kidney** – and one in five patients does not survive this waiting period.

**Dependent variables** *(note that the display order of the recipients was randomized)*	
**Willingness to Donate**	**Now, you learn that someone is seriously ill and needs a kidney donation**. You must decide whether you would donate one of your kidneys as a living donor for this person.<br>• If you choose to donate, the person in need would receive a kidney immediately, but you would have to undergo surgery under general anaesthesia.<br>• If you decide against a living donation, the person in need must rely on receiving a kidney from a deceased donor. The availability of a donor kidney depends on the length of the waiting list.<br>When making your decision, keep in mind that: [key facts of manipulation repeated]<br>**How likely are you to donate a kidney as a living donor for this person if the person in need is ___________?**<br>Please answer this question by filling in the blank with each of the following individuals:<br>(1 = Not at all likely, to 7 = Extremely likely)<br>• A close family member?<br>• A close friend?<br>• A remote relative?<br>• An acquaintance?<br>• A stranger?
**Mediating variables** *(note that the sequences of constructs and items was randomized)*	
**Perceived Sufficiency of Supply**<br>**(α = 0. 876)**<br>(Evans and Ferguson 2014)	To what extent to do you agree with the following statements?<br>(1 = strongly disagree, to 7 = strongly agree)<br>• I believe that there are enough people willing to donate their organs after death. Therefore, I do not need to become a living donor.<br>• I believe that the demand for organs is already sufficiently met by the existing number of registered donors.<br>• If I do not make a living donation, it will surely be quite easy to find a donor organ from the pool of deceased donors.
**Injunctive Social Norm**<br>**(α = 0. 760)**<br>(Lemmens et al. 2009)	To what extent to do you agree with the following statements?<br>(1 = strongly disagree, to 7 = strongly agree)<br>• I would feel that I should also be an organ donor after my death.<br>• I believe that other people will expect me to donate my organs after death.<br>• I would feel socially obligated to remain registered as an organ donor.
**Covariates – Prosocial Attitude**	
**Reciprocity**<br>**(α = 0. 663)**<br>(Perugini et al. 2003)	To what extent do you agree with the following statements?<br>(1 = strongly disagree, to 7 = strongly agree)<br>• To help somebody is the best policy to be certain that s/he will help you in the future.<br>• I do not behave badly with others so as to avoid them behaving badly with me.<br>• When I pay someone compliments, I expect that s/he in turn will reciprocate.<br>• I avoid being impolite because I do not want others being impolite with me.
**Philanthropy**<br>**(α = 0. 643)**<br>(Schuyt, Smit, Bekkers 2004)	To what extent do you agree with the following statements?<br>(1 = strongly disagree, to 7 = strongly agree)<br>• We have to leave this world a better place for the next generation.<br>• Society is in danger because people are less concerned about each other nowadays.<br>• The world needs responsible citizens.<br>• I give money to charitable causes, no matter what the government does.
**Altruism**<br>**(α = 0. 799)**	Please tick the relevant box below that indicates how often, if at all, you have actually carried out the following actions.<br>(1 = never, 5 = very often)

(Manzur and Olavarrieta 2021, based on Rushton, Chrisjohn, and Fekken 1981)	• I have given money to a charity.<br>• I have donated goods or clothes to a charity.<br>• I have done voluntary work for a charity.<br>• I have donated blood.<br>• I have helped carry a stranger's belongings (books, parcels, etc.).<br>• I have let a neighbour who I didn't know too well borrow an item of some value to me (e.g. a dish, tools, etc.).<br>• I have offered to help a handicapped or elderly stranger across a street.<br>• I have offered my seat on a bus or train to a stranger who was standing.<br>• I have helped an acquaintance to move households.
**Covariates – Health and Organ Donation**	
**Know so. who donated organ**	• Do you know someone who has donated an organ by transplantation? (0 = no, 1 = yes)
**Know so. who received organ**	• Do you know someone who has received an organ by transplantation? (0 = no, 1 = yes)
**Know so. who is critically ill**	• Is one of your family members or close friends terminally critically ill? (0 = no, 1 = yes)
**Healthy enough**	• Do you feel healthy enough to be able to make a living donation? **(**1 = not at all, to 7 = completely)
**Trust in doctors**	• Do you trust in doctors and their expertise? (1 = not at all, to 7 = completely)
**Registration as deceased donor**	• I am registered as a potential post-mortem organ donor. (0 = no, 1 = yes)
**Covariates – Demographics**	
**Age**	• How old are you? ___ years
**Gender**	• With which gender do you identify? (0 = female or other, 1 = male)
**Religion**	• Do you belong to a church or religious community? (0 = no, 1 = yes)

Notes. In comparison to Study 2 and 3, we reduced the number of items for the prosocial attitude covariates for economic sampling reasons. For the reciprocity and the philanthropy scale, item selection was based on highest factor loadings in Studies 2 and 3, while retaining construct dimensionality. For the altruism scale we turned to an established short version by Manzur and Olavarrieta (2021).

Table S4.2: *Descriptive statistics for each experimental group (n = 1,582).*

Variable	Opt-in with 40%, shortages absent (n=352) Mean (SD)	Opt-in with 40%, shortages present (n=460) Mean (SD)	Opt-out with 99%, shortages absent (n=341) Mean (SD)	Opt-out with 99%, shortages present (n=429) Mean (SD)
**DVs: Willingness to donate**				
Close family member	5.747 (1.614)	5.861 (1.579)	5.601 (1.716)	5.662 (1.720)
Close friend	4.449 (1.845)	4.528 (1.850)	4.308 (1.874)	4.326 (1.896)
Distant relative	3.412 (1.877)	3.604 (1.825)	3.334 (1.749)	3.424 (1.770)
Acquaintance	3.273 (1.832)	3.439 (1.821)	3.235 (1.785)	3.212 (1.789)
Stranger	2.165 (1.549)	2.417 (1.661)	2.117 (1.492)	2.289 (1.592)
All donations (index)	3.809 (1.429)	3.970 (1.447)	3.719 (1.406)	3.783 (1.413)
Altruistic donation (friend, relative, acquaintance, stranger)	3.325 (1.538)	3.497 (1.579)	3.249 (1.506)	3.313 (1.536)
**Mediators**				
Sufficiency of supply	2.519 (1.391)	2.467 (1.360)	3.588 (1.690)	3.077 (1.490)
Injunctive social norm	4.190 (1.592)	4.276 (1.617)	4.498 (1.778)	4.429 (1.696)
**Covariates**				
**Prosocial attitudes**				
Reciprocity	3.714 (1.218)	3.762 (1.262)	3.522 (1.163)	3.630 (1.219)
Philanthropy	5.204 (1.066)	5.203 (1.127)	5.152 (1.102)	5.147 (1.178)
Pure Altruism	2.691 (0.717)	2.746 (0.796)	2.703 (0.742)	2.730 (0.752)
**Health & organ donation**				
Knowing s.o. who donated an organ	0.122 (0.328)	0.135 (0.342)	0.129 (0.336)	0.133 (0.340)
Knowing s.o. who received an organ	0.170 (0.377)	0.189 (0.392)	0.208 (0.407)	0.198 (0.399)
Feeling healthy enough to donate	4.259 (1.881)	4.235 (1.912)	4.349 (1.742)	4.044 (1.867)
Trust in doctors	4.983 (1.477)	4.813 (1.606)	4.856 (1.505)	4.828 (1.503)
Registration as deceased organ donor	0.446 (0.498)	0.413 (0.493)	0.396 (0.490)	0.387 (0.488)
**Demographics**				
Age	44.372 (14.519)	44.480 (14.312)	45.443 (14.561)	46.569 (14.821)
Gender	0.523 (0.500)	0.497 (0.501)	0.499 (0.501)	0.497 (0.501)
Religious	0.412 (0.493)	0.481 (0.500)	0.460 (0.499)	0.494 (0.501)

Notes. Coding of binary variables: gender (0 = female and other, 1 = male), all other binary variables (0 = no, 1 = yes); see Supplementary File 4, Table 5.1 for further details.

We collected the data with the help of a panel provider, and it seems that the attention regarding the question about organ supply was somewhat more difficult to answer, leading to substantial more people being screened out because of failed attention checks in the conditions where shortages were absent. This has caused more dropouts in this group and led to the present imbalance in group sizes. We followed the pre-registration to screen out participants that failed the attention check but also tested a model in which these participants were not excluded. The pattern of results remains stable when including participants that failed the attention check.

Table S4.3: *Main and interaction effects of congruent policy/registration rate and the presence of organ shortages on willingness to make a (i) familial, (ii) directed altruistic, and (iii) non-directed altruistic living organ donation.*

	**(1) Familial donation**	**(2) Familial donation**	**(3) Altruistic directed**	**(4) Altruistic directed**	**(5) Altruistic non-directed**	**(6) Altruistic non-directed**
Opt-out, high%	-0.147+<br>(0.077)	-0.141<br>(0.116)	-0.073<br>(0.072)	-0.027<br>(0.109)	-0.038<br>(0.073)	-0.012<br>(0.107)
Organ shortages	0.114<br>(0.078)	0.119<br>(0.106)	0.120+<br>(0.072)	0.159<br>(0.104)	0.230**<br>(0.073)	0.253*<br>(0.103)
Opt-out, high% × Organ shortages		-0.011<br>(0.155)		-0.080<br>(0.145)		-0.046<br>(0.145)
**Covariates**						
Reciprocity	0.083*<br>(0.037)	0.083*<br>(0.037)	0.162***<br>(0.034)	0.162***<br>(0.034)	0.121**<br>(0.035)	0.121**<br>(0.035)
Philanthropy	0.237***<br>(0.045)	0.237***<br>(0.045)	0.162***<br>(0.037)	0.162***<br>(0.037)	0.024<br>(0.039)	0.024<br>(0.039)
Altruism	0.096<br>(0.064)	0.096<br>(0.064)	0.322***<br>(0.057)	0.322***<br>(0.056)	0.413***<br>(0.057)	0.413***<br>(0.057)
Know so. who donated organ	0.024<br>(0.123)	0.024<br>(0.123)	0.085<br>(0.117)	0.086<br>(0.117)	-0.034<br>(0.124)	-0.034<br>(0.124)
Know so. who received organ	0.075<br>(0.110)	0.075<br>(0.110)	0.031<br>(0.100)	0.030<br>(0.100)	0.020<br>(0.102)	0.019<br>(0.102)
Healthy enough	0.182***<br>(0.027)	0.182***<br>(0.027)	0.185***<br>(0.024)	0.184***<br>(0.024)	0.156***<br>(0.022)	0.156***<br>(0.022)
Trust in doctors	0.093**<br>(0.031)	0.093**<br>(0.031)	0.148***<br>(0.028)	0.149***<br>(0.028)	0.094**<br>(0.027)	0.095**<br>(0.027)
Registration as deceased donor	-0.099<br>(0.081)	-0.099<br>(0.081)	0.229**<br>(0.077)	0.230**<br>(0.077)	0.290***<br>(0.078)	0.290***<br>(0.078)
Age	0.002<br>(0.003)	0.002<br>(0.003)	-0.011***<br>(0.003)	-0.011***<br>(0.003)	-0.007*<br>(0.003)	-0.007*<br>(0.003)
Gender	-0.504***<br>(0.081)	-0.504***<br>(0.081)	-0.087<br>(0.076)	-0.087<br>(0.076)	-0.075<br>(0.077)	-0.074<br>(0.077)
Religious	-0.104<br>(0.080)	-0.105<br>(0.08)	-0.007<br>(0.074)	-0.007<br>(0.074)	0.036<br>(0.074)	0.036<br>(0.074)

Notes. N = 1,582. Significance levels: + $P < 0.1$, * $P < 0.05$, ** $P < 0.01$, *** $P < 0.001$. P-values are two-tailed. Estimates show unstandardized regression coefficients. We relied on a path regression analysis to estimate the effects in the models (1), (3), and (5), as well as (2), (4), and (6) simultaneously. All models were estimated with a maximum likelihood estimator with robust standard errors using *M*plus version 8.9. No correction for multiple comparisons was used. We used dummy variable coding to test the effects of the default policy (0 = opt-in, 1 = opt-out) and organ shortage (0 = absent, 1 = present) on willingness to donate living organs. Directed altruistic donations is an index reflecting the average willingness to give to a friend, acquaintance, and distant relative. Following the pre-registration, we included the covariates in all models.

Figure S4.1: *Differences in willingness to become a familial and altruistic living organ donor depending on the policy and registration rate (opt-in with low%, opt-out with high %) and organ shortages (absent, present).*

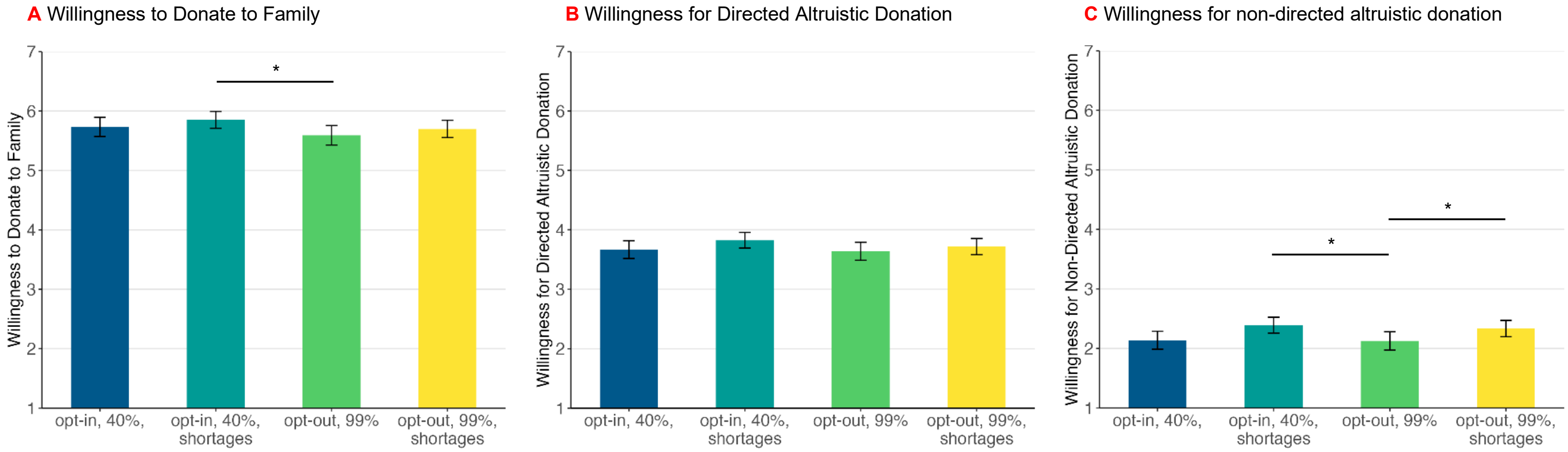

Notes. Significance levels: * $P < 0.05$, ** $P < 0.01$, *** $P < 0.001$. *P*-values are two-tailed. The plots show differences in willingness to become a familial (Panel A), directed altruistic (Panel B), and non-directed altruistic living organ donor (Panel C) depending on the policy (opt-in, 40% vs. opt-out, 99%) and organ shortages (absent vs. present). The horizontal lines and stars indicate which conditions are significantly different from one another. The effects associated with the significance levels reflect the difference between two conditions conditional on all covariate effects. To highlight pairwise comparisons between combinations we estimated separate versions of the same model using different levels of the experimental manipulations as the baseline. All covariates are demeaned to facilitate interpretation.

Figure S4.2: *Differences in perceived supply sufficiency and injunctive social norms depending on the policy and registration rate (opt-in, 40% vs. opt-out, 99%) and mentioning of organ shortages (absent vs. present).*

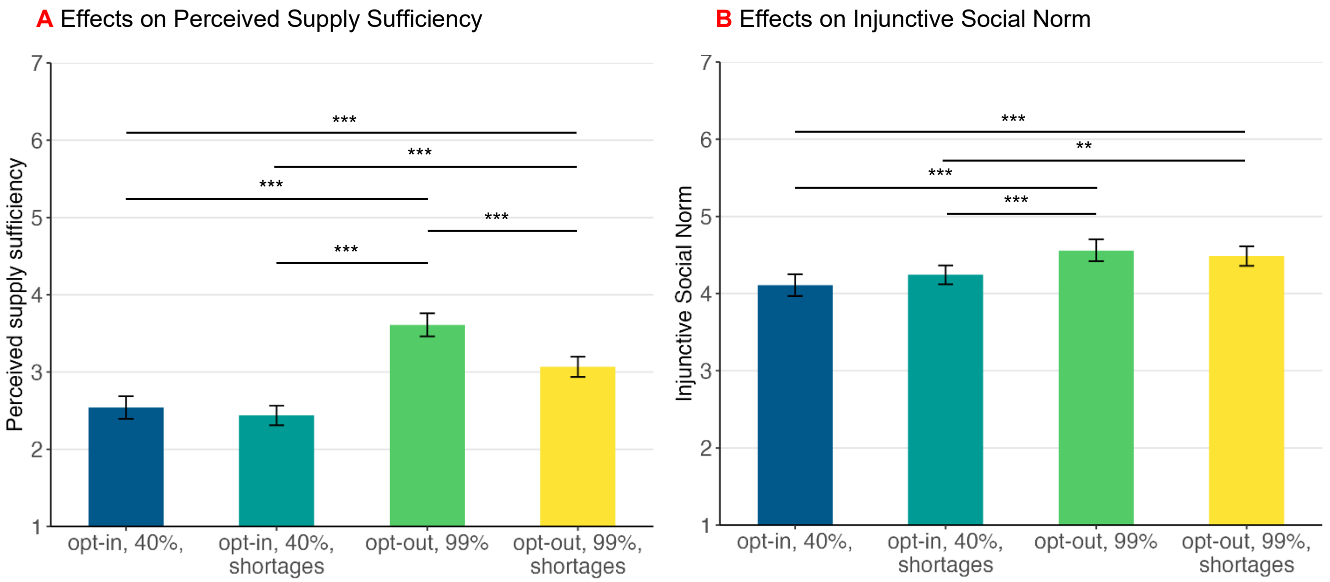

Notes. Significance levels: * $P < 0.05$, ** $P < 0.01$, *** $P < 0.001$. P-values are two-tailed. The plots show differences in perceptions of supply sufficiency (Panel A) and injunctive social norm (Panel B) depending on the policy (opt-in, 40% vs. opt-out, 99%) and organ shortages (absent vs. present). The horizontal lines and stars indicate which conditions are significantly different from one another. The effects associated with the significance levels reflect the difference between two conditions conditional on all covariate effects. To highlight pairwise comparisons between combinations we estimated separate versions of the same model using different levels of the experimental manipulations as the baseline. All covariates are demeaned to facilitate interpretation.

Table S4.4: *Parallel mediation analysis for Study 4.*

Tested relationships	(1) Supply Sufficiency	(2) Supply Sufficiency	(3) Injunctive Social Norm	(4) Injunctive Social Norm	(5) Familial donation	(6) Familial donation	(7) Altruistic directed	(8) Altruistic directed	(9) Altruistic non-directed	(10) Altruistic non-directed
Opt-out default policy	0.823*** (0.070)	1.071*** (0.113)	0.334*** (0.067)	0.452*** (0.104)	-0.050 (0.079)	-0.017 (0.119)	0.022 (0.074)	0.095 (0.110)	0.047 (0.078)	0.099 (0.112)
Shortages present	-0.317*** (0.072)	-0.101 (0.090)	0.030 (0.068)	0.134 (0.090)	0.049 (0.077)	0.078 (0.103)	0.058 (0.071)	0.119 (0.102)	0.186* (0.073)	0.230* (0.102)
Opt-out default policy × shortages present		-0.441** (0.143)		-0.209 (0.136)		-0.058 (0.152)		-0.127 (0.142)		-0.090 (0.145)
Sufficiency of supply					-0.187*** (0.031)	-0.188*** (0.031)	-0.179*** (0.026)	-0.181*** (0.026)	-0.133*** (0.026)	-0.134*** (0.026)
Injunctive social norm					0.169*** (0.034)	0.169*** (0.034)	0.159*** (0.029)	0.158*** (0.029)	0.071* (0.028)	0.071* (0.028)
**Mediation analysis**										
**Specific indirect effects**										
Indirect effect via sufficiency of supply					-0.095*** (0.024)	-0.099*** (0.025)	-0.091*** (0.022)	-0.096*** (0.022)	-0.067*** (0.019)	-0.071*** (0.019)
Opt-out policy→ Supply Sufficiency→ DV					-0.154*** (0.029)	-0.201*** (0.041)	-0.147*** (0.026)	-0.194*** (0.035)	-0.109*** (0.024)	-0.143*** (0.032)
Shortages present→ Supply Sufficiency→ DV					0.059*** (0.016)	0.019 (0.017)	0.057*** (0.015)	0.018 (0.016)	0.042** (0.013)	0.014 (0.012)
Interaction→ Supply Sufficiency→ DV						0.083** (0.031)		0.080** (0.028)		0.059** (0.022)
Indirect effect via injunctive social norm					0.062** (0.020)	0.064** (0.02)	0.058** (0.018)	0.059** (0.019)	0.026* (0.012)	0.027* (0.012)
Opt-out default policy→ Injunctive norms→ DV					0.057*** (0.016)	0.076** (0.023)	0.053*** (0.015)	0.071** (0.021)	0.024* (0.01)	0.032* (0.015)
Shortages present→ Injunctive norms→ DV					0.005 (0.012)	0.023 (0.016)	0.005 (0.011)	0.021 (0.015)	0.002 (0.005)	0.009 (0.007)
Interaction→ Injunctive norms→ DV						-0.035 (0.024)		-0.033 (0.022)		-0.015 (0.011)
**Total indirect effects**										
Total indirect effect					-0.033 (0.030)	-0.036 (0.031)	-0.033 (0.028)	-0.036 (0.028)	-0.041+ (0.022)	-0.044* (0.022)
Total direct and indirect effect					-0.033 (0.111)	-0.033 (0.111)	0.047 (0.102)	0.051 (0.103)	0.192+ (0.103)	0.195+ (0.103)

Notes. N = 1,582. Significance levels: + $P < 0.1$, * $P < 0.05$, ** $P < 0.01$, *** $P < 0.001$. P-values are two-tailed. Estimates are unstandardized regression coefficients. We relied on a path regression analysis to estimate all effects in the main and interaction effects models simultaneously. All models were estimated with a maximum likelihood estimator with robust standard errors using *M*plus version 8.9. No correction for multiple comparisons was used. We used dummy variable coding to test the effects of the default policy (0 = opt-in, 1 = opt-out) and shortages (0 = absent, 1 = present) on willingness to donate living organs. Following the pre-registration, we analyzed the effects including the effects of all covariates on each dependent variable to account for the influence of relevant influence factors of organ donation decisions identified by prior literature. Results for covariate effects are not included to facilitate interpretation of the core effects.

Supplementary File 5: Materials and Supporting Analyses for Study 5

Table S5.1: *Survey design, experimental manipulations, and measures.*

**Briefing on organ donation, default policies, and current organ shortage**	
**Introduction to organ donation**	To familiarize you with the topic of **organ donation**, we would like to provide you with some information before you complete the questionnaire.<br>In an organ donation, donors make their own organs available for transplantation to save a recipient's life or improve their quality of life.<br>Every person can freely decide whether they want to donate their organs or not.<br>There are two possible ways to donate organs:<br>• **Deceased organ donation**: In a deceased donation, organs are removed and donated after death.<br>• **Living donation**: In a living donation, organs (e.g., a kidney) are removed and donated during the donor's lifetime through surgery.
**Briefing on default policies**	There are different approaches countries can take to regulate deceased organ donation.<br>• In Switzerland, the **opt-in policy** currently applies: This means that only those who explicitly consent during their lifetime (e.g., with an organ donor card) are considered organ donors after death.<br>• An alternative is the **opt-out policy**: According to this system, all individuals are automatically considered organ donors after death unless they object during their lifetime (e.g., by unregistering from the organ donor registry).
**Briefing on organ shortage**	**Organ Donation Shortage Under the Opt-In Policy in Switzerland**:<br>Under the currently applicable opt-in policy, there is a significant shortage of organ donations in Switzerland. **The waiting list for a donor kidney is especially long**. Patients wait an average of 2–3 years for a donor kidney – and one in ten patients does not survive the wait.<br>Problem with the opt-in system: Although **56%** of Swiss citizens say they want to donate their organs after death, **only 23% have actually registered**.<br>**To address the shortage of organ donations, significantly more donors are urgently needed**.
**Experimental manipulations of opt-out policy preview**	
**Baseline**	**Introduction of the Opt-Out Policy**<br>To address the problem of the organ donation shortage under opt-in, Switzerland has decided to introduce the opt-out policy, **whereby every person in Switzerland would automatically be considered an organ donor after death**.
**Realistic preview**	**Introduction of the Opt-Out Policy**<br>[same as baseline] + However, the introduction of the opt-out policy has not proven to be an effective solution for increasing the number of deceased organ donors in other countries, as long waiting lists for organs remain. **Therefore, the opt-out policy does not provide a solution to eliminating the organ donation shortage**.
**Optimistic preview**	**Introduction of the Opt-Out Policy**<br>[same as baseline] + Indeed, the introduction of the opt-out system has proven to be an effective solution for increasing the number of post-mortem organ donors in other countries. **Therefore, the opt-out system is often seen as a solution to address the shortage of organ donations**.

**Manipulation checks**	
**Registration rate**	Regardless of your personal opinion on the opt-out default policy ...<br>How many people do you think will be registered as potential organ donors after death under an objection solution?<br>(Slider 0-100%, starting position at current registration level, i.e., 23%)
**Effectiveness**<br>(Hagmann, Ho, and Loewenstein 2019)	How effective do you think the opt-out default policy is compared to the opt-in default in increasing the number of organ donors and solving the problem of organ shortage?<br>(1= much less effective, 7 = much more effective)
**Supply sufficiency**	To what extent do you believe that the supply of donor organs under an opt-out policy will be sufficient to meet the demand for donor organs?<br>(1=not sufficient at all, 7= totally sufficient)
**Dependent variables** *(note that the display order of the recipients was randomized)*	
**Willingness to Donate**	**Imagine that the opt-out policy is already in effect in Switzerland for evaluating the following situation.**<br>Now, you learn that someone is seriously ill and needs a kidney donation. You must decide whether you would donate one of your kidneys as a living donor for this person.<br>• <u>If you choose to donate</u>, the person in need would receive a kidney immediately, but you would have to undergo surgery under general anaesthesia.<br>• <u>If you decide against a living donation</u>, the person in need must rely on receiving a kidney from a deceased donor. The availability of a donor kidney depends on the length of the waiting list.<br>When making your decision, keep in mind that: [key facts of manipulation repeated]<br>**How likely are you to donate a kidney as a living donor for this person if the person in need is ___________?**<br>Please answer this question by filling in the blank with each of the following individuals:<br>(1 = Not at all likely, to 7 = Extremely likely)<br>• A close family member?<br>• A close friend?<br>• A remote relative?<br>• An acquaintance?<br>• A stranger?
**Mediating variables** *(note that the sequences of constructs and items was randomized)*	
**Perceived Sufficiency of Supply**<br>**(α = 0. 834)**<br>(Evans and Ferguson 2014)	To what extent to do you agree with the following statements?<br>(1 = strongly disagree, to 7 = strongly agree)<br>• I believe that there are enough people willing to donate their organs after death. Therefore, I do not need to become a living donor.<br>• I believe that the demand for organs is already sufficiently met by the existing number of registered donors.<br>• If I do not make a living donation, it will surely be quite easy to find a donor organ from the pool of deceased donors.
**Injunctive Social Norm**<br>**(α = 0. 709)**<br>(Lemmens et al. 2009)	To what extent to do you agree with the following statements?<br>(1 = strongly disagree, to 7 = strongly agree)<br>• I would feel that I should also be an organ donor after my death.<br>• I believe that other people will expect me to donate my organs after death.<br>• I would feel socially obligated to remain registered as an organ donor.

Covariates – Prosocial Attitude	
**Reciprocity** **(α = 0. 690)** (Perugini et al. 2003)	To what extent do you agree with the following statements?<br>(1 = strongly disagree, to 7 = strongly agree)<br>• To help somebody is the best policy to be certain that s/he will help you in the future.<br>• I do not behave badly with others so as to avoid them behaving badly with me.<br>• When I pay someone compliments, I expect that s/he in turn will reciprocate.<br>• I avoid being impolite because I do not want others being impolite with me.
**Philanthropy** **(α = 0. 618)** (Schuyt, Smit, Bekkers 2004)	To what extent do you agree with the following statements?<br>(1 = strongly disagree, to 7 = strongly agree)<br>• We have to leave this world a better place for the next generation.<br>• Society is in danger because people are less concerned about each other nowadays.<br>• The world needs responsible citizens.<br>• I give money to charitable causes, no matter what the government does.
**Altruism** **(α = 0. 757)** (Manzur and Olavarrieta 2021, based on Rushton, Chrisjohn, and Fekken 1981)	Please tick the relevant box below that indicates how often, if at all, you have actually carried out the following actions.<br>(1 = never, 5 = very often)<br>• I have given money to a charity.<br>• I have donated goods or clothes to a charity.<br>• I have done voluntary work for a charity.<br>• I have donated blood.<br>• I have helped carry a stranger's belongings (books, parcels, etc.).<br>• I have let a neighbour who I didn't know too well borrow an item of some value to me (e.g. a dish, tools, etc.).<br>• I have offered to help a handicapped or elderly stranger across a street.<br>• I have offered my seat on a bus or train to a stranger who was standing.<br>• I have helped an acquaintance to move households.
**Covariates – Health and Organ Donation**	
**Know so. who donated organ**	• Do you know someone who has donated an organ by transplantation? (0 = no, 1 = yes)
**Know so. who received organ**	• Do you know someone who has received an organ by transplantation? (0 = no, 1 = yes)
**Know so. who is critically ill**	• Is one of your family members or close friends terminally critically ill? (0 = no, 1 = yes)
**Healthy enough**	• Do you feel healthy enough to be able to make a living donation? **(**1 = not at all, to 7 = completely)
**Trust in doctors**	• Do you trust in doctors and their expertise? (1 = not at all, to 7 = completely)
**Registration as deceased donor**	• I am registered as a potential post-mortem organ donor. (0 = no, 1 = yes)
**Covariates – Demographics**	
**Age**	• How old are you? ___ years
**Gender**	• With which gender do you identify? (0 = female or other, 1 = male)
**Religion**	• Do you belong to a church or religious community? (0 = no, 1 = yes)

Notes. In comparison to Study 2 and 3, we reduced the number of items for the prosocial attitude covariates for economic sampling reasons. For the reciprocity and the philanthropy scale, item selection was based on highest factor loadings in Studies 2 and 3, while retaining construct dimensionality. For the altruism scale we turned to an established short version by Manzur and Olavarrieta (2021).

Table S5.2: *Descriptive statistics for each experimental group (n = 1,225).*

Variable	Baseline (n=405) Mean (SD)	Realistic preview (n=411) Mean (SD)	Optimistic preview (n=399) Mean (SD)
**DVs: Willingness to donate**			
Close family member	5.602 (1.566)	5.642 (1.571)	5.637 (1.623)
Close friend	4.533 (1.704)	4.557 (1.705)	4.491 (1.741)
Distant relative	3.363 (1.656)	3.489 (1.649)	3.366 (1.604)
Acquaintance	3.328 (1.739)	3.392 (1.691)	3.281 (1.634)
Stranger	2.299 (1.508)	2.297 (1.517)	2.243 (1.403)
All donations (index)	3.825 (1.336)	3.875 (1.317)	3.804 (1.303)
Altruistic donation (friend, relative, acquaintance, stranger)	3.381 (1.446)	3.434 (1.402)	3.345 (1.369)
**Policy evaluations (manipulation checks)**			
Registration rate	57.815 (22.167)	48.993 (19.719)	56.085 (22.486)
Effectiveness	5.617 (1.359)	4.886 (1.41)	5.519 (1.398)
**Mediators**			
Sufficiency of supply	3.142 (1.422)	2.685 (1.184)	3.001 (1.318)
Injunctive social norm	4.149 (1.521)	4.120 (1.540)	4.089 (1.520)
**Covariates**			
**Prosocial attitudes**			
Reciprocity	3.534 (1.181)	3.570 (1.225)	3.593 (1.251)
Philanthropy	5.178 (1.034)	5.134 (0.995)	5.113 (1.085)
Pure Altruism	2.813 (0.651)	2.805 (0.664)	2.770 (0.647)
**Health & organ donation**			
Knowing s.o. who donated an organ	0.133 (0.340)	0.178 (0.383)	0.128 (0.334)
Knowing s.o. who received an organ	0.254 (0.436)	0.231 (0.422)	0.233 (0.423)
Feeling healthy enough to donate	4.578 (1.743)	4.557 (1.643)	4.516 (1.735)
Trust in doctors	4.817 (1.388)	4.759 (1.431)	4.789 (1.414)
Registration as deceased organ donor	0.289 (0.454)	0.311 (0.464)	0.301 (0.459)
Voted against opt-out	0.175 (0.381)	0.185 (0.389)	0.148 (0.355)
**Demographics**			
Age	43.644 (14.102)	43.745 (14.512)	44.373 (14.872)
Gender	0.516 (0.500)	0.499 (0.501)	0.509 (0.501)
Religious	0.469 (0.500)	0.513 (0.500)	0.516 (0.500)

Notes. Coding of binary variables: gender (0 = female and other, 1 = male), all other binary variables (0 = no, 1 = yes).

Figure S5.1: *Differences in expected registration rate and policy effectiveness depending on description of policy effectiveness.*

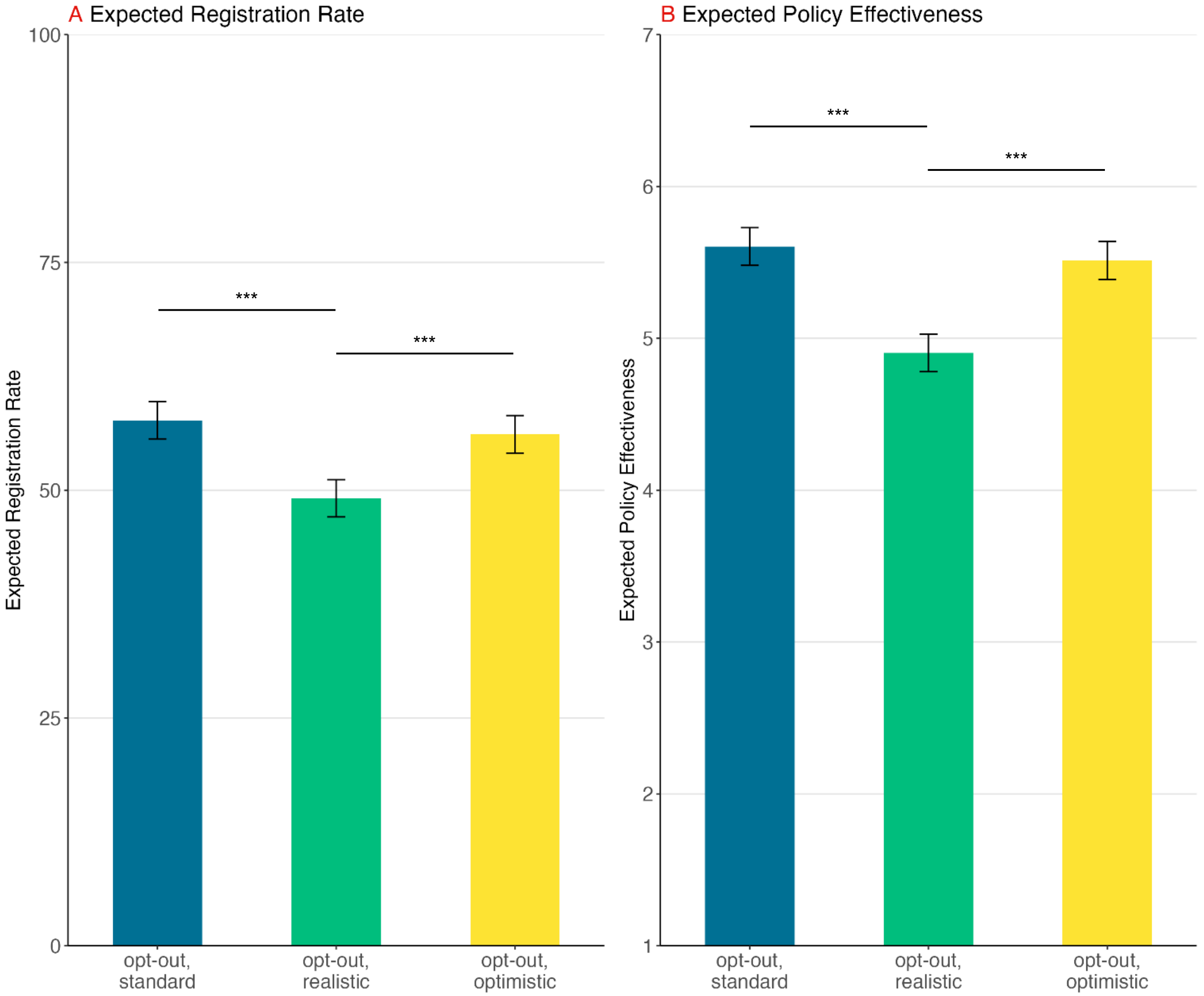

Notes. Significance levels: * $P < 0.05$, ** $P < 0.01$, *** $P < 0.001$. *P*-values are two-tailed. The plots show differences in expected registration rate (Panel A) and policy effectiveness (Panel B) depending on the description of the policy intervention. The horizontal lines and stars indicate which conditions are significantly different from one another. The effects associated with the significance levels reflect the difference between two conditions conditional on all covariate effects. To highlight pairwise comparisons between combinations we estimated separate versions of the same model using different levels of the experimental manipulations as the baseline. All covariates are demeaned to facilitate interpretation.

Figure S5.2: *Differences in willingness to become a familial and altruistic organ donor depending on description of policy effectiveness.*

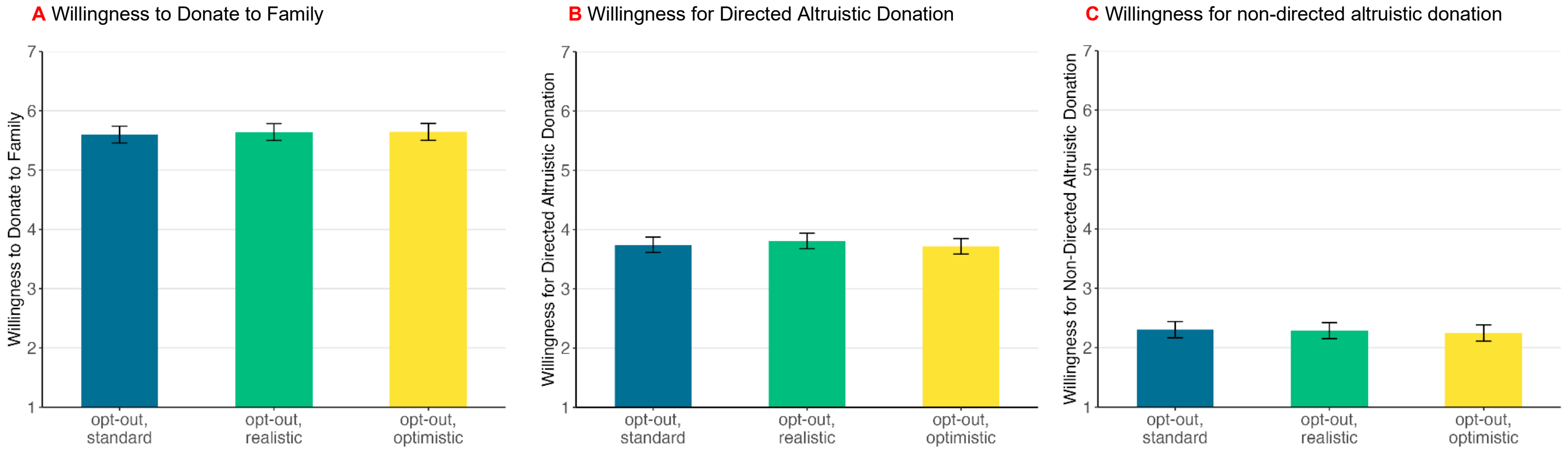

Notes. Significance levels: * $P < 0.05$, ** $P < 0.01$, *** $P < 0.001$. *P*-values are two-tailed. The plots show differences in willingness to become a familial (Panel A), directed altruistic (Panel B), and non-directed altruistic living organ donor (Panel C) depending on whether the effectiveness of the opt-out policy is described as (i) standard, (ii) realistic, or (iii) optimistic. The horizontal lines and stars indicate which conditions are significantly different from one another. The effects associated with the significance levels reflect the difference between two conditions conditional on all covariate effects. To highlight pairwise comparisons between combinations we estimated separate versions of the same model using different levels of the experimental manipulations as the baseline. All covariates are demeaned to facilitate interpretation.

Figure S5.3: *Differences in perceived supply sufficiency and injunctive social norms depending on description of policy effectiveness.*

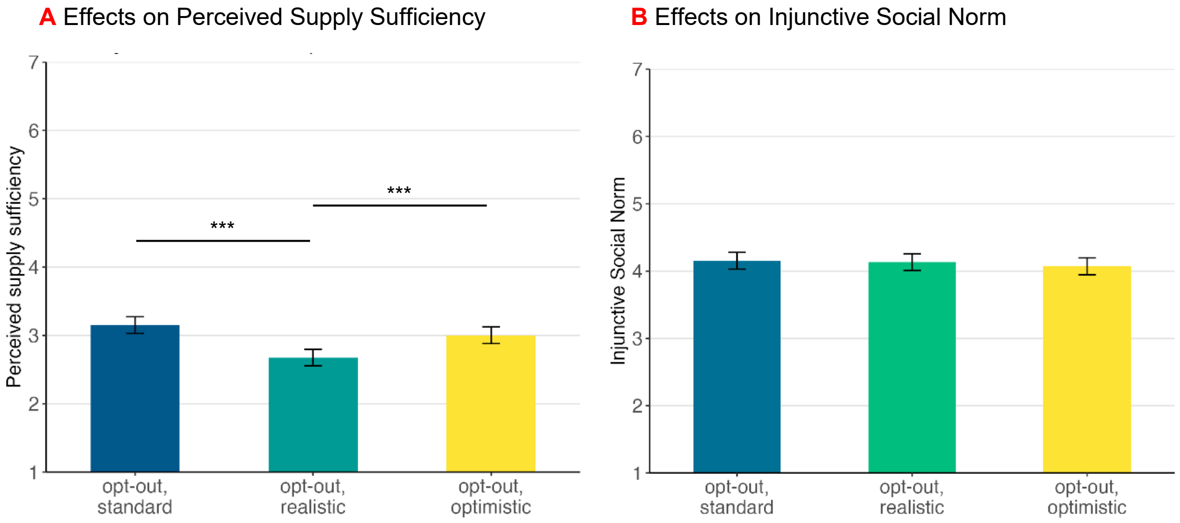

Notes. Significance levels: * $P < 0.05$, ** $P < 0.01$, *** $P < 0.001$. *P*-values are two-tailed. The plots show differences in perceived supply sufficiency (Panel A) and injunctive social norms (Panel B) depending on whether the effectiveness of the opt-out policy is described as (i) standard, (ii) realistic, or (iii) optimistic. The horizontal lines and stars indicate which conditions are significantly different from one another. The effects associated with the significance levels reflect the difference between two conditions conditional on all covariate effects. To highlight pairwise comparisons between combinations we estimated separate versions of the same model using different levels of the experimental manipulations as the baseline. All covariates are demeaned to facilitate interpretation.

Table S5.3: *Parallel mediation analysis for Study 5.*

	(1) Supply Sufficiency	(2) Injunctive Social Norm	(3) Familial donation	(4) Altruistic directed	(5) Altruistic non-directed
Realistic (vs. standard & optimistic)	-0.401***<br>(0.075)	0.020<br>(0.077)	-0.067<br>(0.087)	-0.001<br>(0.08)	-0.053<br>(0.084)
Sufficiency of supply			-0.21***<br>(0.033)	-0.188***<br>(0.03)	-0.153***<br>(0.032)
Injunctive social norm			0.114***<br>(0.032)	0.12***<br>(0.029)	0.102**<br>(0.031)
**Mediation analysis**					
**Specific indirect effects**					
Indirect effect via sufficiency of supply			0.084***<br>(0.021)	0.075***<br>(0.019)	0.061***<br>(0.017)
Indirect effect via injunctive social norm			0.002<br>(0.009)	0.002<br>(0.009)	0.002<br>(0.008)
**Total indirect effects**					
Total indirect effect			0.087***<br>(0.022)	0.078***<br>(0.021)	0.063**<br>(0.019)
Total direct and indirect effect			0.020<br>(0.088)	0.077<br>(0.081)	0.011<br>(0.085)

Notes. N = 1,225. Significance levels: + $P < 0.1$, * $P < 0.05$, ** $P < 0.01$, *** $P < 0.001$. P-values are two-tailed. Estimates are unstandardized regression coefficients. We relied on a path regression analysis to estimate all effects simultaneously. All models were estimated with a maximum likelihood estimator with robust standard errors using *M*plus version 8.9. No correction for multiple comparisons was used. We used dummy variable coding to test the effects of the default policy (0 = opt-in, low%, 1 = opt-out, high%) on willingness to donate living organs. Following the pre-registration, we analyzed the effects including the effects of all covariates on each dependent variable to account for the influence of relevant influence factors of organ donation decisions identified by prior literature. Results for covariate effects are not included to facilitate interpretation of the core effects.

Supplementary File 6: Internal Meta-Mediation-Analysis for Studies 2-4

Table S6.1: *Parallel mediation analysis – pooled data from Study 2-4.*

	(1) Supply Sufficiency	(2) Familial Donation	(3) Directed altruistic	(4) Non-directed altruistic	(5) Familial Donation	(6) Directed altruistic	(7) Non-directed altruistic
**Focal Relationships**							
Opt-out policy, high registration rate	0.686***	-0.090	-0.160**	-0.130	0.020	-0.069	-0.078
	(0.05)	(0.060)	(0.056)	(0.058)	(0.061)	(0.058)	(0.060)
Supply sufficiency					-0.160***	-0.132***	-0.075**
					(0.025)	(0.021)	(0.025)
**Mediation analysis**							
**Indirect effects**							
Indirect effect via sufficiency of supply					-0.109***	-0.091***	-0.052**
					(0.019)	(0.016)	(0.016)
Total direct and indirect effects					-0.090	-0.160**	-0.130*
					(0.060)	(0.056)	(0.058)

Notes. $N = 2{,}894$. Significance levels: + $P < 0.1$, * $P < 0.05$, ** $P < 0.01$, *** $P < 0.001$. *P*-values are two-tailed. Estimates are unstandardized regression coefficients. We relied on a path regression analysis to estimate all effects simultaneously. All models were estimated with a maximum likelihood estimator with robust standard errors using *M*plus version 8.9. No correction for multiple comparisons was used. We used dummy variable coding to test the effects of the default policy (0 = opt-in, low%, 1 = opt-out, high%) on willingness to donate living organs. Following the pre-registration, we analyzed the effects including the effects of all covariates on each dependent variable to account for the influence of relevant influence factors of organ donation decisions identified by prior literature. Results for covariate effects are not included to facilitate interpretation of the core effects.

Supplementary File 7: Transparency Notes – Deviations from Pre-registration

Table S7.1: *Deviations from pre-registration for Study 2 and 3.*

#	Details		Original wording	Deviation description	To what extent is this a deviation from the preregistered plan?	Judgment of impact
**Preregistration deviations**						
1	Type	Methods	Study design: "In addition, 200 respondents will be allocated to the control group with the 'true' situation in the respective country, regarding the consent system and the proportion of registered/not-deregistered donors. In total, we aim to include 1,000 respondents per country."	*This was carried out as planned. However, we subsequently decided to analyze the groups that were exposed to the 'true' situation in each country in a separate study (Study 2), as the comparison with the hypothetical scenarios is not entirely clean experimentally. Thus, the planned 200 respondents in each of the two countries who were exposed to the 'true' situation are now part of Study 2, while the 800 respondents per country who based their answers on the hypothetical scenario are analyzed as part of Study 3.*	Minor	*Positive impact, as this has led to a methodologically cleaner and more correct analysis.*<br>*No impact on findings, as the results remain unaffected. We only changed the structure of the presentation of the results.*
	Reason	Plan not possible				
	Timing	Before data access				
2	Type	Methods	We pre-registered the planned sample size but missed to add a rationale for this in the pre-registration form.	*We added a sample size calculation in the method section of the current manuscript.*	Minor	*Positive impact, as there is now a more elaborate rationale for the choice of the sample size.*
	Reason	Typo/Error				
	Timing	Before data collection				
3	Type	Methods	Data collection procedures: No information on specific countries in which we collected the data.	*This is not actually a deviation, but an error in our initial data collection description, as we missed to specify the countries in which we collected the data in the pre-registration more clearly.*	Minor	*Impact unclear, but we sticked to the initial plan of using the often-cited comparison between Germany and Austria. However, this plan should have been mentioned in the pre-registration.*
	Reason	Typo/Error				
	Timing	Before data access				

4	Type	Methods	Measured variables: "Our key dependent variable is (1) willingness to be a living organ donor. We ask participants about their willingness to donate a kidney, a lobe of their liver to a stranger, relative or friends on a 7-point Likert scale ranging from 1 not at all likely to 7 extremely likely."	*We continued to pursue this objective as planned but added two categories to better capture differences in emotional and genetic closeness to the recipients. Specifically, we subdivided the category 'relative' into 'close family member' and 'distant relative' and the category 'friend' into 'close friend' and 'acquaintance'.*<br>*The mistake was not to include this change in a new pre-registration.*	Minor	*Positive impact, as this has led to more nuanced insights.*
	Reason	Typo/Error				
	Timing	Before data collection				
5	Type	Methods	Furthermore, background information and demographic controls are measured: knowledge about organ donation, donation, transplantation in social network, trust in medical system, subjective health, age, gender, religious and ethnic background.	*We have relabelled some of the covariates to make them more self-explanatory. Specifically, we use "Know so. who donated organ", "Know so. who received organ", Know so. who is critically ill", "Healthy enough to donate an organ", "Trust in doctors (medical system)", "Registration as deceased donor", "age", "gender", religious".*<br>*We did not measure ethnic background as this question was considered ethically questionable by participants in a pre-test. Moreover, standard US-centric ethnicity categories do not apply to the sampled populations.*	Minor	*No impact on findings of variable relabelling. We do not assume that the ethnic background would make a difference to the results.*
	Reason	Miscommunication				
	Timing	Before data collection				
6	Type	Analyses	Statistical models: "Our key dependent variables are willingness to make a living kidney or liver lobe donation to a friend, stranger or relative and intention to register or de-register as posthumous donor under the different conditions."	*We focused on willingness to make a living donation as the main DV and dropped intention to register or de-register from the model as 1) it is not part of our central hypothesis and 2) has methodological issues (i.e., it captures very different things depending on the default policy condition and on whether one is already (de-)registered or not).*	Minor	*Positive impact, as the experimental setup is cleaner, and the results are tied closer to our main hypothesis.*
	Reason	Peer review				
	Timing	After results known				

Notes. This table was created using the template by Willroth and Atherton (2024) to transparently document any deviations from the initial pre-registration. The pre-registration is accessible under https://osf.io/s4zv6.

Table S7.2: *Deviations from pre-registration for Study 4.*

**Preregistration deviations**							
#	Details		Original wording	Deviation description	To what extent is this a deviation from the preregistered plan?	Judgment of impact
1	Type	Research questions / hypotheses	Hypotheses: "- in the opt-out policy with 99% registration rate in which organ shortages are emphasized a) perceived supply sufficiency will be highest and b) willingness to make an altruistic living organ donation will be lowest."	*We just realized that there was missing word here when going back to the hypotheses before accessing the data. In line with our previous predictions, this should have been:* "- in the opt-out policy with 99% registration rate in which organ shortages are not emphasized a) perceived supply sufficiency will be highest and b) willingness to make an altruistic living organ donation will be lowest."	Minor	*None.*
	Reason	Typo/Error				
	Timing	Before data access				

Notes. This table was created using the template by Willroth and Atherton (2024) to transparently document any deviations from the initial pre-registration. The pre-registration is accessible under https://osf.io/eb84s.

References

Bagozzi, R. P., & Yi, Y. (2012). Specification, evaluation, and interpretation of structural equation models. *Journal of the academy of marketing science*, *40*, 8-34.

Chell, K., & Mortimer, G. (2014). Investigating online recognition for blood donor retention: An experiential donor value approach. *International Journal of Nonprofit and Voluntary Sector Marketing*, *19*(2), 143-163.

Evans, R., & Ferguson, E. (2014). Defining and measuring blood donor altruism: a theoretical approach from biology, economics and psychology. *Vox sanguinis*, *106*(2), 118-126.

Fornell, C., & Larcker, D. F. (1981). Evaluating structural equation models with unobservable variables and measurement error. *Journal of marketing research*, *18*(1), 39-50.

Lemmens, K. P., Abraham, C., Ruiter, R. A., Veldhuizen, I. J., Dehing, C. J., Bos, A. E., & Schaalma, H. P. (2009). Modelling antecedents of blood donation motivation among non-donors of varying age and education. *British journal of psychology*, *100*(1), 71-90.

Manzur, E., & Olavarrieta, S. (2021). The 9-SRA scale: A simplified 9-items version of the SRA scale to assess altruism. *Sustainability*, *13*(13), 6999.

Marsh, H. W., Wen, Z., & Hau, K. T. (2004). Structural equation models of latent interactions: evaluation of alternative estimation strategies and indicator construction. *Psychological methods*, *9*(3), 275.

NHS Blood and Transplant, https://www.odt.nhs.uk/statistics-and-reports/annual-activity-report. [last accessed 05/01/2025].

Perugini, M., Gallucci, M., Presaghi, F., & Ercolani, A. P. (2003). The personal norm of reciprocity. *European Journal of Personality*, *17*(4), 251-283.

Rushton, J. P., Chrisjohn, R. D., & Fekken, G. C. (1981). The altruistic personality and the self-report altruism scale. *Personality and individual differences*, *2*(4), 293-302.

Schuyt, T. N. M., Smit, J., & Bekkers, R. (2004). Constructing a philanthropy-scale: Social responsibility and philanthropy. 33d Arnova Conference. *Los Angeles*.

Willroth, E. C., & Atherton, O. E. (2024). Best laid plans: A guide to reporting preregistration deviations. *Advances in Methods and Practices in Psychological Science*, *7*(1), 25152459231213802.
